# A tight binding and $\overrightarrow{{\boldsymbol{k}}}\cdot \overrightarrow{{\boldsymbol{p}}}$ study of monolayer stanene

**DOI:** 10.1038/s41598-017-12281-y

**Published:** 2017-09-21

**Authors:** Liming Jiang, Paolo Marconcini, Md Sharafat Hossian, Wanzhi Qiu, Robin Evans, Massimo Macucci, Efstratios Skafidas

**Affiliations:** 1 Centre for Neural Engineering, The University of Melbourne, 203 Bouverie St, Carlton, VIC 3053 Australia; 2 Dipartimento di Ingegneria dell’Informazione, Università di Pisa, via G. Caruso 16, 56122 Pisa, Italy; 3 Department of Electrical and Electronic Engineering, The University of Melbourne, Parkville, VIC 3010 Australia; 4 Data61, CSIRO/NICTA, Docklands, VIC 3008 Australia; 5 ARC Research Hub for Graphene Enabled Industry Transformation, The University of Melbourne, Parkville, Victoria Australia

## Abstract

Stanene is a single layer of tin atoms which has been discovered as an emerging material for quantum spin Hall related applications. In this paper, we present an accurate tight-binding model for single layer stanene near the Fermi level. We parameterized the onsite and hopping energies for the nearest, second nearest, and third nearest neighbor tight-binding method, both without and with spin orbital coupling. We derived the analytical solution for the $\overrightarrow{{\boldsymbol{\Gamma }}}$and $\overrightarrow{{\boldsymbol{K}}}$ points and numerically investigated the buckling effect on the material electronic properties. In these points of the reciprocal space, we also discuss a corresponding $\overrightarrow{{\boldsymbol{k}}}\cdot \overrightarrow{{\boldsymbol{p}}}$ description, obtaining the value of the $\overrightarrow{{\boldsymbol{k}}}\cdot \overrightarrow{{\boldsymbol{p}}}$ parameters both analytically from the tight-binding ones, and numerically, fitting the ab-initio dispersion relations. Our models provide a foundation for large scale atomistic device transport calculations.

## Introduction

2 dimensional (2-D) materials demonstrate many unique properties and potentially can be used in many applications to provide better performance in electronics, bio-sensing and spintronics^1^. Recently, another 2-D material which is made up of a single layer of tin atoms, namely stanene, has been theoretically studied^2^ and experimentally synthesized^3^. The properties of stanene have been investigated by means of first principles and it has been predicted to be a large bandgap topological insulator, which makes it a prospective candidate for the implementation of quantum spin-Hall based devices.

A simple yet accurate model is required for large scale stanene based device simulation. Most approaches today implement density functional theory (DFT) to study the electronic properties of stanene^2,4–6^. This method provides a relatively accurate description of the electronic properties of stanene but is computationally expensive. Tight-binding (TB) modeling is a highly scalable method, the complexity of which can be tuned according to the purpose of the study by choosing the number of basis sets and hopping neighbors. In order to model the electronic properties of stanene, here we implement a tight-binding method, which can provide accurate results in the low-energy range but at a significantly lower computational cost. This will provide important advantages for the simulation of complex devices such as sensors and transistors.

In this study, we use the *sp* ^3^ basis set and we investigate the difference in bandstructure of nearest neighbor tight binding (NNTB), second nearest neighbor tight binding (2NTB), and third nearest tight binding (3NTB) models. We found that, in order to fit the DFT bandstructure, an additional parameter has to be inserted into the NNTB method. Both 2NTB and 3NTB models can well reproduce the stanene bandstructure after tuning the on-site and hopping energy parameters. The 3NTB method provides more accurate results for stanene ribbon devices. Our study demonstrates that a tight bind model with properly tuned parameters can accurately describe the stanene properties in the low energy region at a less demanding computational cost, which can be useful for modeling the properties of large devices. A further simplification can be achieved adopting a $\overrightarrow{k}\cdot \overrightarrow{p}$ model for stanene. Here we report the $\overrightarrow{k}\cdot \overrightarrow{p}$ Hamiltonians in the $\overrightarrow{{\rm{\Gamma }}}$, $\overrightarrow{K}$ and $\overrightarrow{K}^{\prime}$ points of the reciprocal space, deriving the $\overrightarrow{k}\cdot \overrightarrow{p}$ parameters both from the tight-binding Slater-Koster parameters, and by directly fitting the DFT bands.

The present paper is organized as follows. We describe the tight binding theory for the stanene nanostructure in section II. In section III, we describe the parameter fitting process we adopt in order to get the correct stanene bandstructure representation. In section IV we present and discuss our tight-binding results for unconfined stanene. In section V we report the $\overrightarrow{k}\cdot \overrightarrow{p}$ Hamiltonians that we can derive from our NNTB description. In section VI we use our tight-binding model to find the dispersion relations in the presence of lateral confinement and of an orthogonal magnetic field. Finally, we draw our conclusions in section VI. The details of the tight-binding and $\overrightarrow{k}\cdot \overrightarrow{p}$ models are provided in the Appendices A and B, respectively.

## Crystal structures and tight-binding Hamiltonian model

**Figure 1:**
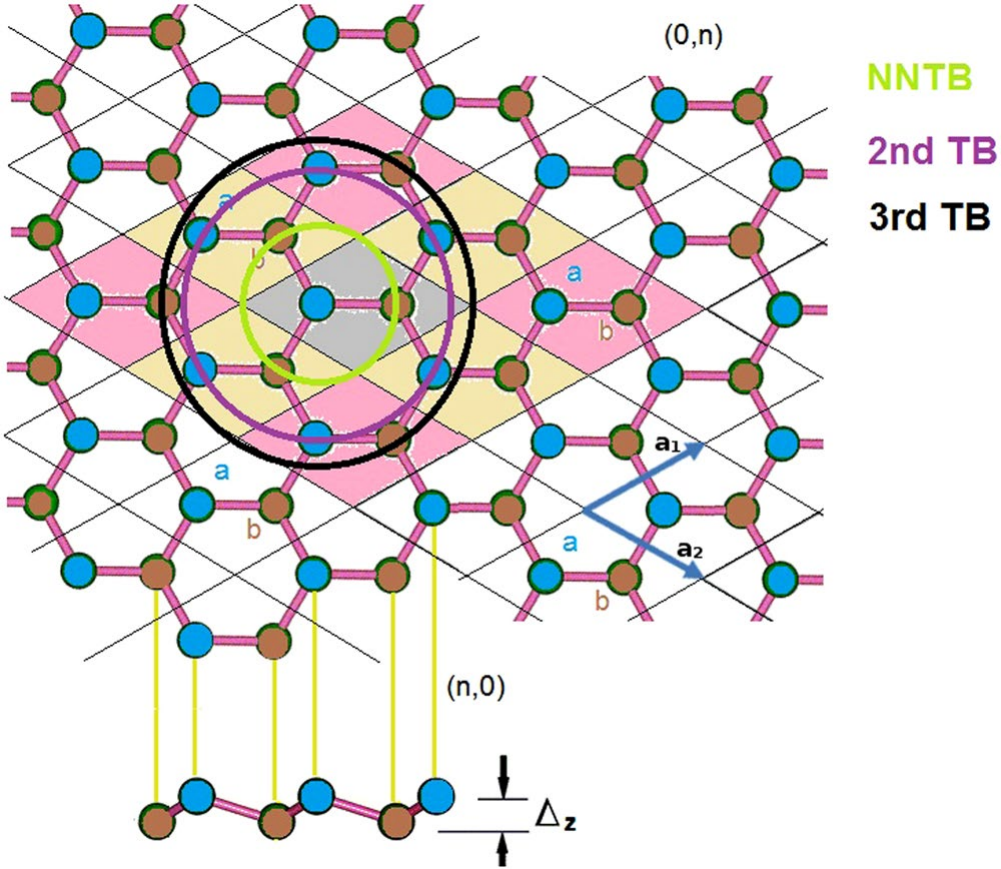
The crystal structure of monolayer stanene.

Stanene is a mono-layer of tin atoms that are arranged in a honeycomb pattern. Unlike graphene, in which all the atoms are aligned in the same plane, the atoms of stanene are buckled in the direction perpendicular to the lattice plane. This is shown in Fig. 1. The lattice coordinates are defined as:

1 $$\begin{array}{c}{\overrightarrow{a}}_{1}={a}_{0}(\frac{\sqrt{3}}{2},\frac{1}{2},\,0);\\ {\overrightarrow{a}}_{2}={a}_{0}(\frac{\sqrt{3}}{2},-\frac{1}{2},\,0);\\ {\overrightarrow{a}}_{3}=(0,\,0,\,{L}_{z})\end{array}$$

where *a*_0_ represents the lattice constant of stanene. *L*_*z*_ represents the unit cell size in the z direction. In our calculation, we set *L*_*z*_ = 20 Å in order to reduce the inter-layer interaction. The buckling distance along the z direction for stanene is set as Δ_*z*_. By optimizing the geometry using DFT methods, we obtain *a*_0_ = 4.698 Å and Δ_*z*_ = 0.86 Å. Based on the lattice coordinates, we can also derive the corresponding reciprocal lattice vectors:

2 $$\begin{array}{c}{\overrightarrow{b}}_{1}=\frac{2\pi }{{a}_{0}}(\frac{\sqrt{3}}{3},-1,\,0);\\ {\overrightarrow{b}}_{2}=\frac{2\pi }{{a}_{0}}(\frac{\sqrt{3}}{3},\,1,\,0);\\ {\overrightarrow{b}}_{3}=\frac{2\pi }{{L}_{z}}(0,\,0,\,1).\end{array}$$

Stanene has a hexagonal Brillouin zone. The most important high symmetry points are $\overrightarrow{{\rm{\Gamma }}}$, $\overrightarrow{M}$ and $\overrightarrow{K}$, which are:

3 $$\begin{array}{ccc}\overrightarrow{{\rm{\Gamma }}} & = & (0,\,0,\,0);\\ \overrightarrow{M} & = & \frac{\pi }{{a}_{0}}(\frac{\sqrt{3}}{3},\,1,\,0);\\ \overrightarrow{K} & = & \frac{2\pi }{{a}_{0}}(\frac{1}{\sqrt{3}},\frac{1}{3},\,0).\end{array}$$

The *sp* ^3^ basis set is commonly used for the electronic property calculations of face centered cubic (FCC) semiconductors, such as silicon^7^ and other zinc blend crystal materials^8^. As a Sn atom has a similar valence shell as Si and C, we used the *sp* ^3^ model to investigate the stanene properties. We denote our basis sets for each tin atom as:

4 $$|{\overrightarrow{r}}_{i};s\rangle ,\,|{\overrightarrow{r}}_{i};{p}_{x}\rangle ,\,|{\overrightarrow{r}}_{i};{p}_{y}\rangle ,\,|{\overrightarrow{r}}_{i};{p}_{z}\rangle $$

where the vector ${\overrightarrow{r}}_{i}$ represents the spatial coordinates and *s*, *p*_*x*_, *p*_*y*_, *p*_*z*_ represent the valence orbitals. The orbitals are represented by Löwdin functions, so they are all orthogonal to each other. From this, we can then construct the stanene Hamiltonian using the tight binding method.

A generalized tight binding Hamiltonian is given by the expression:

5 $$\langle {\overrightarrow{r}}_{i};n|\hat{H}(\overrightarrow{k})|{\overrightarrow{r}}_{j};m\rangle =\sum _{l\in L}\,{V}_{n,m}^{l}\cdot {e}^{i\overrightarrow{k}\cdot ({\overrightarrow{r}}_{i}-{\overrightarrow{r}}_{j})}$$

where *n*, *m* are integers that index all the basis sets in the defined system. The term ${V}_{n,m}^{l}$ represents the corresponding on-site energy or two center integral between the basis elements *n*, *m* as proposed by Slater and Koster^9^ and *L* is a set that contains all the sites that we consider in the calculation.

We implement three methods, including the nearest neighbor tight binding (NNTB), the second nearest neighbor tight binding (2NTB) and the third nearest neighbor tight binding (3NTB) model, to represent the bandstructure of stanene. For the NNTB model, we use 2 on-site energies, 4 hopping energy terms and 1 additional fitting parameter, represented by *ε*_*s*_, *ε*_*p*_ for the on-site energies, (*ssσ*), (*spσ*), (*ppσ*), (*ppπ*) for the hopping terms, and Δ_*pz*_ as an additional fitting parameter^10^ that we add to the on-site energy of *p*_*z*_ orbital. We further expanded our tight binding model to the second (2NTB) and third nearest neighbors (3NTB). The 2NTB model contains 10 parameters, which include 2 onsite energies, 4 nearest hopping energies and 4 second nearest neighbor hopping energies. For the 3NTB, there are 14 parameters which include the 2 parameters for on-site energies, 4 parameters for nearest neighbor hopping, 4 parameters for 2nd nearest neighbor hopping, (*ssσ*_2_), (*spσ*_2_), (*ppσ*_2_), (*ppπ*_2_), and 4 parameters for the 3rd nearest neighbor hopping, denoted by (*ssσ*_3_), (*spσ*_3_), (*ppσ*_3_), (*ppπ*_3_). Determining these energy values is critical for the tight binding approximation. Some methods exist for the tight binding parametrization, including the free electron approximation^11^, data fitting^12,13^ and ab initio projection^14^. There are also tight binding parameter tables available for certain lattice configuration, e.g. Vogl *et al*.^8^ and Grosso and Piermarocchi^15^. Although these, ready made, parameters work well in producing the bandstructure for the bulk configuration of materials, they are not quite adequate for the representation of 2-dimensional materials because they are derived for the zinc blend lattice configuration. Thus these parameters should be modified to account for the 2 dimensional geometry configuration. On the other hand, these parameters can be a good initial starting point for the data fitting method. In our tight binding parametrization, we use Vogl’s parameters as an initial starting point, and fit the tight binding model to the DFT calculated bandstructure.

The first-principle DFT calculation acts as a reference for our tight-binding modeling. DFT calculations were performed using quantum espresso^16^. We used the pseudopotential *Sn*.*pz*-*bhs*.*UPF* for the calculation without spin-orbital interaction and used the pseudopotential *Sn*.*rel*-*pz*-*dn*-*kjpaw*_*psl*.0.2.*UPF* for the calculations with spin-orbital interaction. The unit cell contains two Sn atoms with an optimized lattice constant of 4.698 Å. The lattice unit cell size in the z direction is 20 Å. This is chosen so as to eliminate the interaction between different layers. The Brillouin zone is sampled using a 10 × 10 × 1 mesh.

## Parameter fitting

In tight binding calculations, the appropriate determination of related on-site and hopping energies is essentially important for the correct representation of the material bandstructure. Here, the data fitting method^12,13^ is a very convenient way to get on-site and hopping parameters by minimizing the mean square error between the bandstructure predicted by our model and that from the DFT calculation. Furthermore, by adjusting the weight of certain $\overrightarrow{k}$ vectors, we can control the fitting accuracy of the bandstructure at certain $\overrightarrow{k}$ points in the reciprocal space. The cost function can be defined as:

6 $$c(\overrightarrow{k})=\sum _{\overrightarrow{k},j}\,{w}_{j}(\overrightarrow{k})\,{({E}_{j}^{TB}(\overrightarrow{k})-{E}_{j}^{DFT}(\overrightarrow{k}))}^{2}$$

where the ${w}_{j}(\overrightarrow{k})$ represents the weight in a certain region of the Brillouin zone, with band index *j*. ${E}_{j}^{TB}$ and ${E}_{j}^{DFT}$ represent the dispersion relationships, with the subscript indicating the band index. Depending on how the optimization algorithm is implemented, the choice of the initial starting point can be important because the complete fitting might cause the cost function to fall into a local minimum. To avoid this, we used Vogl’s parameters as the starting point to fit our tight binding model. Since the 2-D material is just a layer of a 3-D lattice configuration, correlation between the 2-D TB parameters and the 3-D TB parameters is expected.

## Tight-binding dispersion relations

### Stanene bandstructure

**Figure 2:**
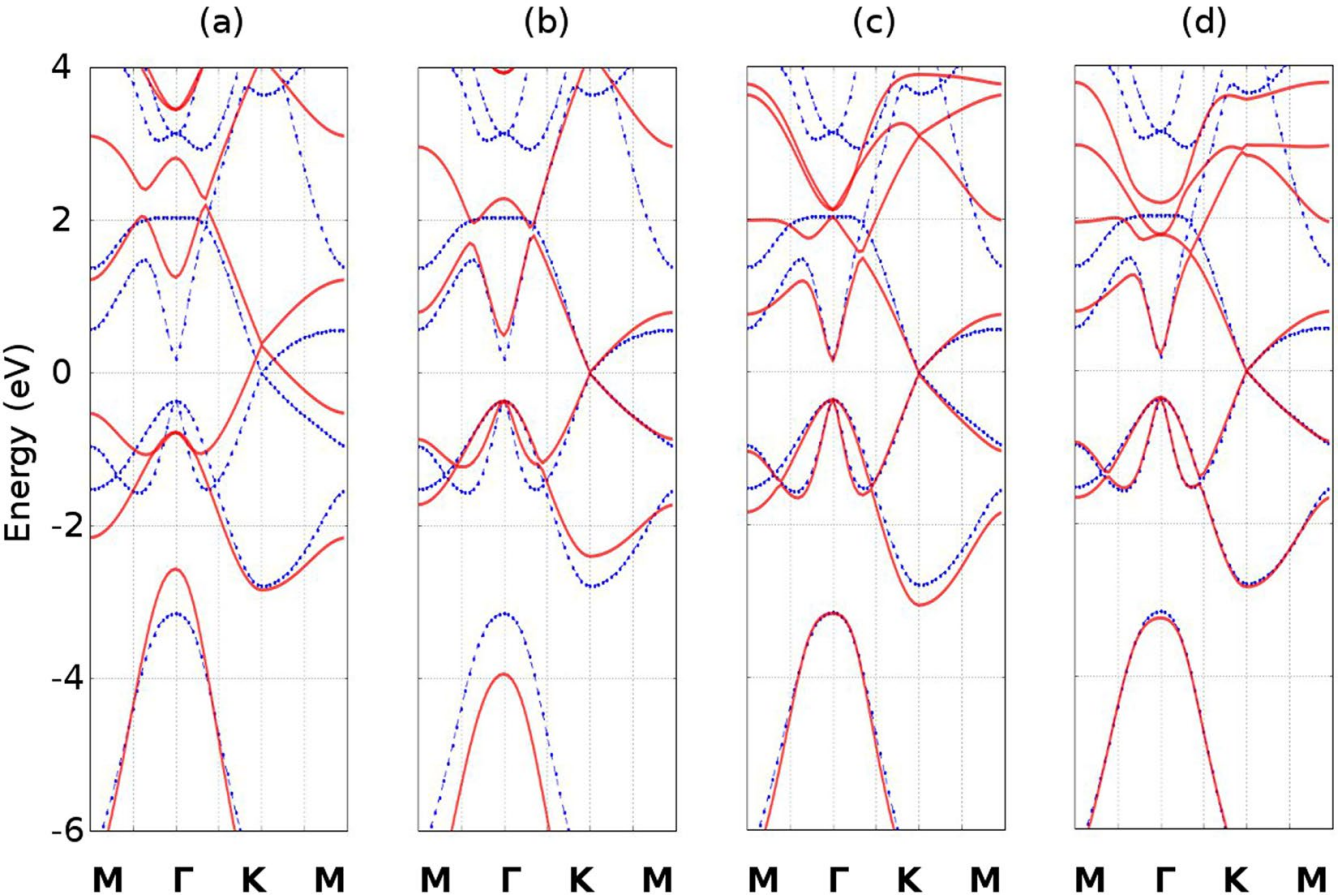
Monolayer stanene bandstructure comparison between the DFT and our tight binding model. The blue dotted lines represent the bandstructure calculated by means of DFT without spin orbital interaction, whereas the red solid lines represent the bandstructure calculated by means of our tight binding model. (**a**) represents the bandstructure calculated using Vogl’s parameters. (**b**) represents the bandstructure calculated with the NNTB with additional fitting parameter. (**c**) and (**d**) represent the bandstructure calculated with the 2NTB and 3NTB method, respectively.

The bandstructure for mono-layer stanene is presented in Fig. 2. The dotted blue lines are the DFT calculation results, whereas the red solid lines represent the bandstructure produced by our tight binding model. As can been seen from Fig. 2(a), the bandstructure calculated by means of Vogl’s parameters shows a large difference from to the first principle result. Hence it is not well suited for a 2-D material such as stanene and cannot correctly predict its energy diagram. Figure 2(b) represents the best coincidence fit bandstructure for the NNTB model with 1 additional parameter which accounts for the on-site energy difference between *p*_*z*_ and *p*_*x*,*y*_^17^. We mainly fitted our model to account for all the conduction bands and the first and second valence bands, and added more weight to the high symmetry points in order to get the most critical values correct. The 2NTB and 3NTB can both give a better representation for bands close to and below the Fermi level. However, the 3NTB overall can fit the low energy region more accurately than the 2NTB. Our model provides a reasonable good match for the high-symmetry points $\overrightarrow{M},\,\overrightarrow{{\rm{\Gamma }}},\,\overrightarrow{K}$ and it is suitable for the study of the low energy properties of stanene. The parameters for the on-site and hopping energies are summarized in Table 1.

**Table 1:** The initial energy values of stanene two center integral parameters taken from Vogl *et al*.^8^ and the parameter fitted in our calculation.

Parameter	Vogl *et al*.	NNTB	2NTB	3NTB
Δ_*pz*_	—	−0.946	—	—
*ε*_*s*_	−5.6700	−6.4042	−5.2441	−5.1576
*ε*_*p*_	1.3300	1.7747	0.5675	0.4728
(*ssσ*)	−1.4175	−1.2154	−1.2487	−1.2531
(*spσ*)	1.9536	1.9539	1.8252	1.8809
(*ppσ*)	2.3725	2.3851	1.8018	1.5222
(*ppπ*)	−0.6875	−0.6769	−0.7443	−0.7384
(*ssσ*_2_)	—	—	−0.0374	−0.0496
(*spσ*_2_)	—	—	−0.0626	−0.0358
(*ppσ*_2_)	—	—	0.1575	0.1020
(*ppπ*_2_)	—	—	−0.0555	−0.0236
(*ssσ*_3_)	—	—	—	0.0537
(*spσ*_3_)	—	—	—	0.0507
(*ppσ*_3_)	—	—	—	0.1341
(*ppπ*_3_)	—	—	—	−0.0010

All the energy values are in eV.

### Spin orbital interaction

For a better representation of the stanene bandstructure, we included the spin orbital coupling in our study. The general form of the spin orbit interaction Hamiltonian is usually written as:

7 $$\hat{H}=\frac{e\hslash }{4{m}^{2}{c}^{2}}\overrightarrow{\hat{\sigma }}\cdot (\overrightarrow{E}\times \overrightarrow{\hat{p}})$$

where $\overrightarrow{\hat{\sigma }}$ represents the Pauli spin matrices including $({\hat{\sigma }}_{x},\,{\hat{\sigma }}_{y},\,{\hat{\sigma }}_{z})$. $\overrightarrow{E}$ is the vector that represents the electrical field of an atomic nucleus and $\overrightarrow{\hat{p}}$ represents the momentum operator. Nonzero spin-orbit interaction exists only between orbitals on the same atom. Due to the symmetry of the *s* orbital, all the on site spin-orbital interaction terms with the *s* orbital become zero. Therefore we got a spin-orbit coupling matrix in the span of basis sets (*p*_*x*↑_, *p*_*y*↑_, *p*_*z*↑_, *p*_*x*↓_, *p*_*y*↓_, *p*_*z*↓_):

8 $${H}_{so}=\frac{{{\rm{\Delta }}}_{so}}{3}\cdot [\begin{array}{cccccc}0 & -i & 0 & 0 & 0 & 1\\ -i & 0 & 0 & 0 & 0 & -i\\ 0 & 0 & 0 & -1 & i & 0\\ 0 & 0 & -1 & 0 & i & 0\\ 0 & 0 & -i & -i & 0 & 0\\ 1 & i & 0 & 0 & 0 & 0\end{array}]$$

where Δ_*so*_ represents the spin-orbit splitting energy.

**Figure 3:**
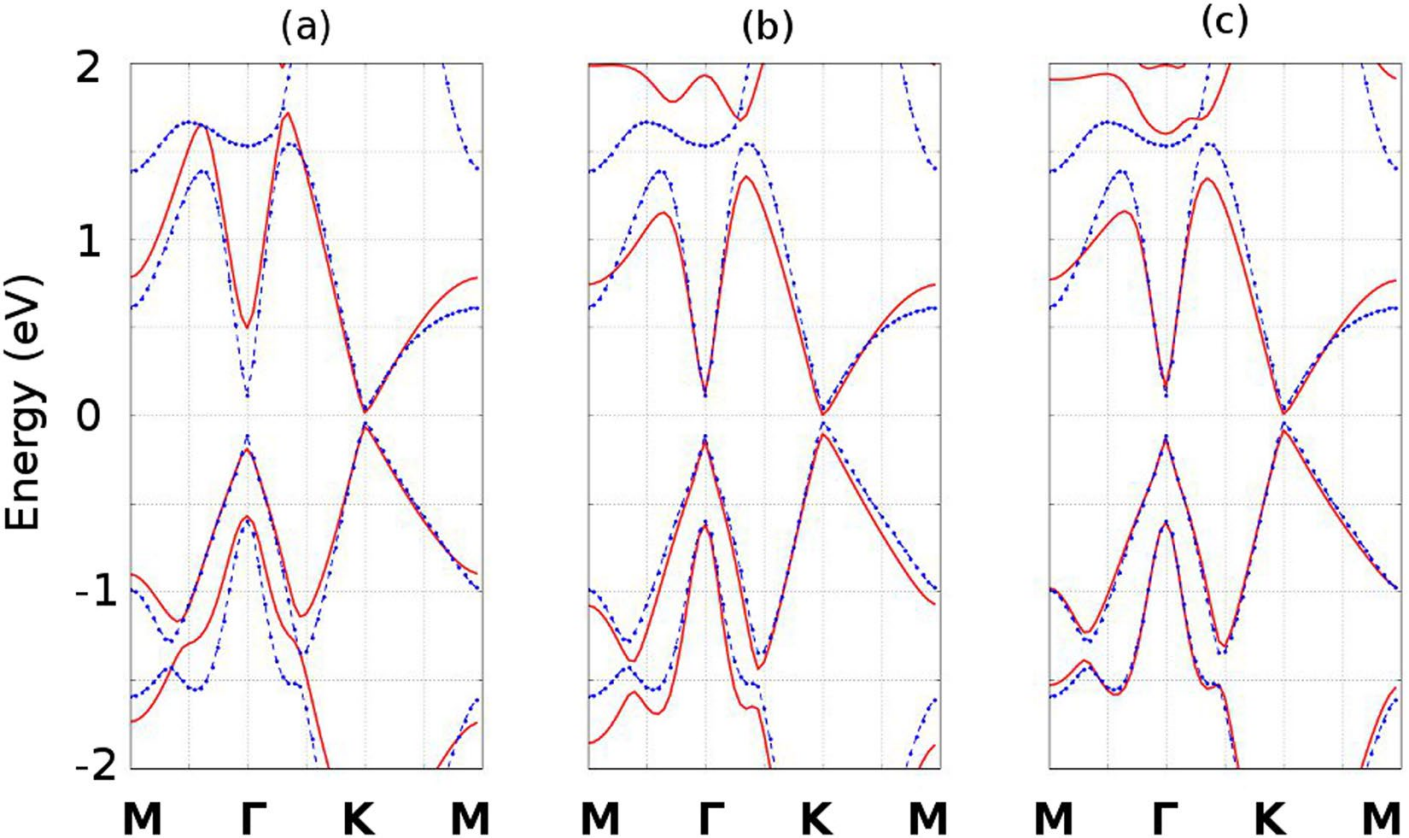
Monolayer stanene bandstructure with the spin orbital coupling effect: comparison between the DFT calculation and our tight binding model. The blue dotted lines represent the bandstructure calculated by means of DFT with spin orbital interaction, whereas the red solid lines represent the bandstructure calculated by means of our tight binding model. (**a**), (**b**) and (**c**) represent the comparisons for the NNTB, 2NTB and 3NTB models, respectively.

Hence, we implemented the spin-orbit coupling interaction for our NNTB, 2NTB and 3NTB model. By comparing to the DFT calculation of the bandstructure of stanene, we fit Δ_*so*_ in order to match the DFT calculated result. We find that the spin-orbit splitting energy Δ_*so*_ ≈ 0.672 eV best matches with the DFT calculation. Our bandstructure with the spin orbital coupling effect is presented in Fig. 3.

### Analytical solution of high symmetry point $\overrightarrow{{\boldsymbol{\Gamma }}}$ and $\overrightarrow{{\boldsymbol{K}}}$

We simplify the 2NTB Hamiltonian for stanene at the $\overrightarrow{{\rm{\Gamma }}}$ and $\overrightarrow{K}$ points and obtain the analytical dispersion relation by evaluating its eigenvalues. The simplified Hamiltonian is shown in Appendix A. The analytically solved eigenvalues are reported below:

9 $${E}_{{p}_{x}\pm }({\rm{\Gamma }})={{\rm{\Gamma }}}_{1}\pm {{\rm{\Gamma }}}_{4}$$

10 $${E}_{{p}_{y}\pm }({\rm{\Gamma }})={{\rm{\Gamma }}}_{1}\pm {{\rm{\Gamma }}}_{4}$$

11 $${E}_{s{p}_{z}\pm }({\rm{\Gamma }})=\frac{{{\rm{\Gamma }}}_{0}+{{\rm{\Gamma }}}_{2}-{{\rm{\Gamma }}}_{3}+{{\rm{\Gamma }}}_{6}\pm \sqrt{{({{\rm{\Gamma }}}_{2}-{{\rm{\Gamma }}}_{0}+{{\rm{\Gamma }}}_{3}+{{\rm{\Gamma }}}_{6})}^{2}+4{{\rm{\Gamma }}}_{5}^{2}}}{2}$$

12 $${E}_{s{p}_{z}\pm }({\rm{\Gamma }}{)}^{^{\prime} }=\frac{{{\rm{\Gamma }}}_{0}+{{\rm{\Gamma }}}_{2}+{{\rm{\Gamma }}}_{3}-{{\rm{\Gamma }}}_{6}\pm \sqrt{{({{\rm{\Gamma }}}_{0}-{{\rm{\Gamma }}}_{2}+{{\rm{\Gamma }}}_{3}+{{\rm{\Gamma }}}_{6})}^{2}+4{{\rm{\Gamma }}}_{5}^{2}}}{2}.$$

The valence band dispersion relation at the $\overrightarrow{{\rm{\Gamma }}}$ point is double degenerate with representation Γ_1_ − Γ_4_ and the conduction band analytical dispersion relation is $\tfrac{{{\rm{\Gamma }}}_{0}+{{\rm{\Gamma }}}_{2}-{{\rm{\Gamma }}}_{3}+{{\rm{\Gamma }}}_{6}+\sqrt{{({{\rm{\Gamma }}}_{2}-{{\rm{\Gamma }}}_{0}+{{\rm{\Gamma }}}_{3}+{{\rm{\Gamma }}}_{6})}^{2}+4{{\rm{\Gamma }}}_{5}^{2}}}{2}$. From the expression, we can draw the conclusion that, at the $\overrightarrow{{\rm{\Gamma }}}$ point, the conduction band mainly consists of *s*, *p*_*z*_ hybridization, whereas the valence band is mainly a hybrid of *p*_*x*_, *p*_*y*_ orbitals.

At the *K* point, we apply the basis transformation:

13 $$|{\psi }_{xy+}^{a}\rangle =\frac{\sqrt{2}}{2}|{p}_{x}^{a}\rangle +\frac{i\sqrt{2}}{2}|{p}_{y}^{a}\rangle $$

14 $$|{\psi }_{xy-}^{a}\rangle =\frac{\sqrt{2}}{2}|{p}_{x}^{a}\rangle -\frac{i\sqrt{2}}{2}|{p}_{y}^{a}\rangle $$

15 $$|{\psi }_{xy+}^{b}\rangle =\frac{\sqrt{2}}{2}|{p}_{x}^{b}\rangle +\frac{i\sqrt{2}}{2}|{p}_{y}^{b}\rangle $$

16 $$|{\psi }_{xy-}^{b}\rangle =\frac{\sqrt{2}}{2}|{p}_{x}^{b}\rangle -\frac{i\sqrt{2}}{2}|{p}_{y}^{b}\rangle $$

We then transfer the original Hamiltonian into the new basis set $\{|{s}^{a}\rangle ,|{\psi }_{xy+}^{b}\rangle ,|{p}_{z}^{a}\rangle ,|{s}^{b}\rangle ,|{\psi }_{xy-}^{a}\rangle ,|{p}_{z}^{b}\rangle ,|{\psi }_{xy-}^{b}\rangle ,|{\psi }_{xy+}^{a}\rangle \}$ and we obtain the Hamiltonian ${\tilde{H}}_{K}$:

17 $$[{\mathop{H}\limits^{ \sim }}_{K}]=[\begin{array}{ccc}{\kappa }_{0} & 0 & 0\\ 0 & {\kappa }_{1} & 0\\ 0 & 0 & {\kappa }_{2}\end{array}]$$

where *κ*_0_, *κ*_1_ is a 3 × 3 matrix and *κ*_2_ is a 2 × 2 matrix. We can obtain the analytical expression of the energy bands by diagonalizing [*H*_*K*_]. The details are included in Appendix A.

### Buckling effect on stanene bandstructure

Due to the large inter atomic distance, monolayer stanene exhibits a mixture of *sp* ^2^ and *sp* ^3^ orbital hybridization in order to stabilize the structure, which naturally leads to a buckled hexagonal lattice^18,19^. The buckling distance can influence the electronic properties of the material; therefore, this can be engineered to achieve further tunability of the material. For example, many methods including chemical decoration^20,21^, substrate interaction^22^, mechanical strain^23^ can be applied to buckled materials, in order to tune their properties.

**Figure 4:**
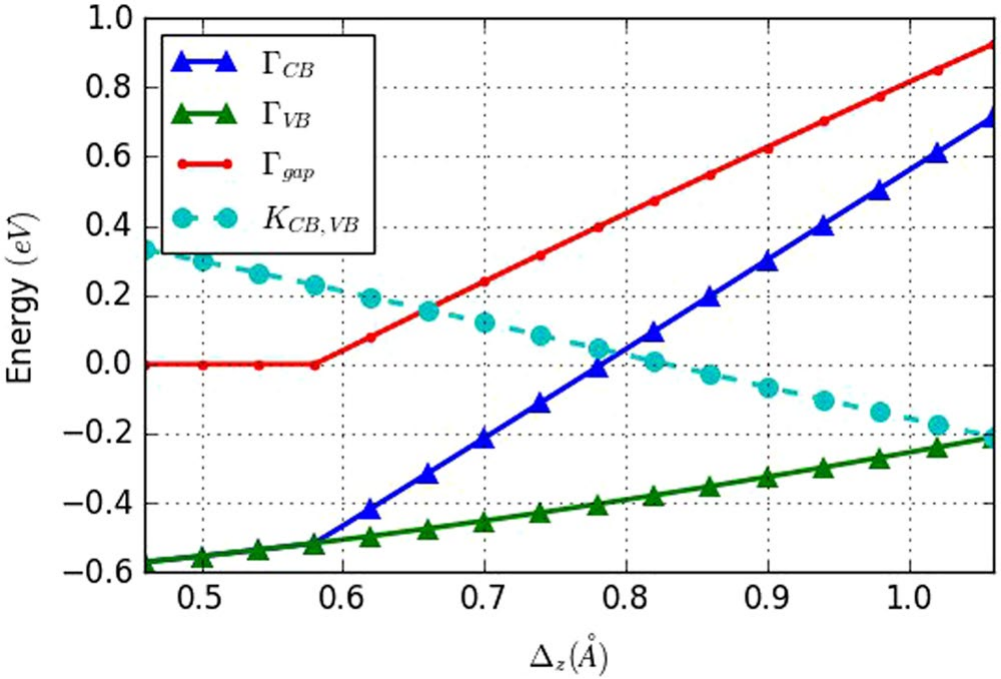
Band energy variation as a function of the buckling distance, without spin-orbital interaction. Γ_*CB*_ and Γ_*VB*_ represent the conduction band and valence band energy at the $\overrightarrow{{\rm{\Gamma }}}$ point. Γ_*gap*_ is the energy difference between the conduction and valence bands (Γ_*CB*_ − Γ_*VB*_). *K*_*CB*,*VB*_ represents the degenerate energy of the conduction and valence bands in the $\overrightarrow{K}$ point. All the energies are relative to the Fermi level.

**Figure 5:**
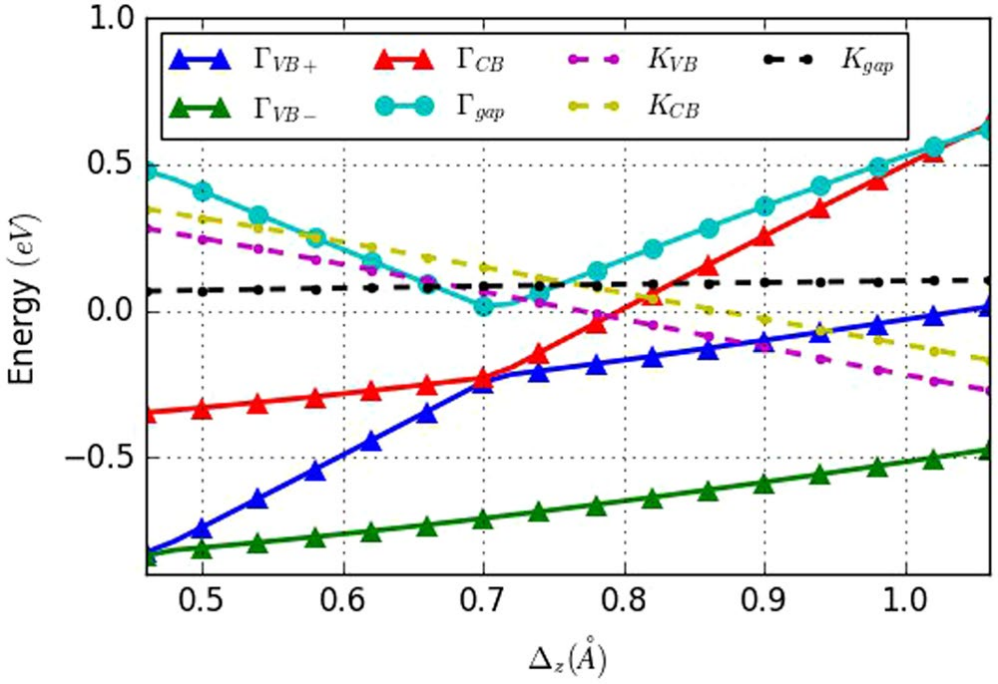
Band energy variation as a function of the buckling distance, with spin-orbital interaction. Γ_*VB*+_ and Γ_*VB*−_ represent the energies of the valence bands with higher and lower energy at the $\overrightarrow{{\rm{\Gamma }}}$ point, which are separated due to the spin-orbital coupling. Γ_*CB*_ represents the conductance band energy. Γ_*gap*_ is the energy difference between the conduction and the higher valence band (Γ_*CB*_ − Γ_*VB*+_). *K*_*CB*_ and *K*_*VB*_ represent the energies of the conduction and valence band in the $\overrightarrow{K}$ point. *K*_*gap*_ is the energy difference between *K*_*CB*_ and *K*_*VB*_. All the energies are relative to the Fermi level.

In this paper, we conducted a simulation study on the relationship between the buckling distance and the stanene energy bands using our tight binding model. In order to evaluate the relationship between buckling distance and energy bands, we keep our TB parameters the same while varying the buckling distance Δ_*z*_ in the tight binding model and then calculate the stanene band structure for each buckling distance. We find that the buckling distance Δ_*z*_ determines the energy gap between conduction and valence bands. Decreasing the bulking will result into an energy gap reduction, whereas increasing the bulking distance can increase the energy gap at the $\overrightarrow{{\rm{\Gamma }}}$ point. When Δ_*z*_ is smaller than 0.58 Å, the conduction and valence bands overlap. The buckling can also change the energies of the conduction and valence bands at the $\overrightarrow{K}$ point, where reducing the buckling can lift the band energies and increasing the buckling can reduce the band energies. Furthermore, our calculation indicates that for Δ_*z*_ larger than 0.58 Å, the energies changes linearly respect to the buckling distance, as shown in Fig. 4. We also study the buckling impact when we include the spin orbital interaction. The result is presented in Fig. 5. As far as the variation of the bands in the $\overrightarrow{K}$ point is concerned, we notice that the buckling distance does not affect the energy gap of the $\overrightarrow{K}$ point, however, it reduces the band energy when Δ_*z*_ increases. On the other hand, the energy varies differently at the $\overrightarrow{{\rm{\Gamma }}}$ point. For Δ_*z*_ larger than 0.7 Å, the energy difference between Γ_*VB*+_ and Γ_*VB*−_ remains the same and increasing Δ_*z*_ tends to increase both the conduction and valence band energies (Γ_*CB*_, Γ_*VB*+_, Γ_*VB*−_), as well as the energy difference between the conduction and valence bands (Γ_*gap*_ = Γ_*CB*_ − Γ_*VB*+_). At 0.7 Å, the conduction band and valence bands become the same, so that the energy gap is zero. However, when Δ_*z*_ is less than 0.7 Å, further reducing Δ_*z*_ can increase the Γ_*gap*_. In addition, the energy difference between Γ_*VB*+_ and Γ_*VB*−_ starts to decrease, whereas the energy difference between Γ_*CB*_ and Γ_*VB*−_ becomes almost constant.

## $\overrightarrow{{\boldsymbol{k}}}\cdot \overrightarrow{{\boldsymbol{p}}}$ approximation

As we have previously stated, a tight-binding analysis requires much less computational resources and time than a DFT calculation. However, its requirements increase with the number of atoms and can make it inapplicable in cases in which large structures have to be simulated. In these cases a $\overrightarrow{k}\cdot \overrightarrow{p}$ approach^24^ (analogous to that already used by some of us for the simulation of large graphene devices^25–29^) can be preferable (see also refs^30–34^). Therefore, here we have also obtained the $\overrightarrow{k}\cdot \overrightarrow{p}$ dispersion relations of stanene near the points $\overrightarrow{K}^{\prime}$, $\overrightarrow{K}$, and $\overrightarrow{{\rm{\Gamma }}}$, where the highest valence band and the lowest conduction band have local maxima and minima, respectively (see the DFT and tight binding results shown in Figs 2 and 3). As we explain in detail in the Appendix B, around each of these 3 points of the reciprocal space, starting from our nearest-neighbor tight-binding relations with the parameter Δ_*pz*_, we have performed a low-order Taylor expansion of the tight-binding Hamiltonian and then, in order to reduce its size, we have projected it onto the states corresponding to the dispersion relations nearest to the Fermi energy. In particular, around $\overrightarrow{K}^{\prime}$ and $\overrightarrow{K}$, noting the approximately linear behavior of the DFT and tight-binding bands, we have performed only a first-order Taylor expansion and then we have projected the Hamiltonian onto 2 states (4 including the spin). The $\overrightarrow{k}\cdot \overrightarrow{p}$ Hamiltonians we have obtained around $\overrightarrow{K}^{\prime}$ are in the absence of spin-orbit interaction:

18 $${H}_{\overrightarrow{{K}^{^{\prime} }}}(\overrightarrow{\kappa })=[\begin{array}{cc}{\varepsilon }_{1} & \gamma ({\kappa }_{x}+i{\kappa }_{y})\\ \gamma ({\kappa }_{x}-i{\kappa }_{y}) & {\varepsilon }_{1}\end{array}]$$

and in the presence of spin-orbit interaction:

19 $${H}_{\overrightarrow{{K}^{{\rm{^{\prime} }}}}}(\overrightarrow{\kappa })\otimes {I}_{2}+[\begin{array}{cccc}{d}_{1} & 0 & 0 & 0\\ 0 & {d}_{2} & 0 & 0\\ 0 & 0 & {d}_{2} & 0\\ 0 & 0 & 0 & {d}_{1}\end{array}]$$

(*I*_2_ is the 2 × 2 identity matrix). Around $\overrightarrow{K}$, we have obtained in the absence of spin-orbit interaction:

20 $${H}_{\overrightarrow{K}}(\overrightarrow{\kappa })=[\begin{array}{cc}{\varepsilon }_{1} & -\gamma ({\kappa }_{x}-i{\kappa }_{y})\\ -\gamma ({\kappa }_{x}+i{\kappa }_{y}) & {\varepsilon }_{1}\end{array}]$$

and in the presence of spin-orbit interaction:

21 $${H}_{\overrightarrow{K}}(\overrightarrow{\kappa })\otimes {I}_{2}+[\begin{array}{cccc}{d}_{2} & 0 & 0 & 0\\ 0 & {d}_{1} & 0 & 0\\ 0 & 0 & {d}_{1} & 0\\ 0 & 0 & 0 & {d}_{2}\end{array}]$$

Instead, around $\overrightarrow{{\rm{\Gamma }}}$, where the dispersion relations are not linear any more, we have performed a second-order Taylor expansion and then we have projected the Hamiltonian onto 3 states (6 including the spin), obtaining in the absence of spin-orbit interaction the following $\overrightarrow{k}\cdot \overrightarrow{p}$ Hamiltonian:

22 $${H}_{\overrightarrow{{\rm{\Gamma }}}}(\overrightarrow{\kappa })=[\begin{array}{ccc}{\varepsilon }_{3}^{{\rm{^{\prime} }}}+{c}_{3}({\kappa }_{x}^{2}+{\kappa }_{y}^{2}) & -i{c}_{4}{\kappa }_{x} & -i{c}_{4}{\kappa }_{y}\\ i{c}_{4}{\kappa }_{x} & -{V}_{6}+{c}_{5}{\kappa }_{x}^{2}+{c}_{6}{\kappa }_{y}^{2} & -{c}_{7}{\kappa }_{x}{\kappa }_{y}\\ i{c}_{4}{\kappa }_{y} & -{c}_{7}{\kappa }_{x}{\kappa }_{y} & -{V}_{6}+{c}_{6}{\kappa }_{x}^{2}+{c}_{5}{\kappa }_{y}^{2}\end{array}]$$

and in the presence of spin-orbit interaction:

23 $${H}_{\overrightarrow{{\rm{\Gamma }}}}(\overrightarrow{\kappa })\otimes {I}_{2}+[\begin{array}{cccccc}{f}_{1} & 0 & 0 & 0 & 0 & 0\\ 0 & {f}_{1} & 0 & 0 & 0 & 0\\ 0 & 0 & {f}_{2} & 0 & -i{f}_{3} & 0\\ 0 & 0 & 0 & {f}_{2} & 0 & i{f}_{3}\\ 0 & 0 & i{f}_{3} & 0 & {f}_{2} & 0\\ 0 & 0 & 0 & -i{f}_{3} & 0 & {f}_{2}\end{array}]$$

**Figure 6:**
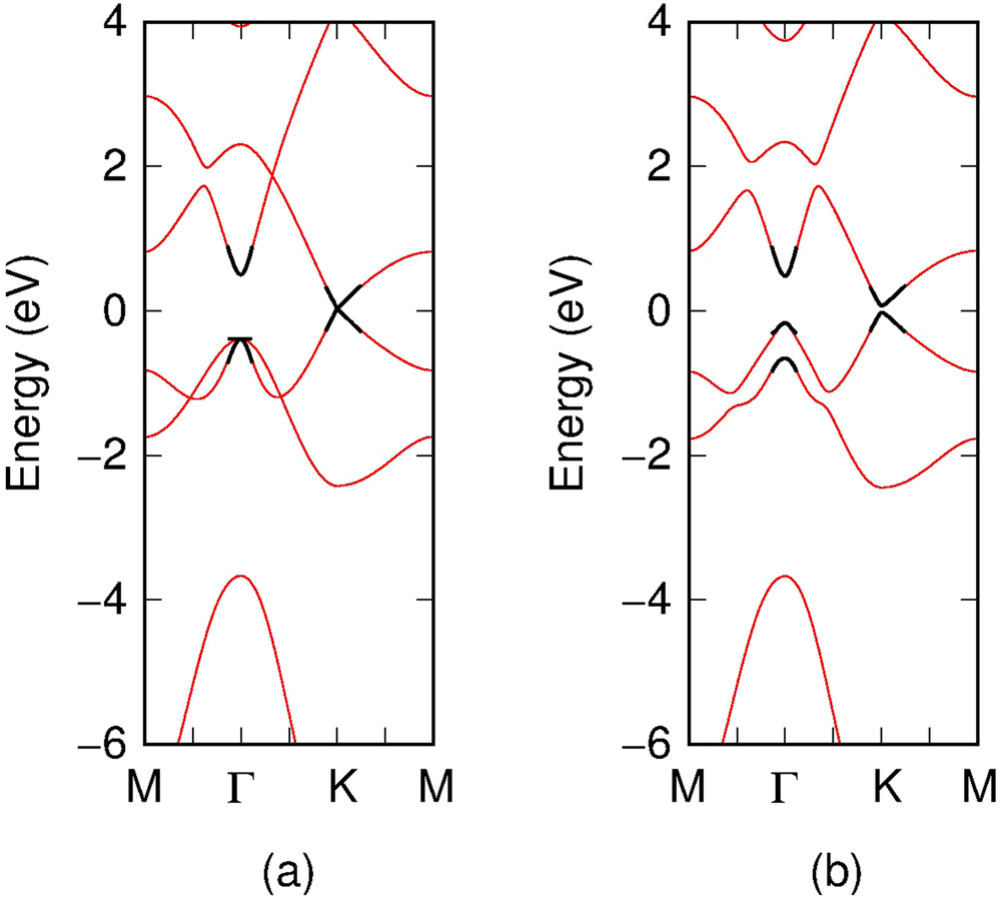
Dispersion relations of stanene, obtained neglecting (**a**) and considering (**b**) spin-orbit interaction. With the thin red line we show the nearest-neighbor tight-binding bands, while with the thick black line we report the $\overrightarrow{k}\cdot \overrightarrow{p}$ dispersion relations obtained with the parameters of Table 2.

In the Appendix B we derive these expressions and we write the relations between the $\overrightarrow{k}\cdot \overrightarrow{p}$ parameters which appear in these matrices and the nearest-neighbor tight-binding parameters. In Table 2 we report the values of the $\overrightarrow{k}\cdot \overrightarrow{p}$ parameters obtained from the NNTB parameters of Table 1 using these relations and in Fig. 6 we compare the NNTB bands with the $\overrightarrow{k}\cdot \overrightarrow{p}$ dispersion relations of stanene, computed adopting these values for the $\overrightarrow{k}\cdot \overrightarrow{p}$ parameters.

**Table 2:** Values of the $\overrightarrow{k}\cdot \overrightarrow{p}$ parameters obtained from the nearest-neighbor tight-binding parameters.

$\overrightarrow{{\boldsymbol{k}}}\cdot \overrightarrow{{\boldsymbol{p}}}$ parameter	value obtained from NNTB parameters
*ε*_1_	3.1749 · 10^−2^ eV
*γ*	2.9001 · 10^−1^ eV · nm
*ε*_3′_	5.0316 · 10^−1^ eV
*V*_6_	3.9049 · 10^−1^ eV
*c*_3_	−2.3078 · 10^−2^ eV · nm^2^
*c*_4_	6.6487 · 10^−1^ eV · nm
*c*_5_	7.8477 · 10^−2^ eV · nm^2^
*c*_6_	1.2379 · 10^−3^ eV · nm^2^
*c*_7_	−7.7239 · 10^−2^ eV · nm^2^
*d*_1_	−5.3937 · 10^−2^ eV
*d*_2_	4.2190 · 10^−2^ eV
*f*_1_	−1.9452 · 10^−2^ eV
*f*_2_	−2.0429 · 10^−2^ eV
*f*_3_	2.4443 · 10^−1^ eV

A $\overrightarrow{k}\cdot \overrightarrow{p}$ description with 4 bands near $\overrightarrow{K}$ and 6 bands near $\overrightarrow{{\rm{\Gamma }}}$ is considered.

**Figure 7:**
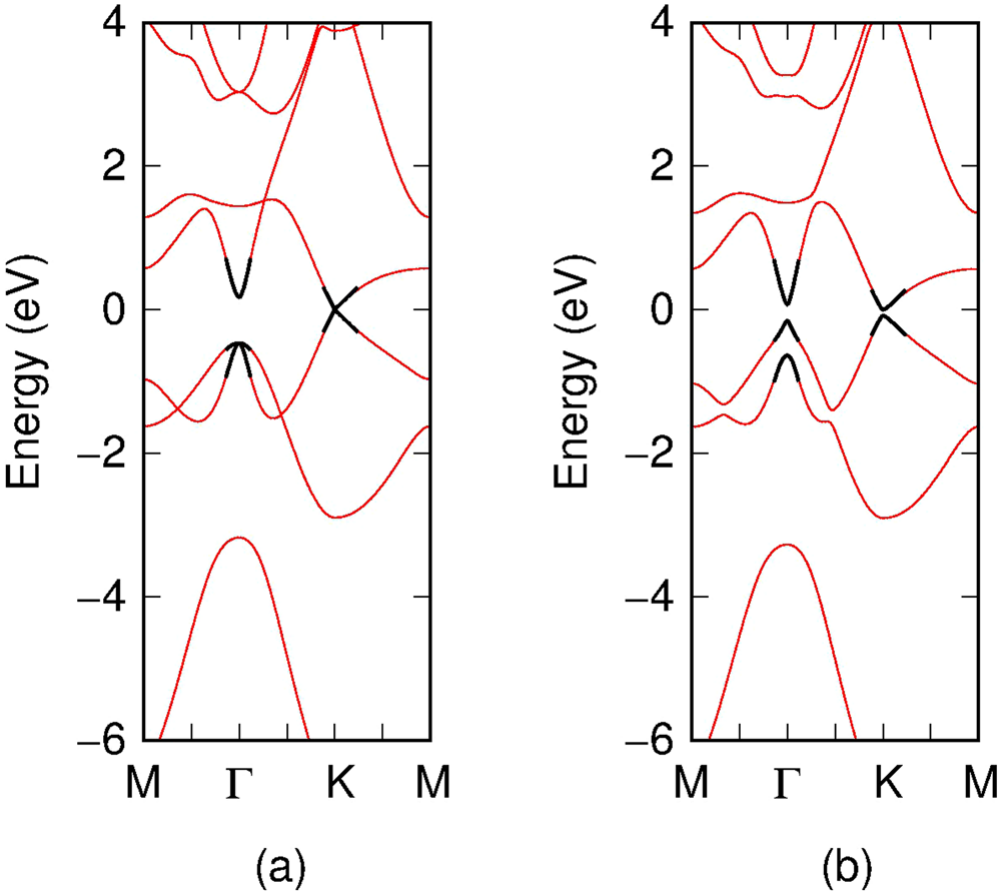
Dispersion relations of stanene, obtained neglecting (**a**) and considering (**b**) spin-orbit interaction. With the thin red line we show the DFT bands, while with the thick black line we report the $\overrightarrow{k}\cdot \overrightarrow{p}$ dispersion relations obtained with the parameters of Table 3.

Then, starting from these expressions for the $\overrightarrow{k}\cdot \overrightarrow{p}$ Hamiltonians, we have instead used the $\overrightarrow{k}\cdot \overrightarrow{p}$ parameters as fitting parameters to better reproduce the DFT dispersion relations around the points $\overrightarrow{K}^{\prime}$, $\overrightarrow{K}$, and $\overrightarrow{{\rm{\Gamma }}}$ (using the minimization algorithm proposed in ref.^35^). The fitting procedure is described in the Appendix B; the resulting values of the $\overrightarrow{k}\cdot \overrightarrow{p}$ parameters are reported in Table 3. In Fig. 7 we compare the DFT bands with the $\overrightarrow{k}\cdot \overrightarrow{p}$ dispersion relations of stanene, computed substituting these values to the $\overrightarrow{k}\cdot \overrightarrow{p}$ parameters.

**Table 3:** Values of the $\overrightarrow{k}\cdot \overrightarrow{p}$ parameters obtained fitting the DFT dispersion relations.

$\overrightarrow{{\boldsymbol{k}}}\cdot \overrightarrow{{\boldsymbol{p}}}$ parameter	value fitted from DFT
*ε*_1_	−2.7173 · 10^−4^ eV
*γ*	2.9360 · 10^−1^ eV · nm
*ε*_3′_	1.7506 · 10^−1^ eV
*V*_6_	4.6869 · 10^−1^ eV
*c*_3_	3.5993 · 10^−3^ eV · nm^2^
*c*_4_	7.2639 · 10^−1^ eV · nm
*c*_5_	5.5394 · 10^−2^ eV · nm^2^
*c*_6_	−8.3891 · 10^−2^ eV · nm^2^
*c*_7_	−1.3307 · 10^−1^ eV · nm^2^
*d*_1_	−3.8364 · 10^−2^ eV
*d*_2_	4.6786 · 10^−2^ eV
*f*_1_	−5.5095 · 10^−2^ eV
*f*_2_	1.1570 · 10^−1^ eV
*f*_3_	2.4084 · 10^−1^ eV

A $\overrightarrow{k}\cdot \overrightarrow{p}$ description with 4 bands near $\overrightarrow{K}$ and 6 bands near $\overrightarrow{{\rm{\Gamma }}}$ is considered.

## TB results with lateral confinement and magnetic field

### Stanene nanoribbons

In this section, we investigate the properties of both armchair and zigzag stanene nanoribbons using our 3NTB parameters, both with and without spin orbital coupling. In this step, we implemented our model parameter into the Kwant Package^36^, which is a python-based tight-binding package that allows users to customize codes and to perform quantum transport calculations.

**Figure 8:**
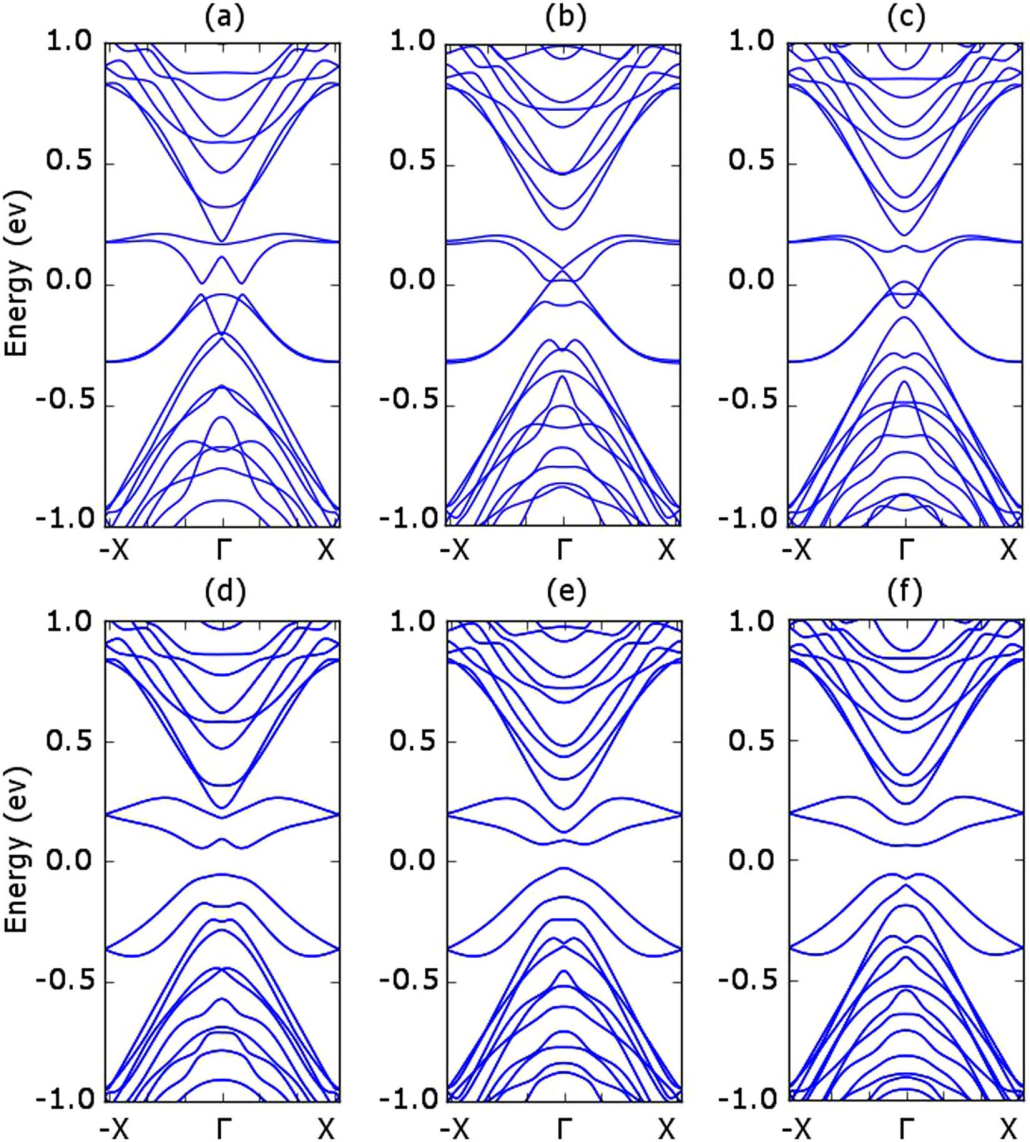
Armchair stanene nanoribbon bandstructure with 12, 13, 14 armchair lines. The panels (**a**–**c**) report the results without spin orbital coupling, whereas the panels (**d**–**f**) report the results with the spin orbital coupling effect.

**Figure 9:**
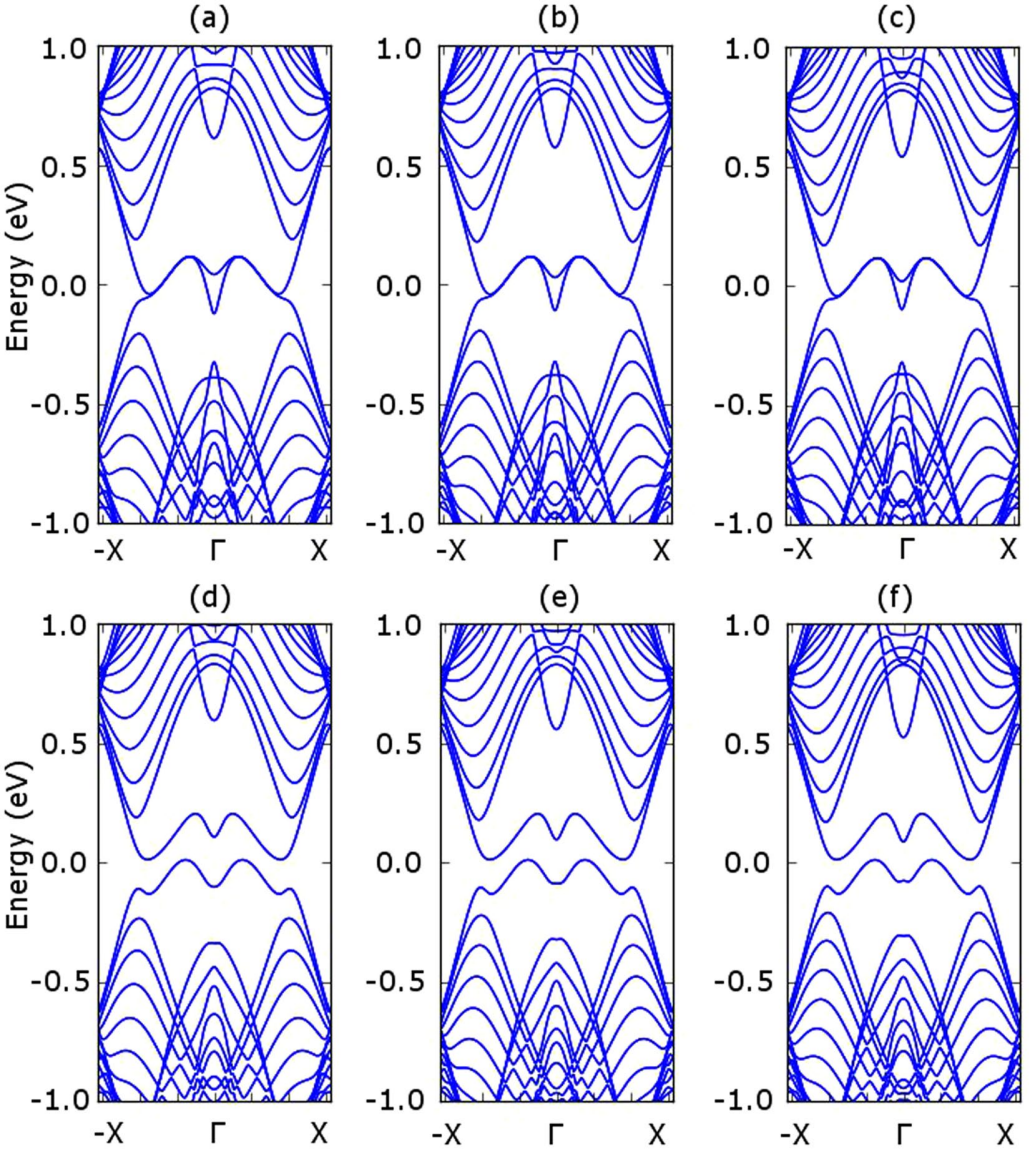
Zigzag stanene nanoribbon bandstructure with 12, 13, 14 zigzag lines. The panels (**a–c**) report the results without spin orbital coupling, whereas the panels (**d–f**) report the results with the spin orbital coupling effect.

We first obtain the band structure for armchair stanene nanoribbons (ASNR). As shown in Fig. 8, if we do not consider the spin orbital interaction, the armchair stanene nanoribbons show different characteristics for the 12, 13 and 14 ASNRs (i.e. for the armchair stanene nanoribbons with 12, 13, 14 armchair lines). However, the spin orbital interaction opens up band gaps for all the three narrow ASNRs. Similarly, the spin orbital interaction can also open up a band gap for narrow zigzag stanene nano ribbons (ZSNRs), as shown in Fig. 9.

**Figure 10:**
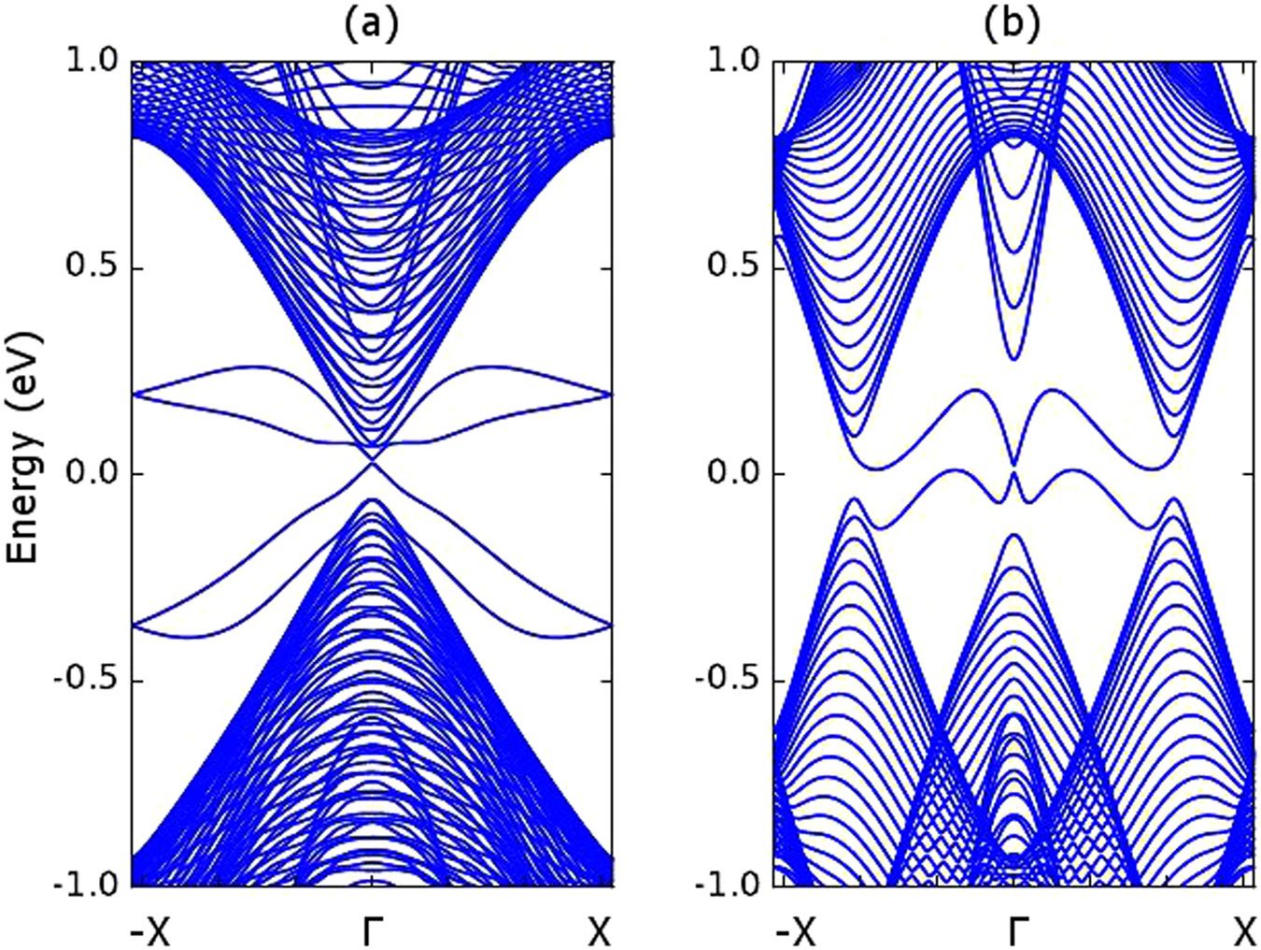
Bandstructure of wide stanene nanoribbons with the spin orbital interaction effect. (**a**) Armchair stanene nanoribbon with 15.2 nm width. (**b**) Zigzag stanene nanoribbon with 15.2 nm width.

We calculate the bandstructure of an ASNR with a width of 15.2 nm to compare it with the previous literature^5^. As shown in Fig. 10, there is a clear Dirac corn which matches with the previous DFT based calculations. We further study the bandstructure of a zigzag stanene nanoribbon with 15.2 nm width and we notice there exists a narrow energy gap around the $\overrightarrow{{\rm{\Gamma }}}$ point. This gap reduces as the nanoribbon width increases and eventually becomes negligibly small when the ribbon becomes wider than 20 nm.

### Magnetic field effect on stanene nanoribbon

We also investigated the properties of stanene nanoribbons in the presence of an external homogeneous vertical magnetic field by adopting the Peierls substitution. The Peierls substitution takes the magnetic field into account by multiplying the hopping integral by a phase component. A vertical magnetic field can be represented by a vector $\overrightarrow{B}=\mathrm{(0,}\,0,\,{B}_{z})$. The corresponding magnetic vector potential is related to the magnetic field by the relationship $\overrightarrow{B}=\overrightarrow{\nabla }\times \overrightarrow{A}$: for example, we take $\overrightarrow{A}=(-{B}_{z}y,\,\mathrm{0,}\,\mathrm{0)}$. By applying the Peierls substitution, we can calculate the hopping integral with the effect of magnetic field [*H*]_*P*_ multiplying the hopping integral matrix ${[H]}_{\alpha }^{\beta }$ from the stanene site *α* to the site *β* without magnetic field by a phase term due to the magnetic field:

24 $${[H]}_{P}={[H]}_{\alpha }^{\beta }\cdot {e}^{i{\varphi }_{\alpha ,\beta }}$$

25 $${\varphi }_{\alpha ,\beta }=\frac{e}{\hslash }{\int }_{\alpha }^{\beta }\,\overrightarrow{A}\cdot d\overrightarrow{r}.$$

**Figure 11:**
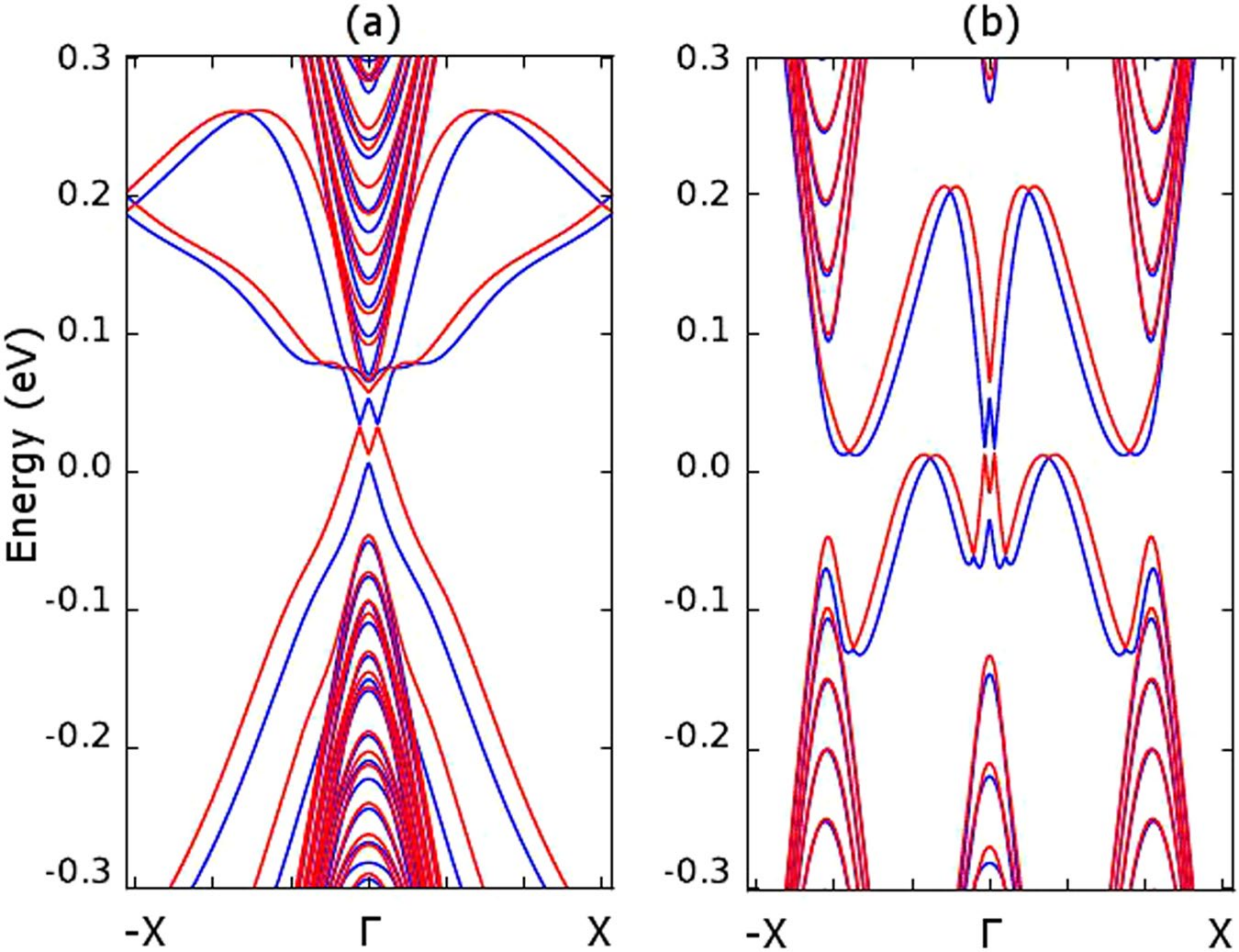
Bandstructure of stanene nanoribbons with the spin orbital interaction effect under a 1 Tesla magnetic field. The red and blue lines indicate the different spin components. (**a**) 15.2 nm wide armchair stanene nanoribbon. (**b**) 15.2 nm wide zigzag stanene nanoribbon.

By incorporating the magnetic field into our model, we calculated the stanene nanoribbon bandstructure under a 1 Tesla magnetic field. As shown in Fig. 11, the magnetic field can split the bandstructure of spin up and spin down electrons and make the stanene nanoribbon useful for spintronics applications.

## Conclusion

We have developed efficient approaches to the determination of the bandstructure of stanene and of stanene nanoribbons, which can be useful for large scale simulations. In particular, we focused on a tight-binding model and on a $\overrightarrow{k}\cdot \overrightarrow{p}$ description in the $\overrightarrow{K}$, $\overrightarrow{K}^{\prime}$ and $\overrightarrow{{\rm{\Gamma }}}$ points of the reciprocal space. The on-site and hopping energy parameters for the adopted tight-binding *sp* ^3^ model have been obtained fitting DFT calculations. Nearest-neighbor, second nearest neighbor and third nearest neighbor models have been considered, achieving a particularly good agreement with the DFT reference, especially in the high-symmetry points, which makes our approach suitable for the investigation of the low-energy properties of stanene. Our tight-binding model has been applied also to the investigation of the bandstructure of armchair and zigzag nanoribbons, with the inclusion of the spin-obit interaction and of the effect of an orthogonal magnetic field. A further reduction of the computational burden can be achieved with the $\overrightarrow{k}\cdot \overrightarrow{p}$ approach, that we have applied around the high-symmetry points, obtaining the values of the parameters that appear in the Hamiltonian by fitting the results to the tight-binding and DFT bands. We have also investigated the effect of buckling in the stanene lattice on the band structure, in particular on the energy gap.

## Electronic supplementary material

Appendices

SUPPLEMENTARY MATERIAL
