# Supplementary material for: A tight binding and k → ⋅ p → study of monolayer stanene
Source: Sci Rep. 2017 Sep 21;7:12069. doi: 10.1038/s41598-017-12281-y (PMC5608720; doi:10.1038/s41598-017-12281-y)
Supplement: Supplementary file 1 — Appendices [file 41598_2017_12281_MOESM1_ESM.pdf]

APPENDICES

for the paper:

Liming Jiang, Paolo Marconcini, Md Sharafat Hossian,
Wanzhi Qiu, Robin Evans, Massimo Macucci, Efstratios Skafidas,

“A TIGHT BINDING AND $\vec{k} \cdot \vec{p}$ STUDY OF MONOLAYER STANENE”

APPENDIX A: TIGHT BINDING HAMILTONIAN ELEMENTS

The 2NTB Hamiltonian for stanene at the $\vec{\Gamma}$ point can be simplified as:

$$[\Gamma] = \begin{bmatrix} \Gamma_0 & 0 & 0 & 0 & \Gamma_3 & 0 & 0 & -\Gamma_5 \\ 0 & \Gamma_1 & 0 & 0 & 0 & \Gamma_4 & 0 & 0 \\ 0 & 0 & \Gamma_1 & 0 & 0 & 0 & \Gamma_4 & 0 \\ 0 & 0 & 0 & \Gamma_2 & \Gamma_5 & 0 & 0 & \Gamma_6 \\ \Gamma_3 & 0 & 0 & \Gamma_5 & \Gamma_0 & 0 & 0 & 0 \\ 0 & \Gamma_4 & 0 & 0 & 0 & \Gamma_1 & 0 & 0 \\ 0 & 0 & \Gamma_4 & 0 & 0 & 0 & \Gamma_1 & 0 \\ -\Gamma_5 & 0 & 0 & \Gamma_6 & 0 & 0 & 0 & \Gamma_2 \end{bmatrix} \tag{S1}$$

Its eigenvalues can be calculated by applying a unitary transformation:

$$\tilde{\Gamma} = U_0^\dagger \Gamma U_0 \tag{S2}$$

where:

$$U_0 = \begin{bmatrix} 1 & 0 & 0 & 0 & 0 & 0 & 0 & 0 \\ 0 & 0 & 0 & 0 & 1 & 0 & 0 & 0 \\ 0 & 0 & 0 & 0 & 0 & 0 & 0 & 1 \\ 0 & 0 & 0 & 1 & 0 & 0 & 0 & 0 \\ 0 & 1 & 0 & 0 & 0 & 0 & 0 & 0 \\ 0 & 0 & 0 & 0 & 0 & 1 & 0 & 0 \\ 0 & 0 & 0 & 0 & 0 & 0 & 1 & 0 \\ 0 & 0 & 1 & 0 & 0 & 0 & 0 & 0 \end{bmatrix} \tag{S3}$$

The resulting transformed Hamiltonian $\tilde{\Gamma}$ can be represented as:

$$\tilde{\Gamma} = \begin{bmatrix} \gamma_0 & 0 & 0 \\ 0 & \gamma_1 & 0 \\ 0 & 0 & \gamma_1 \end{bmatrix} \tag{S4}$$

where:

$$\gamma_0 = \begin{bmatrix} \Gamma_0 & \Gamma_3 & -\Gamma_5 & 0 \\ \Gamma_3 & \Gamma_0 & 0 & \Gamma_5 \\ -\Gamma_5 & 0 & \Gamma_2 & \Gamma_6 \\ 0 & \Gamma_5 & \Gamma_6 & \Gamma_2 \end{bmatrix} \tag{S5}$$

and

$$\gamma_1 = \begin{bmatrix} \Gamma_1 & \Gamma_4 \\ \Gamma_4 & \Gamma_1 \end{bmatrix} \tag{S6}$$

with:

$$\Gamma_0 = \epsilon_s + 6ss\sigma_2 \tag{S7}$$

$$\Gamma_1 = \epsilon_p + 3pp\pi_2 + 3pp\sigma_2 \tag{S8}$$

$$\Gamma_2 = \epsilon_p + 6pp\pi_2 \tag{S9}$$

$$\Gamma_3 = 3ss\sigma \tag{S10}$$

$$\Gamma_4 = 3pp\pi - \frac{a_0^2}{2b^2}pp\pi + \frac{a_0^2}{2b^2}pp\sigma \tag{S11}$$

$$\Gamma_5 = \frac{3\Delta_z}{b}sp\sigma \tag{S12}$$

$$\Gamma_6 = 3pp\pi(1 - \frac{\Delta_z^2}{b^2}) + 3pp\sigma\frac{\Delta_z^2}{b^2} \tag{S13}$$

with $b = \sqrt{(a_0^2/3) + \Delta_z^2}$.

The Hamiltonian H_K for the high symmetry $\vec{K}$ point can be expressed in the basis set $\{s^a, p_x^a, p_y^a, p_z^a, s^b, p_x^b, p_y^b, p_z^b\}$ as:

$$[H_K] = \begin{bmatrix} K_0 & 0 & 0 & 0 & 0 & K_3 & -iK_3 & 0 \\ 0 & K_1 & 0 & 0 & -K_3 & K_4 & iK_4 & -K_5 \\ 0 & 0 & K_1 & 0 & iK_3 & iK_4 & -K_4 & iK_5 \\ 0 & 0 & 0 & K_2 & 0 & -K_5 & iK_5 & 0 \\ 0 & -K_3 & -iK_3 & 0 & K_0 & 0 & 0 & 0 \\ K_3 & K_4 & -iK_4 & -K_5 & 0 & K_1 & 0 & 0 \\ iK_3 & -iK_4 & -K_4 & -iK_5 & 0 & 0 & K_1 & 0 \\ 0 & -K_5 & -iK_5 & 0 & 0 & 0 & 0 & K_2 \end{bmatrix} \tag{S14}$$

This Hamiltonian can be transferred to another basis set by performing the unitary transformation

$$\tilde{H_K} = U_1^\dagger H_K U_1 \tag{S15}$$

where

$$U_1 = \begin{bmatrix} 1 & 0 & 0 & 0 & 0 & 0 & 0 & 0 \\ 0 & 0 & 0 & 0 & \frac{\sqrt{2}}{2} & 0 & 0 & \frac{\sqrt{2}}{2} \\ 0 & 0 & 0 & 0 & -\frac{i\sqrt{2}}{2} & 0 & 0 & \frac{i\sqrt{2}}{2} \\ 0 & 0 & 1 & 0 & 0 & 0 & 0 & 0 \\ 0 & 0 & 0 & 1 & 0 & 0 & 0 & 0 \\ 0 & \frac{\sqrt{2}}{2} & 0 & 0 & 0 & 0 & \frac{\sqrt{2}}{2} & 0 \\ 0 & \frac{i\sqrt{2}}{2} & 0 & 0 & 0 & 0 & -\frac{i\sqrt{2}}{2} & 0 \\ 0 & 0 & 0 & 0 & 0 & 1 & 0 & 0 \end{bmatrix} \tag{S16}$$

The transformed matrix $\tilde{H_K}$ is:

$$\tilde{H_K} = \begin{bmatrix} \kappa_0 & 0 & 0 \\ 0 & \kappa_1 & 0 \\ 0 & 0 & \kappa_2 \end{bmatrix} \tag{S17}$$

with:

$$\kappa_0 = \begin{bmatrix} K_0 & \sqrt{2}K_3 & 0 \\ \sqrt{2}K_3 & K_1 & -\sqrt{2}K_5 \\ 0 & -\sqrt{2}K_5 & K_2 \end{bmatrix} \tag{S18}$$

$$\kappa_1 = \begin{bmatrix} K_0 & -\sqrt{2}K_3 & 0 \\ -\sqrt{2}K_3 & K_1 & -\sqrt{2}K_5 \\ 0 & -\sqrt{2}K_5 & K_2 \end{bmatrix} \tag{S19}$$

$$\kappa_2 = \begin{bmatrix} K_1 & 2K_4 \\ 2K_4 & K_1 \end{bmatrix} \tag{S20}$$

The analytical eigenvalues can be obtained evaluating the determinant of the matrices $\kappa_0, \kappa_1, \kappa_2$. The quadratic expressions for each matrix can be written as:

$$det(\kappa_0 - \lambda I) = (K_0 - \lambda)(K_1 - \lambda)(K_2 - \lambda) - 2K_5^2(K_0 - \lambda) - 2K_3^2(K_2 - \lambda) = 0 \tag{S21}$$

$$det(\kappa_1 - \lambda I) = (K_0 - \lambda)(K_1 - \lambda)(K_2 - \lambda) - 2K_5^2(K_0 - \lambda) - 2K_3^2(K_2 - \lambda) = 0 \tag{S22}$$

$$det(\kappa_2 - \lambda I) = (K_1 - \lambda)^2 - 4K_4^2 = 0 \tag{S23}$$

The matrix elements at the $\vec{K}$ point can be expressed as:

$$K_0 = \epsilon_s - 6ss\sigma_2 \tag{S24}$$

$$K_1 = \epsilon_p - \frac{3}{2}(pp\pi_2 + pp\sigma_2) \tag{S25}$$

$$K_2 = \epsilon_p - 3pp\pi_2 \tag{S26}$$

$$K_3 = \frac{\sqrt{3}a_0}{2b} sp\sigma \tag{S27}$$

$$K_4 = \frac{a_0^2}{4b^2}(pp\sigma - pp\pi) \tag{S28}$$

$$K_5 = \frac{\sqrt{3}\Delta_z a_0}{2b^2}(pp\sigma - pp\pi) \tag{S29}$$

After simplification, we get the polynomial equations:

$$\lambda^3 + \eta_2\lambda^2 + \eta_1\lambda + \eta_0 = 0 \tag{S30}$$

$$(\lambda - K_1)^2 - 4K_4^2 = 0 \tag{S31}$$

where:

$$\eta_0 = 2K_2K_3^2 + 2K_0K_5^2 - K_0K_1K_2 \tag{S32}$$

$$\eta_1 = K_0K_1 + K_1K_2 + K_0K_2 - 2K_4^2 - 2K_5^2 \tag{S33}$$

$$\eta_2 = -(K_0 + K_1 + K_2) \tag{S34}$$

$$\xi_0 = (3\eta_1 - \eta_2^2)/9 \tag{S35}$$

$$\xi_1 = (9\eta_1\eta_2 - 27\eta_0 - 2\eta_2^3)/54 \tag{S36}$$

$$\xi_2 = \xi_0^3 + \xi_1^2 \tag{S37}$$

The eigensolutions at the $\vec{K}$ point are:

$$\lambda_0 = -\frac{\eta_2}{3} + \sqrt[3]{\xi_1 + \sqrt{\xi_2}} + \sqrt[3]{\xi_1 - \sqrt{\xi_2}} \tag{S38}$$

$$\lambda_1 = -\frac{\eta_2}{3} - (\frac{1 - i\sqrt{3}}{2})\sqrt[3]{\xi_1 + \sqrt{\xi_2}} - (\frac{1 + i\sqrt{3}}{2})\sqrt[3]{\xi_1 - \sqrt{\xi_2}} \tag{S39}$$

$$\lambda_2 = -\frac{\eta_2}{3} - (\frac{1 + i\sqrt{3}}{2})\sqrt[3]{\xi_1 + \sqrt{\xi_2}} - (\frac{1 - i\sqrt{3}}{2})\sqrt[3]{\xi_1 - \sqrt{\xi_2}} \tag{S40}$$

$$\lambda_{3,4} = K_1 \pm 2K_4 \tag{S41}$$

APPENDIX B: $\vec{k} \cdot \vec{p}$ ANALYSIS

In Ref. [1] a general expression for the $\vec{k} \cdot \vec{p}$ dispersion relations of silicene and similar materials (such as stanene) has already been obtained near the $\vec{K}'$ point from nearest-neighbor tight-binding relations (see also Refs. [2–5]). Here we will first develop an analogous treatment around $\vec{K}'$, including the additional parameter Δ_{pz} (the difference between the on-site energy of the p_z atomic orbital and that of the p_x and p_y orbitals) that we have considered in our nearest-neighbor tight-binding analysis. Then we will adopt a similar procedure to find the $\vec{k} \cdot \vec{p}$ expressions around the points $\vec{K}$ and Γ. Finally, once we have obtained the $\vec{k} \cdot \vec{p}$ analytical relations near these points, we will numerically find the values of the $\vec{k} \cdot \vec{p}$ parameters which best fit the DFT dispersion relations in those regions of the reciprocal space.

In order to maintain a close analogy with Ref. [1], we will consider a reference frame rotated by 30° in the clockwise direction with respect to that adopted in the previous tight-binding calculations. With this choice, the three vectors which connect each atom A to its nearest neighbor B atoms (in Fig. 1, atoms A and B are represented in blue and

in red, respectively) are:

$$\vec{d}_1 = \frac{a_0}{\sqrt{3}} \begin{bmatrix} \frac{\sqrt{3}}{2} \\ \frac{1}{2} \\ \cot\theta \end{bmatrix}, \quad \vec{d}_2 = \frac{a_0}{\sqrt{3}} \begin{bmatrix} -\frac{\sqrt{3}}{2} \\ \frac{1}{2} \\ \cot\theta \end{bmatrix}, \quad \vec{d}_3 = \frac{a_0}{\sqrt{3}} \begin{bmatrix} 0 \\ -1 \\ \cot\theta \end{bmatrix}, \tag{S42}$$

where $\theta = \arctan[(a_0/\sqrt{3})/(-\Delta)]$ is the angle between the z direction orthogonal to the plane and the bond between an A atom and a nearest-neighbor B atom (in stanene $\theta \approx 107.1°$). In the reciprocal space, $\vec{K}' = [4\pi/(3a_0),\, 0,\, 0]^T$, $\vec{K} = [-4\pi/(3a_0),\, 0,\, 0]^T$, and $\vec{\Gamma} = [0,\, 0,\, 0]^T$.

Using the basis set $\mathcal{V}_1 = \{|s^A\rangle, |p_x^A\rangle, |p_y^A\rangle, |p_z^A\rangle, |s^B\rangle, |p_x^B\rangle, |p_y^B\rangle, |p_z^B\rangle\}$ and the nearest neighbor approach with the Δ_{pz} parameter, the Hamiltonian in the absence of spin-orbit coupling can be written as:

$$\tilde{H}_0(\vec{k}) = \begin{bmatrix} \Delta & 0 & 0 & 0 & h_{15} & h_{16} & h_{17} & h_{18} \\ 0 & 0 & 0 & 0 & -h_{16} & h_{26} & h_{27} & h_{28} \\ 0 & 0 & 0 & 0 & -h_{17} & h_{27} & h_{37} & h_{38} \\ 0 & 0 & 0 & \Delta_{pz} & -h_{18} & h_{28} & h_{38} & h_{48} \\ h_{15}^* & -h_{16}^* & -h_{17}^* & -h_{18}^* & \Delta & 0 & 0 & 0 \\ h_{16}^* & h_{26}^* & h_{27}^* & h_{28}^* & 0 & 0 & 0 & 0 \\ h_{17}^* & h_{27}^* & h_{37}^* & h_{38}^* & 0 & 0 & 0 & 0 \\ h_{18}^* & h_{28}^* & h_{38}^* & h_{48}^* & 0 & 0 & 0 & \Delta_{pz} \end{bmatrix} \tag{S43}$$

where:

$$\begin{aligned} h_{15} &= V_{ss\sigma}\,(e^{i\vec{k}\cdot\vec{d}_1} + e^{i\vec{k}\cdot\vec{d}_2} + e^{i\vec{k}\cdot\vec{d}_3})\,, \\ h_{16} &= \sin\theta\,\frac{\sqrt{3}}{2}\,V_{sp\sigma}\,(e^{i\vec{k}\cdot\vec{d}_1} - e^{i\vec{k}\cdot\vec{d}_2})\,, \\ h_{17} &= \sin\theta\,V_{sp\sigma}\left[\frac{1}{2}\,(e^{i\vec{k}\cdot\vec{d}_1} + e^{i\vec{k}\cdot\vec{d}_2}) - e^{i\vec{k}\cdot\vec{d}_3}\right]\,, \\ h_{18} &= \cos\theta\,V_{sp\sigma}\,(e^{i\vec{k}\cdot\vec{d}_1} + e^{i\vec{k}\cdot\vec{d}_2} + e^{i\vec{k}\cdot\vec{d}_3})\,, \\ h_{26} &= \left[\sin^2\theta\,\frac{3}{4}\,V_{pp\sigma} + \left(1 - \sin^2\theta\,\frac{3}{4}\right)V_{pp\pi}\right](e^{i\vec{k}\cdot\vec{d}_1} + e^{i\vec{k}\cdot\vec{d}_2}) + V_{pp\pi}e^{i\vec{k}\cdot\vec{d}_3}\,, \\ h_{27} &= \sin^2\theta\,\frac{\sqrt{3}}{4}\,(V_{pp\sigma} - V_{pp\pi})\,(e^{i\vec{k}\cdot\vec{d}_1} - e^{i\vec{k}\cdot\vec{d}_2})\,, \\ h_{28} &= \sin\theta\cos\theta\,\frac{\sqrt{3}}{2}\,(V_{pp\sigma} - V_{pp\pi})\,(e^{i\vec{k}\cdot\vec{d}_1} - e^{i\vec{k}\cdot\vec{d}_2})\,, \\ h_{37} &= \left[\sin^2\theta\,\frac{1}{4}\,V_{pp\sigma} + \left(1 - \sin^2\theta\,\frac{1}{4}\right)V_{pp\pi}\right](e^{i\vec{k}\cdot\vec{d}_1} + e^{i\vec{k}\cdot\vec{d}_2}) + \left[\sin^2\theta\,V_{pp\sigma} + \left(1 - \sin^2\theta\right)V_{pp\pi}\right]e^{i\vec{k}\cdot\vec{d}_3}\,, \\ h_{38} &= \sin\theta\cos\theta\,(V_{pp\sigma} - V_{pp\pi})\left[\frac{1}{2}\,(e^{i\vec{k}\cdot\vec{d}_1} + e^{i\vec{k}\cdot\vec{d}_2}) - e^{i\vec{k}\cdot\vec{d}_3}\right]\,, \\ h_{48} &= (\cos^2\theta\,V_{pp\sigma} + \sin^2\theta\,V_{pp\pi})\,(e^{i\vec{k}\cdot\vec{d}_1} + e^{i\vec{k}\cdot\vec{d}_2} + e^{i\vec{k}\cdot\vec{d}_3})\,, \end{aligned} \tag{S44}$$

with $\vec{k} = [k_x,\, k_y,\, 0]^T$ the wave vector of the electrons in stanene. In analogy with Ref. [1], here we have taken as zero energy the value of ϵ_p, i.e. we have subtracted the value of ϵ_p (in our case, equal to 1.7747 eV: see Table 1) to all the elements on the diagonal of the Hamiltonian. Therefore, $\Delta = \epsilon_s - \epsilon_p = -6.4042 - 1.7747 = -8.1789$ eV. At the end of the calculations, the value ϵ_p will be added back to the elements on the diagonal of the final Hamiltonians.

B.1: $\vec{k} \cdot \vec{p}$ analysis at the point $\vec{K}'$

Regarding the $\vec{k} \cdot \vec{p}$ analysis at the point $\vec{K}'$, it is very similar to that performed in Ref. [1] and thus here we report only the main steps, describing the differences with respect to such reference; a detailed explanation can be found in the Supplementary Information.

In $\vec{K}'$ we have that $e^{i\vec{K}'\cdot\vec{d}_1} = e^{i2\pi/3}$, $e^{i\vec{K}'\cdot\vec{d}_2} = e^{-i2\pi/3}$, $e^{i\vec{K}'\cdot\vec{d}_3} = 1$, and therefore the Hamiltonian (S43) becomes:

$$\tilde{H}_0(\vec{K}') = \begin{bmatrix} \Delta & 0 & 0 & 0 & 0 & iV_2' & -V_2' & 0 \\ 0 & 0 & 0 & 0 & -iV_2' & V_1' & -iV_1' & -iV_3' \\ 0 & 0 & 0 & 0 & V_2' & -iV_1' & -V_1' & V_3' \\ 0 & 0 & 0 & \Delta_{pz} & 0 & -iV_3' & V_3' & 0 \\ 0 & iV_2' & V_2' & 0 & \Delta & 0 & 0 & 0 \\ -iV_2' & V_1' & iV_1' & iV_3' & 0 & 0 & 0 & 0 \\ -V_2' & iV_1' & -V_1' & V_3' & 0 & 0 & 0 & 0 \\ 0 & iV_3' & V_3' & 0 & 0 & 0 & 0 & \Delta_{pz} \end{bmatrix} \tag{S45}$$

with

$$V_1' = \frac{3}{4}\sin^2\theta\,(V_{pp\pi} - V_{pp\sigma})\,, \quad V_2' = \frac{3}{2}\sin\theta\,V_{sp\sigma}\,, \quad V_3' = \frac{3}{2}\sin\theta\,\cos\theta\,(V_{pp\pi} - V_{pp\sigma})\,. \tag{S46}$$

This Hamiltonian can be diagonalized performing a basis change through the unitary matrix $\tilde{U}$

$$\tilde{U} = \begin{bmatrix} u_{21} & 0 & u_{22} & 0 & u_{23} & 0 & 0 & 0 \\ 0 & -\frac{u_{31}}{\sqrt{2}} & 0 & -\frac{u_{32}}{\sqrt{2}} & 0 & -\frac{u_{33}}{\sqrt{2}} & -\frac{1}{2} & \frac{1}{2} \\ 0 & -\frac{iu_{31}}{\sqrt{2}} & 0 & -\frac{iu_{32}}{\sqrt{2}} & 0 & -\frac{iu_{33}}{\sqrt{2}} & \frac{i}{2} & -\frac{i}{2} \\ u_{11} & 0 & u_{12} & 0 & u_{13} & 0 & 0 & 0 \\ 0 & -u_{21} & 0 & -u_{22} & 0 & -u_{23} & 0 & 0 \\ \frac{u_{31}}{\sqrt{2}} & 0 & \frac{u_{32}}{\sqrt{2}} & 0 & \frac{u_{33}}{\sqrt{2}} & 0 & -\frac{1}{2} & -\frac{1}{2} \\ -\frac{iu_{31}}{\sqrt{2}} & 0 & -\frac{iu_{32}}{\sqrt{2}} & 0 & -\frac{iu_{33}}{\sqrt{2}} & 0 & -\frac{i}{2} & -\frac{i}{2} \\ 0 & u_{11} & 0 & u_{12} & 0 & u_{13} & 0 & 0 \end{bmatrix}. \tag{S47}$$

Indeed, we have that $(\tilde{U})^\dagger \tilde{H}_0(\vec{K}')\tilde{U} = H_0'(\vec{K}')$, with

$$H_0'(\vec{K}') = \begin{bmatrix} \epsilon_1 & 0 & 0 & 0 & 0 & 0 & 0 & 0 \\ 0 & \epsilon_1 & 0 & 0 & 0 & 0 & 0 & 0 \\ 0 & 0 & \epsilon_2 & 0 & 0 & 0 & 0 & 0 \\ 0 & 0 & 0 & \epsilon_2 & 0 & 0 & 0 & 0 \\ 0 & 0 & 0 & 0 & \epsilon_3 & 0 & 0 & 0 \\ 0 & 0 & 0 & 0 & 0 & \epsilon_3 & 0 & 0 \\ 0 & 0 & 0 & 0 & 0 & 0 & V_1 & 0 \\ 0 & 0 & 0 & 0 & 0 & 0 & 0 & -V_1 \end{bmatrix}. \tag{S48}$$

With respect to Ref. [1], in our analysis we have included the further tight-binding parameter Δ_{pz}. As a consequence, here the energies ϵ_i (with $i = 1, 2, 3$, where $\epsilon_3 < \epsilon_1 < \epsilon_2$) are the roots of the equation

$$\epsilon^3 - (\Delta + \Delta_{pz})\epsilon^2 - (-\Delta_{pz}\Delta + V_2^2 + V_3^2)\epsilon + \Delta_{pz}V_2^2 + \Delta V_3^2 = 0\,, \tag{S49}$$

(with $V_1 = 2V_1'$, $V_2 = \sqrt{2}V_2'$, and $V_3 = \sqrt{2}V_3'$), while the quantities u are defined in this way:

$$u_{1i} = \frac{1}{\alpha_i}\,, \quad u_{2i} = \frac{V_2(\epsilon_i - \Delta_{pz})}{\alpha_i(\Delta - \epsilon_i)V_3}\,, \quad u_{3i} = \frac{i(\epsilon_i - \Delta_{pz})}{\alpha_i V_3}\,, \tag{S50}$$

with

$$\alpha_i = \sqrt{1 + \left(\frac{V_2(\epsilon_i - \Delta_{pz})}{(\Delta - \epsilon_i)V_3}\right)^2 + \left(\frac{\epsilon_i - \Delta_{pz}}{V_3}\right)^2}\,. \tag{S51}$$

Substituting the values of the NNTB parameters of Table 1 into the expressions of V_1, V_2 and V_3 and solving Eq. (S49), we obtain that $\epsilon_3 = -9.8374$ eV (double degenerate), $V_1 = -4.1959$ eV, $\epsilon_1 = -1.7430$ eV (double degenerate), $\epsilon_2 = 2.4555$ eV (double degenerate), $-V_1 = 4.1959$ eV. Therefore, the Fermi energy ϵ_F (which leaves half of the

energy levels occupied, i.e. with values lower than ϵ_F, and half unoccupied, i.e. with values higher than ϵ_F) is located at ϵ_1 and thus we have focused on the states corresponding to this eigenvalue.

In order to obtain the $\vec{k}\cdot\vec{p}$ Hamiltonian of stanene around $\vec{K}'$, we have performed a first-order Taylor expansion around $\vec{K}'$ of all the elements of the tight-binding Hamiltonian of Eq. (S43), obtaining the matrix $\tilde{H}_o^{(\text{exp})}(\vec{\kappa})$ (where $\vec{\kappa} = \vec{k} - \vec{K}'$ represents the distance in the reciprocal space between $\vec{K}'$ and $\vec{k}$). Then, performing a basis change through the unitary matrix $\tilde{U}$, we have obtained $H_0'^{(\text{exp})}(\vec{\kappa}) = (\tilde{U})^\dagger \tilde{H}_0^{(\text{exp})}(\vec{\kappa})\,\tilde{U}$. The $\vec{k}\cdot\vec{p}$ Hamiltonian is obtained projecting this matrix onto the two states which have energy at $\vec{K}'$ equal to ϵ_1, i.e. considering the 2×2 submatrix of $H_0'^{(\text{exp})}(\vec{\kappa})$ given by the intersection of its first two rows and of its first two columns. We obtain:

$$H_{\vec{K}'}(\vec{\kappa}) = \begin{bmatrix} \epsilon_1 & \gamma(\kappa_x + i\kappa_y) \\ \gamma(\kappa_x - i\kappa_y) & \epsilon_1 \end{bmatrix}, \tag{S52}$$

where γ (which coincides with $\hbar v_F$, with $\hbar$ the reduced Planck constant and v_F the Fermi velocity) is equal to

$$\gamma = -\frac{\sqrt{3}a_0}{2}\left[u_{11}^2(V_{pp\pi}\sin^2\theta + V_{pp\sigma}\cos^2\theta) - u_{21}^2 V_{ss\sigma} + 2u_{11}u_{21}\cos\theta V_{sp\sigma} - \frac{1}{2}|u_{31}|^2\sin^2\theta(V_{pp\sigma} - V_{pp\pi})\right]. \tag{S53}$$

If we include the spin-orbit coupling (neglecting the Rashba term), the total Hamiltonian on the basis $\mathcal{V}_1\otimes\{|\uparrow\rangle, |\downarrow\rangle\}$ can be written as $\tilde{H}_0(\vec{k})\otimes I_2 + \tilde{H}_{SO}$, where

$$\tilde{H}_{SO} = \begin{bmatrix} \hat{H}_{SO} & 0 \\ 0 & \hat{H}_{SO} \end{bmatrix}, \quad \hat{H}_{SO} = \frac{\Delta_{SO}}{3}\begin{bmatrix} 0&0&0&0&0&0&0&0 \\ 0&0&0&0&0&0&0&0 \\ 0&0&0&0&-i&0&0&1 \\ 0&0&0&0&0&i&-1&0 \\ 0&0&i&0&0&0&0&-i \\ 0&0&0&-i&0&0&-i&0 \\ 0&0&0&-1&0&i&0&0 \\ 0&0&1&0&i&0&0&0 \end{bmatrix}. \tag{S54}$$

Repeating the previous treatment on this extended Hamiltonian, we have first performed a first-order Taylor expansion in $\vec{\kappa}$ around $\vec{K}'$ of its elements. Then we have changed our basis through the unitary matrix $\tilde{U}\otimes I_2$, obtaining

$$(\tilde{U}\otimes I_2)^\dagger(\tilde{H}_0^{(\text{exp})}(\vec{\kappa})\otimes I_2 + \tilde{H}_{SO})(\tilde{U}\otimes I_2) = H_0'^{(\text{exp})}(\vec{\kappa})\otimes I_2 + (\tilde{U}\otimes I_2)^\dagger\tilde{H}_{SO}(\tilde{U}\otimes I_2)\,. \tag{S55}$$

Finally, we have considered the 4×4 submatrix given by the intersection of its first 4 rows and of its first 4 columns (which corresponds to projecting the Hamiltonian onto the two basis states with energy ϵ_1 at $\vec{K}'$ in the absence of spin-orbit, taken with spin up and spin down). We have obtained this $\vec{k}\cdot\vec{p}$ Hamiltonian:

$$\begin{bmatrix} \epsilon_1 & 0 & \gamma(\kappa_x+i\kappa_y) & 0 \\ 0 & \epsilon_1 & 0 & \gamma(\kappa_x+i\kappa_y) \\ \gamma(\kappa_x-i\kappa_y) & 0 & \epsilon_1 & 0 \\ 0 & \gamma(\kappa_x-i\kappa_y) & 0 & \epsilon_1 \end{bmatrix} + \lambda_{SO}^{1st}\begin{bmatrix} -1&0&0&0 \\ 0&1&0&0 \\ 0&0&1&0 \\ 0&0&0&-1 \end{bmatrix}, \tag{S56}$$

with

$$\lambda_{SO}^{1st} = \frac{\Delta_{SO}}{3}|u_{31}|^2\,. \tag{S57}$$

Following the method described in the Appendix B of Ref. [1], we have then partially recovered the effect, on the 4 bands that we have considered in our projection, of the 12 bands that we have instead discarded, through a term $-H_n(H_\sigma - \epsilon_1 I_{12})^{-1}H_n^\dagger$, where I_{12} is the 12×12 identity matrix, H_n is given by the intersection of the first 4 rows and of the 5th-to-16th columns of $(\tilde{U}\otimes I_2)^\dagger\tilde{H}_{SO}(\tilde{U}\otimes I_2)$, and H_σ is the intersection of the 5th-to-16th rows and of the 5th-to-16th columns of $H_0'(\vec{K}')\otimes I_2$. We obtain the following quantity (which represents an effective second-order spin-orbit interaction):

$$H_{so}^{2nd} = \begin{bmatrix} b_1&0&0&0 \\ 0&b_2&0&0 \\ 0&0&b_2&0 \\ 0&0&0&b_1 \end{bmatrix}, \tag{S58}$$

with

$$b_1 = \left(\frac{\Delta_{SO}}{3}\right)^2 \left(\frac{2\,u_{12}^2|u_{31}|^2 + 4\,u_{11}u_{12}u_{31}u_{32} + 2\,u_{11}^2|u_{32}|^2 + |u_{31}|^2|u_{32}|^2}{\epsilon_1 - \epsilon_2} + \right.$$
$$\left. + \frac{2\,u_{13}^2|u_{31}|^2 + 4\,u_{11}u_{13}u_{31}u_{33} + 2\,u_{11}^2|u_{33}|^2 + |u_{31}|^2|u_{33}|^2}{\epsilon_1 - \epsilon_3}\right), \tag{S59}$$
$$b_2 = \left(\frac{\Delta_{SO}}{3}\right)^2 \left(\frac{|u_{31}|^2|u_{32}|^2}{\epsilon_1 - \epsilon_2} + \frac{|u_{31}|^2|u_{33}|^2}{\epsilon_1 - \epsilon_3} + \frac{2\,\epsilon_1\,u_{11}^2}{\epsilon_1^2 - V_1^2}\right).$$

Adding Eq. (S58) to Eq. (S56), we obtain the Hamiltonian

$$H_{\vec{K}'}(\vec{\kappa}) \otimes I_2 + \begin{bmatrix} d_1 & 0 & 0 & 0 \\ 0 & d_2 & 0 & 0 \\ 0 & 0 & d_2 & 0 \\ 0 & 0 & 0 & d_1 \end{bmatrix}, \tag{S60}$$

where

$$d_1 = b_1 - \lambda_{SO}^{1st}\,, \quad d_2 = b_2 + \lambda_{SO}^{1st}\,. \tag{S61}$$

B.2: $\vec{k}\cdot\vec{p}$ analysis at the point $\vec{K}$

The $\vec{k}\cdot\vec{p}$ analysis around $\vec{K}$ is very similar to that around $\vec{K}'$. Here we summarize it, reporting a detailed explanation in the Supplementary Information.

In $\vec{K}$ we have that $e^{i\vec{K}\cdot\vec{d}_1} = e^{-i2\pi/3}$, $e^{i\vec{K}\cdot\vec{d}_2} = e^{i2\pi/3}$, $e^{i\vec{K}\cdot\vec{d}_3} = 1$, and therefore the Hamiltonian (S43) becomes:

$$\tilde{H}_0(\vec{K}) = \begin{bmatrix} \Delta & 0 & 0 & 0 & 0 & -iV_2' & -V_2' & 0 \\ 0 & 0 & 0 & 0 & iV_2' & V_1' & iV_1' & iV_3' \\ 0 & 0 & 0 & 0 & V_2' & iV_1' & -V_1' & V_3' \\ 0 & 0 & 0 & \Delta_{pz} & 0 & iV_3' & V_3' & 0 \\ 0 & -iV_2' & V_2' & 0 & \Delta & 0 & 0 & 0 \\ iV_2' & V_1' & -iV_1' & -iV_3' & 0 & 0 & 0 & 0 \\ -V_2' & -iV_1' & -V_1' & V_3' & 0 & 0 & 0 & 0 \\ 0 & -iV_3' & V_3' & 0 & 0 & 0 & 0 & \Delta_{pz} \end{bmatrix}. \tag{S62}$$

This hermitian matrix is the complex conjugate of Eq. (S45) and thus can be diagonalized by the complex conjugate of the unitary matrix $\tilde{U}$, that we call $\tilde{W}$:

$$\tilde{W} = \begin{bmatrix} w_{21} & 0 & w_{22} & 0 & w_{23} & 0 & 0 & 0 \\ 0 & -\frac{w_{31}}{\sqrt{2}} & 0 & -\frac{w_{32}}{\sqrt{2}} & 0 & -\frac{w_{33}}{\sqrt{2}} & -\frac{1}{2} & \frac{1}{2} \\ 0 & \frac{iw_{31}}{\sqrt{2}} & 0 & \frac{iw_{32}}{\sqrt{2}} & 0 & \frac{iw_{33}}{\sqrt{2}} & -\frac{i}{2} & \frac{i}{2} \\ w_{11} & 0 & w_{12} & 0 & w_{13} & 0 & 0 & 0 \\ 0 & -w_{21} & 0 & -w_{22} & 0 & -w_{23} & 0 & 0 \\ \frac{w_{31}}{\sqrt{2}} & 0 & \frac{w_{32}}{\sqrt{2}} & 0 & \frac{w_{33}}{\sqrt{2}} & 0 & -\frac{1}{2} & -\frac{1}{2} \\ \frac{iw_{31}}{\sqrt{2}} & 0 & \frac{iw_{32}}{\sqrt{2}} & 0 & \frac{iw_{33}}{\sqrt{2}} & 0 & \frac{i}{2} & \frac{i}{2} \\ 0 & w_{11} & 0 & w_{12} & 0 & w_{13} & 0 & 0 \end{bmatrix}, \tag{S63}$$

where $w_{ij} = u_{ij}^*$. We have that $(\tilde{W})^\dagger \tilde{H}_0(\vec{K})\tilde{W} = H_0'(\vec{K})$, with

$$H_0'(\vec{K}) = \begin{bmatrix} \epsilon_1 & 0 & 0 & 0 & 0 & 0 & 0 & 0 \\ 0 & \epsilon_1 & 0 & 0 & 0 & 0 & 0 & 0 \\ 0 & 0 & \epsilon_2 & 0 & 0 & 0 & 0 & 0 \\ 0 & 0 & 0 & \epsilon_2 & 0 & 0 & 0 & 0 \\ 0 & 0 & 0 & 0 & \epsilon_3 & 0 & 0 & 0 \\ 0 & 0 & 0 & 0 & 0 & \epsilon_3 & 0 & 0 \\ 0 & 0 & 0 & 0 & 0 & 0 & V_1 & 0 \\ 0 & 0 & 0 & 0 & 0 & 0 & 0 & -V_1 \end{bmatrix} . \tag{S64}$$

The energies ϵ_i (with $i = 1, 2, 3$, where $\epsilon_3 < \epsilon_1 < \epsilon_2$) are still the roots of Eq. (S49) and have the same values as in $\vec{K}'$. Therefore, also in this case we have focused our analysis on the states corresponding to the energy ϵ_1.

The $\vec{k} \cdot \vec{p}$ Hamiltonian around $\vec{K}$ has been obtained in a way similar to what we have done near $\vec{K}'$. We have performed a first-order Taylor expansion around $\vec{K}$ of all the elements of the tight-binding Hamiltonian of Eq. (S43), obtaining the matrix $\tilde{H}_o^{(\exp)}(\vec{\kappa})$, with $\vec{\kappa} = \vec{k} - \vec{K}$. Then we have performed a basis change through the unitary matrix $\tilde{W}$, obtaining $H_0'^{(\exp)}(\vec{\kappa}) = (\tilde{W})^\dagger \tilde{H}_0^{(\exp)}(\vec{\kappa})\,\tilde{W}$. Projecting this matrix onto the two states corresponding to ϵ_1, i.e. considering the 2×2 submatrix of $H_0'^{(\exp)}(\vec{\kappa})$ given by the intersection of its first two rows and of its first two columns, we have finally obtained the $\vec{k} \cdot \vec{p}$ Hamiltonian around $\vec{K}$:

$$H_{\vec{K}}(\vec{\kappa}) = \begin{bmatrix} \epsilon_1 & -\gamma(\kappa_x - i\kappa_y) \\ -\gamma(\kappa_x + i\kappa_y) & \epsilon_1 \end{bmatrix} , \tag{S65}$$

where γ is still given by Eq. (S53).

If we include the spin-orbit interaction (neglecting the Rashba term), the total Hamiltonian on the basis $\mathcal{V}_1 \otimes \{|\uparrow\rangle, |\downarrow\rangle\}$ is $\tilde{H}_0(\vec{k}) \otimes I_2 + \tilde{H}_{SO}$, where $\tilde{H}_{SO}$ is given by Eq. (S54). We have first performed a first-order Taylor expansion in $\vec{\kappa}$ around $\vec{K}$ of its elements. Then we have changed our basis through the unitary matrix $\tilde{W} \otimes I_2$, obtaining

$$(\tilde{W} \otimes I_2)^\dagger (\tilde{H}_0^{(\exp)}(\vec{\kappa}) \otimes I_2 + \tilde{H}_{SO})(\tilde{W} \otimes I_2) = H_0'^{(\exp)}(\vec{\kappa}) \otimes I_2 + (\tilde{W} \otimes I_2)^\dagger \tilde{H}_{SO} (\tilde{W} \otimes I_2) . \tag{S66}$$

Finally, we have considered the 4×4 submatrix given by the intersection of its first 4 rows and of its first 4 columns. At the end, we have obtained this $\vec{k} \cdot \vec{p}$ Hamiltonian:

$$\begin{bmatrix} \epsilon_1 & 0 & -\gamma(\kappa_x - i\kappa_y) & 0 \\ 0 & \epsilon_1 & 0 & -\gamma(\kappa_x - i\kappa_y) \\ -\gamma(\kappa_x + i\kappa_y) & 0 & \epsilon_1 & 0 \\ 0 & -\gamma(\kappa_x + i\kappa_y) & 0 & \epsilon_1 \end{bmatrix} + \lambda_{SO}^{1st} \begin{bmatrix} 1 & 0 & 0 & 0 \\ 0 & -1 & 0 & 0 \\ 0 & 0 & -1 & 0 \\ 0 & 0 & 0 & 1 \end{bmatrix} , \tag{S67}$$

with

$$\lambda_{SO}^{1st} = \frac{\Delta_{SO}}{3} |w_{31}|^2 . \tag{S68}$$

(which coincides with Eq. (S57)).

Also in this case, we can improve our effective Hamiltonian adopting the method described in the Appendix B of Ref. [1]. We obtain the following effective second-order spin-orbit interaction:

$$H_{so}^{2nd} = \begin{bmatrix} b_2 & 0 & 0 & 0 \\ 0 & b_1 & 0 & 0 \\ 0 & 0 & b_1 & 0 \\ 0 & 0 & 0 & b_2 \end{bmatrix} , \tag{S69}$$

where b_1 and b_2 are defined in Eq. (S59). Adding Eq. (S69) to Eq. (S67), we derive the Hamiltonian

$$H_{\vec{K}}(\vec{\kappa}) \otimes I_2 + \begin{bmatrix} d_2 & 0 & 0 & 0 \\ 0 & d_1 & 0 & 0 \\ 0 & 0 & d_1 & 0 \\ 0 & 0 & 0 & d_2 \end{bmatrix} , \tag{S70}$$

where d_1 and d_2 are defined in Eq. (S61).

B.3: $\vec{k} \cdot \vec{p}$ analysis at the point $\vec{\Gamma}$

Let us now describe in more detail the derivation of the $\vec{k} \cdot \vec{p}$ Hamiltonian around $\vec{\Gamma}$.

We first diagonalize the tight-binding Hamiltonian exactly at the point $\vec{\Gamma} = [\,0,\,0,\,0\,]^T$. In $\vec{\Gamma}$ we have that $e^{i\vec{\Gamma}\cdot\vec{d}_1} = 1$, $e^{i\vec{\Gamma}\cdot\vec{d}_2} = 1$, $e^{i\vec{\Gamma}\cdot\vec{d}_3} = 1$, and therefore the Hamiltonian (S43) reads:

$$\tilde{H}_0(\vec{\Gamma}) = \begin{bmatrix} \Delta & 0 & 0 & 0 & V_4 & 0 & 0 & V_5 \\ 0 & 0 & 0 & 0 & 0 & V_6 & 0 & 0 \\ 0 & 0 & 0 & 0 & 0 & 0 & V_6 & 0 \\ 0 & 0 & 0 & \Delta_{pz} & -V_5 & 0 & 0 & V_7 \\ V_4 & 0 & 0 & -V_5 & \Delta & 0 & 0 & 0 \\ 0 & V_6 & 0 & 0 & 0 & 0 & 0 & 0 \\ 0 & 0 & V_6 & 0 & 0 & 0 & 0 & 0 \\ V_5 & 0 & 0 & V_7 & 0 & 0 & 0 & \Delta_{pz} \end{bmatrix} \tag{S71}$$

with

$$\begin{gathered} V_4 = 3\,V_{ss\sigma}\,, \quad V_5 = 3\,\cos\theta\,V_{sp\sigma}\,, \\ V_6 = \frac{3}{2}\,\sin^2\theta\,V_{pp\sigma} + \left(3 - \frac{3}{2}\,\sin^2\theta\right)\,V_{pp\pi}\,, \quad V_7 = 3\,\cos^2\theta\,V_{pp\sigma} + 3\,\sin^2\theta\,V_{pp\pi}\,. \end{gathered} \tag{S72}$$

Let us now move from the basis set $\mathcal{V}_1$ to the basis set $\mathcal{M}_2 = \{|v_1\rangle, |v_2\rangle, |v_3\rangle, |v_4\rangle, |v_5\rangle, |v_6\rangle, |v_7\rangle, |v_8\rangle)$, where

$$\begin{aligned} &|v_1\rangle = \frac{1}{\sqrt{2}}(|s^A\rangle + |s^B\rangle)\,, \quad |v_2\rangle = \frac{1}{\sqrt{2}}(|p_z^A\rangle - |p_z^B\rangle)\,, \quad |v_3\rangle = \frac{1}{\sqrt{2}}(|s^A\rangle - |s^B\rangle)\,, \quad |v_4\rangle = \frac{1}{\sqrt{2}}(|p_z^A\rangle + |p_z^B\rangle) \\ &|v_5\rangle = \frac{1}{\sqrt{2}}(|p_x^A\rangle + |p_x^B\rangle)\,, \quad |v_6\rangle = \frac{1}{\sqrt{2}}(|p_y^A\rangle + |p_y^B\rangle)\,, \quad |v_7\rangle = \frac{1}{\sqrt{2}}(|p_x^A\rangle - |p_x^B\rangle)\,, \quad |v_8\rangle = \frac{1}{\sqrt{2}}(|p_y^A\rangle - |p_y^B\rangle) \end{aligned} \tag{S73}$$

through the unitary transformation

$$\tilde{Q}_1 = \frac{1}{\sqrt{2}} \begin{bmatrix} 1 & 0 & 1 & 0 & 0 & 0 & 0 & 0 \\ 0 & 0 & 0 & 0 & 1 & 0 & 1 & 0 \\ 0 & 0 & 0 & 0 & 0 & 1 & 0 & 1 \\ 0 & 1 & 0 & 1 & 0 & 0 & 0 & 0 \\ 1 & 0 & -1 & 0 & 0 & 0 & 0 & 0 \\ 0 & 0 & 0 & 0 & 1 & 0 & -1 & 0 \\ 0 & 0 & 0 & 0 & 0 & 1 & 0 & -1 \\ 0 & -1 & 0 & 1 & 0 & 0 & 0 & 0 \end{bmatrix}\,. \tag{S74}$$

The Hamiltonian becomes

$$H_1(\vec{\Gamma}) = (\tilde{Q}_1)^\dagger\,\tilde{H}_0(\vec{\Gamma})\,\tilde{Q}_1 = \begin{bmatrix} \Delta + V_4 & -V_5 & 0 & 0 & 0 & 0 & 0 & 0 \\ -V_5 & \Delta_{pz} - V_7 & 0 & 0 & 0 & 0 & 0 & 0 \\ 0 & 0 & \Delta - V_4 & V_5 & 0 & 0 & 0 & 0 \\ 0 & 0 & V_5 & \Delta_{pz} + V_7 & 0 & 0 & 0 & 0 \\ 0 & 0 & 0 & 0 & V_6 & 0 & 0 & 0 \\ 0 & 0 & 0 & 0 & 0 & V_6 & 0 & 0 \\ 0 & 0 & 0 & 0 & 0 & 0 & -V_6 & 0 \\ 0 & 0 & 0 & 0 & 0 & 0 & 0 & -V_6 \end{bmatrix}\,. \tag{S75}$$

This Hamiltonian is a block diagonal matrix with the following blocks on the diagonal:

$$H_A^{\vec{\Gamma}} = \begin{bmatrix} \Delta + V_4 & -V_5 \\ -V_5 & \Delta_{pz} - V_7 \end{bmatrix}\,, \quad H_B^{\vec{\Gamma}} = \begin{bmatrix} \Delta - V_4 & V_5 \\ V_5 & \Delta_{pz} + V_7 \end{bmatrix}\,, \quad V_6\,, \quad V_6\,, \quad -V_6\,, \quad -V_6\,. \tag{S76}$$

The eigenvalues ϵ'_i (with $i = 1, 2$, where $\epsilon'_2 < \epsilon'_1$) of $H_A^{\vec{\Gamma}}$ are the roots of the equation $\det(H_A^{\vec{\Gamma}} - \epsilon I_2) = 0$ (where I_2 is the 2×2 identity matrix), i.e. of

$$\epsilon^2 + \epsilon\,[\,-(\Delta + V_4) - (\Delta_{pz} - V_7)\,] + [\,(\Delta + V_4)(\Delta_{pz} - V_7) - V_5^2\,] = 0\,, \tag{S77}$$

while the corresponding orthonormalized eigenvectors are

$$\begin{bmatrix} \frac{1}{\beta_i} \\ -\frac{1}{\beta_i} \frac{V_5}{\epsilon'_i - \Delta_{pz} + V_7} \end{bmatrix} \equiv \begin{bmatrix} q_{1i} \\ q_{2i} \end{bmatrix} \tag{S78}$$

where

$$\beta_i = \sqrt{1 + \left(\frac{V_5}{\epsilon'_i - \Delta_{pz} + V_7} \right)^2} \tag{S79}$$

(with $i = 1, 2$).
The matrix of the eigenvectors Q_A diagonalizes $H_A^{\vec{\Gamma}}$, i.e. $Q_A^\dagger H_A^{\vec{\Gamma}} Q_A = \Lambda'_A$, with

$$Q_A = \begin{bmatrix} q_{11} & q_{12} \\ q_{21} & q_{22} \end{bmatrix}, \quad \Lambda'_A = \begin{bmatrix} \epsilon'_1 & 0 \\ 0 & \epsilon'_2 \end{bmatrix}. \tag{S80}$$

In a similar way, the eigenvalues ϵ'_i (with $i = 3, 4$, where $\epsilon'_4 < \epsilon'_3$) of $H_B^{\vec{\Gamma}}$ are the roots of the equation $\det(H_B^{\vec{\Gamma}} - \epsilon I_2) = 0$, i.e. of

$$\epsilon^2 + \epsilon \,[\, -(\Delta - V_4) - (\Delta_{pz} + V_7) \,] + [\, (\Delta - V_4)(\Delta_{pz} + V_7) - V_5^2 \,] = 0\,, \tag{S81}$$

while the corresponding orthonormalized eigenvectors are

$$\begin{bmatrix} \frac{1}{\beta_i} \\ \frac{1}{\beta_i} \frac{V_5}{\epsilon'_i - \Delta_{pz} - V_7} \end{bmatrix} \equiv \begin{bmatrix} q_{3i} \\ q_{4i} \end{bmatrix} \tag{S82}$$

where

$$\beta_i = \sqrt{1 + \left(\frac{V_5}{\epsilon'_i - \Delta_{pz} - V_7} \right)^2} \tag{S83}$$

(with $i = 3, 4$).
The matrix of the eigenvectors Q_B diagonalizes $H_B^{\vec{\Gamma}}$, i.e. $Q_B^\dagger H_B^{\vec{\Gamma}} Q_B = \Lambda'_B$, with

$$Q_B = \begin{bmatrix} q_{33} & q_{34} \\ q_{43} & q_{44} \end{bmatrix}, \quad \Lambda'_B = \begin{bmatrix} \epsilon'_3 & 0 \\ 0 & \epsilon'_4 \end{bmatrix}. \tag{S84}$$

Therefore, $(Q'_2)^\dagger H_1(\vec{\Gamma}) Q'_2 = H_2(\vec{\Gamma})$, where

$$Q'_2 = \begin{bmatrix} q_{11} & q_{12} & 0 & 0 & 0 & 0 & 0 & 0 \\ q_{21} & q_{22} & 0 & 0 & 0 & 0 & 0 & 0 \\ 0 & 0 & q_{33} & q_{34} & 0 & 0 & 0 & 0 \\ 0 & 0 & q_{43} & q_{44} & 0 & 0 & 0 & 0 \\ 0 & 0 & 0 & 0 & 1 & 0 & 0 & 0 \\ 0 & 0 & 0 & 0 & 0 & 1 & 0 & 0 \\ 0 & 0 & 0 & 0 & 0 & 0 & 1 & 0 \\ 0 & 0 & 0 & 0 & 0 & 0 & 0 & 1 \end{bmatrix}, \quad H_2(\vec{\Gamma}) = \begin{bmatrix} \epsilon'_1 & 0 & 0 & 0 & 0 & 0 & 0 & 0 \\ 0 & \epsilon'_2 & 0 & 0 & 0 & 0 & 0 & 0 \\ 0 & 0 & \epsilon'_3 & 0 & 0 & 0 & 0 & 0 \\ 0 & 0 & 0 & \epsilon'_4 & 0 & 0 & 0 & 0 \\ 0 & 0 & 0 & 0 & V_6 & 0 & 0 & 0 \\ 0 & 0 & 0 & 0 & 0 & V_6 & 0 & 0 \\ 0 & 0 & 0 & 0 & 0 & 0 & -V_6 & 0 \\ 0 & 0 & 0 & 0 & 0 & 0 & 0 & -V_6 \end{bmatrix}. \tag{S85}$$

In this way, we have moved from the basis set $\mathcal{M}_2$ to the basis set $\mathcal{M}_3 = \{|\tau_1\rangle, |\tau_2\rangle, |\tau_3\rangle, |\tau_4\rangle, |\tau_5\rangle, |\tau_6\rangle, |\tau_7\rangle, |\tau_8\rangle\}$, where $|\tau_1\rangle = q_{11}|v_1\rangle + q_{21}|v_2\rangle$, $|\tau_2\rangle = q_{12}|v_1\rangle + q_{22}|v_2\rangle$, $|\tau_3\rangle = q_{33}|v_3\rangle + q_{43}|v_4\rangle$, $|\tau_4\rangle = q_{34}|v_3\rangle + q_{44}|v_4\rangle$, $|\tau_5\rangle = |v_5\rangle$, $|\tau_6\rangle = |v_6\rangle$, $|\tau_7\rangle = |v_7\rangle$, and $|\tau_8\rangle = |v_8\rangle$.
Substituting the values of the NNTB parameters of Table 1 into the expressions of V_4, V_5, V_6 and V_7, and solving Eqs. (S77) and (S81), we obtain that $\epsilon'_2 = -12.0655$ eV, $\epsilon'_4 = -5.4436$ eV, $-V_6 = -2.1652$ eV (double degenerate), $\epsilon'_3 = -1.2715$ eV, $\epsilon'_1 = 0.5309$ eV, $V_6 = 2.1652$ eV (double degenerate). Therefore, the Fermi energy ϵ_F (which leaves half of the energy levels occupied, i.e. with values lower than ϵ_F, and half unoccupied, i.e. with values higher than ϵ_F) is located between $-V_6$ and ϵ'_3. In our analysis around $\vec{\Gamma}$ we have focused on the states nearest to the Fermi energy, and in particular, as we will discuss in the following, on the three states with energy in $\vec{\Gamma}$ equal to $-V_6$ (double

degenerate) and ϵ_3'. It is therefore useful to permute the position of the basis states in such a way as to put these 3 states in the first positions, through a final change of basis $(Q_2'')^\dagger H_2(\vec{\Gamma}) Q_2'' = H_0'(\vec{\Gamma})$, where

$$Q_2'' = \begin{bmatrix} 0&0&0&1&0&0&0&0 \\ 0&0&0&0&1&0&0&0 \\ 1&0&0&0&0&0&0&0 \\ 0&0&0&0&0&1&0&0 \\ 0&0&0&0&0&0&1&0 \\ 0&0&0&0&0&0&0&1 \\ 0&1&0&0&0&0&0&0 \\ 0&0&1&0&0&0&0&0 \end{bmatrix}, \quad H_0'(\vec{\Gamma}) = \begin{bmatrix} \epsilon_3' & 0 & 0 & 0 & 0 & 0 & 0 & 0 \\ 0 & -V_6 & 0 & 0 & 0 & 0 & 0 & 0 \\ 0 & 0 & -V_6 & 0 & 0 & 0 & 0 & 0 \\ 0 & 0 & 0 & \epsilon_1' & 0 & 0 & 0 & 0 \\ 0 & 0 & 0 & 0 & \epsilon_2' & 0 & 0 & 0 \\ 0 & 0 & 0 & 0 & 0 & \epsilon_4' & 0 & 0 \\ 0 & 0 & 0 & 0 & 0 & 0 & V_6 & 0 \\ 0 & 0 & 0 & 0 & 0 & 0 & 0 & V_6 \end{bmatrix}, \tag{S86}$$

in this way moving from the basis set $\mathcal{M}_3$ to the basis set $\mathcal{M}_4 = \{|\tau_3\rangle, |\tau_7\rangle, |\tau_8\rangle, |\tau_1\rangle, |\tau_2\rangle, |\tau_4\rangle, |\tau_5\rangle, |\tau_6\rangle\}$.
The overall unitary matrix $\tilde{Q}$ corresponding to the basis change from $\mathcal{V}_1$ to $\mathcal{M}_4$, i.e. such that $(\tilde{Q})^\dagger \tilde{H}_0(\vec{\Gamma})\tilde{Q} = H_0'(\vec{\Gamma})$, is

$$\tilde{Q} = \tilde{Q}_1 Q_2' Q_2'' = \frac{1}{\sqrt{2}} \begin{bmatrix} q_{33} & 0 & 0 & q_{11} & q_{12} & q_{34} & 0 & 0 \\ 0 & 1 & 0 & 0 & 0 & 0 & 1 & 0 \\ 0 & 0 & 1 & 0 & 0 & 0 & 0 & 1 \\ q_{43} & 0 & 0 & q_{21} & q_{22} & q_{44} & 0 & 0 \\ -q_{33} & 0 & 0 & q_{11} & q_{12} & -q_{34} & 0 & 0 \\ 0 & -1 & 0 & 0 & 0 & 0 & 1 & 0 \\ 0 & 0 & -1 & 0 & 0 & 0 & 0 & 1 \\ q_{43} & 0 & 0 & -q_{21} & -q_{22} & q_{44} & 0 & 0 \end{bmatrix}. \tag{S87}$$

In analogy with Ref. [1], we have obtained the $\vec{k}\cdot\vec{p}$ Hamiltonian of stanene around $\vec{\Gamma}$ performing a low-order expansion around $\vec{\Gamma}$ of the tight-binding Hamiltonian of Eq. (S43) and reducing the matrix size through projection onto the subset of states we are mainly interested in (i.e., those with energy nearest to the Fermi energy).
More in detail, in $\tilde{H}_o(\vec{k})$ (see Eq. (S43)) we have substituted each occurrence of $\vec{k}$ with $\vec{\Gamma} + \vec{\kappa}$ (where $\vec{\kappa} = \vec{k} - \vec{\Gamma}$ represents the distance in the reciprocal space between $\vec{\Gamma}$ and $\vec{k}$); then we have performed a Taylor expansion around $\vec{\kappa} = 0$ (which corresponds to $\vec{k} = \vec{\Gamma}$) of all the elements of the matrix. Since we are interested only in the behavior of the dispersion relations near $\vec{\Gamma}$ (i.e. for small values of $\vec{\kappa}$) and from the DFT and tight-binding results it appears that the dispersion relations around $\vec{\Gamma}$ are non linear, this time we have not stopped the expansion to the first-order terms, but we have kept also its second-order terms. The dependence on $\vec{k}$ appears in the elements of $\tilde{H}_o(\vec{k})$ (see Eq. (S44)) only through the complex exponentials $e^{i\vec{k}\cdot\vec{d}_i}$; therefore, the second-order Taylor expansion $\tilde{H}_o^{(\exp)}(\vec{\kappa})$ of the Hamiltonian $\tilde{H}_o(\vec{k})$ can be simply performed substituting in all the elements of the matrix (see Eq. (S44)) to the exponentials $e^{i\vec{k}\cdot\vec{d}_i}$ the following quantities:

$$\begin{aligned}
e^{i\vec{k}\cdot\vec{d}_1} &= e^{i\vec{\Gamma}\cdot\vec{d}_1} e^{i\vec{\kappa}\cdot\vec{d}_1} = e^{i\vec{\kappa}\cdot\vec{d}_1} \approx 1 + i\vec{\kappa}\cdot\vec{d}_1 + \frac{(i\vec{\kappa}\cdot\vec{d}_1)^2}{2} = \left[1 - \frac{1}{2}\left(\frac{a}{2}\kappa_x + \frac{a}{2\sqrt{3}}\kappa_y\right)^2\right] + i\left[\frac{a}{2}\kappa_x + \frac{a}{2\sqrt{3}}\kappa_y\right], \\
e^{i\vec{k}\cdot\vec{d}_2} &= e^{i\vec{\Gamma}\cdot\vec{d}_2} e^{i\vec{\kappa}\cdot\vec{d}_2} = e^{i\vec{\kappa}\cdot\vec{d}_2} \approx 1 + i\vec{\kappa}\cdot\vec{d}_2 + \frac{(i\vec{\kappa}\cdot\vec{d}_2)^2}{2} = \left[1 - \frac{1}{2}\left(-\frac{a}{2}\kappa_x + \frac{a}{2\sqrt{3}}\kappa_y\right)^2\right] + i\left[-\frac{a}{2}\kappa_x + \frac{a}{2\sqrt{3}}\kappa_y\right], \\
e^{i\vec{k}\cdot\vec{d}_3} &= e^{i\vec{\Gamma}\cdot\vec{d}_3} e^{i\vec{\kappa}\cdot\vec{d}_3} = e^{i\vec{\kappa}\cdot\vec{d}_3} \approx 1 + i\vec{\kappa}\cdot\vec{d}_3 + \frac{(i\vec{\kappa}\cdot\vec{d}_3)^2}{2} = \left[1 - \frac{a^2}{6}\kappa_y^2\right] - i\frac{a}{\sqrt{3}}\kappa_y
\end{aligned} \tag{S88}$$

(where we have substituted the coordinates of $\vec{\Gamma}$, $\vec{d}_1$, $\vec{d}_2$, and $\vec{d}_3$). In order to project the expanded Hamiltonian $\tilde{H}_o^{(\exp)}(\vec{\kappa})$ onto the subset of states corresponding to the bands nearest to the Fermi energy, we have first changed the basis from $\mathcal{V}_1$ to $\mathcal{M}_4$ (the basis set which diagonalizes the tight-binding Hamiltonian exactly in $\vec{\Gamma}$, i.e. for $\vec{\kappa} = 0$), obtaining the matrix $H_0'^{(\exp)}(\vec{\kappa}) = (\tilde{Q})^\dagger \tilde{H}_0^{(\exp)}(\vec{\kappa})\,\tilde{Q}$. Then, the projection of the Hamiltonian onto a subset of the overall basis $\mathcal{M}_4$ is directly obtained considering the submatrix of $H_0'^{(\exp)}(\vec{\kappa})$ which corresponds to the basis elements we are interested in. We have first tried to separately project the Hamiltonian onto the two subset of states $\{|\tau_7\rangle, |\tau_8\rangle\}$ (which are degenerate in $\vec{\Gamma}$) and $\{|\tau_3\rangle\}$ (these states would correspond to the highest valence bands and to the lowest conduction band, respectively). However, computing the eigenvalues of the two Hamiltonians that we have obtained in this way, we have found energy dispersion relations that completely miss the main features (including the sign of

the concavity) of the tight-binding energy bands. We have seen that, in order to obtain an accurate approximation, it is necessary to include in our calculation also the interaction between these bands, and thus at least consider the basis set $\{|\tau_3\rangle, |\tau_7\rangle, |\tau_8\rangle\}$. Therefore, we have projected the Hamiltonian $H_0'^{(\exp)}(\vec{\kappa})$ onto the subset of basis states $\{|\tau_3\rangle, |\tau_7\rangle, |\tau_8\rangle\}$, obtaining (if the spin is not included) a 3-band $\vec{k}\cdot\vec{p}$ description of stanene around the $\vec{\Gamma}$ point (in addition, in the Supplementary Information we report the extended $\vec{k}\cdot\vec{p}$ Hamiltonian obtained including in the basis set also the state $|\tau_1\rangle$, which corresponds to the second lowest conduction band in $\vec{\Gamma}$). Since we have previously ordered the basis elements of $\mathcal{M}_4$ in such a way as to have these three states in the first positions, the projection of $H_0'^{(\exp)}(\vec{\kappa})$ onto the set $\{|\tau_3\rangle, |\tau_7\rangle, |\tau_8\rangle\}$ is the 3×3 submatrix obtained from the intersection of its first 3 rows and of its first 3 columns. At the end of this analytical procedure, we obtain the following 3×3 matrix:

$$H_{\vec{\Gamma}}(\vec{\kappa}) = \begin{bmatrix} \epsilon_3' + c_3(\kappa_x^2+\kappa_y^2) & -ic_4\kappa_x & -ic_4\kappa_y \\ ic_4\kappa_x & -V_6 + c_5\kappa_x^2 + c_6\kappa_y^2 & -c_7\kappa_x\kappa_y \\ ic_4\kappa_y & -c_7\kappa_x\kappa_y & -V_6 + c_6\kappa_x^2 + c_5\kappa_y^2 \end{bmatrix} \tag{S89}$$

where

$$\begin{gathered} c_3 = \frac{a^2}{12}(V_4\, q_{33}^2 - V_7\, q_{43}^2 - V_5\, 2\, q_{33}\, q_{43})\,, \quad c_4 = \frac{a}{\sqrt{6}}(V_2\, q_{33} - V_3\, q_{43})\,, \\ c_5 = \frac{a^2}{8}(V_6 - V_{pp\pi})\,, \quad c_6 = \frac{a^2}{24}(V_6 + 3\ V_{pp\pi}), \quad c_7 = \frac{a^2}{12}V_1\,. \end{gathered} \tag{S90}$$

Now let us include also the effect of the spin-orbit coupling (neglecting the Rashba term). Rearranging Eq. (8), the spin-orbit Hamiltonian on the basis of 8 atomic orbitals (differing also for the spin) centered on the same atom $\{|s\uparrow\rangle, |s\downarrow\rangle, |p_x\uparrow\rangle, |p_x\downarrow\rangle, |p_y\uparrow\rangle, |p_y\downarrow\rangle, |p_z\uparrow\rangle, |p_z\downarrow\rangle$ is:

$$\hat{H}_{SO} = \frac{\Delta_{SO}}{3} \begin{bmatrix} 0&0&0&0&0&0&0&0 \\ 0&0&0&0&0&0&0&0 \\ 0&0&0&0&-i&0&0&1 \\ 0&0&0&0&0&i&-1&0 \\ 0&0&i&0&0&0&0&-i \\ 0&0&0&-i&0&0&-i&0 \\ 0&0&0&-1&0&i&0&0 \\ 0&0&1&0&i&0&0&0 \end{bmatrix}. \tag{S91}$$

Since no spin-orbit interaction exists between orbitals on different atoms, on the basis set $\mathcal{V}_1\otimes\{|\uparrow\rangle, |\downarrow\rangle\} = \{|s^A\uparrow\rangle, |s^A\downarrow\rangle, |p_x^A\uparrow\rangle, |p_x^A\downarrow\rangle, |p_y^A\uparrow\rangle, |p_y^A\downarrow\rangle, |p_z^A\uparrow\rangle, |p_z^A\downarrow\rangle, |s^B\uparrow\rangle, |s^B\downarrow\rangle, |p_x^B\uparrow\rangle, |p_x^B\downarrow\rangle, |p_y^B\uparrow\rangle, |p_y^B\downarrow\rangle, |p_z^B\uparrow\rangle, |p_z^B\downarrow\rangle\}$ (the operator $\otimes$ being the tensor product) the spin-orbit contribution to the Hamiltonian is

$$\tilde{H}_{SO} = \begin{bmatrix} \hat{H}_{SO} & 0 \\ 0 & \hat{H}_{SO} \end{bmatrix}. \tag{S92}$$

On the other hand, the part of Hamiltonian which does not include the spin-orbit interaction, i.e. $\tilde{H}_0(\vec{k})$, acts identically on spin-up and spin-down orbitals, and thus it can be expressed on the basis set $\mathcal{V}_1\otimes\{|\uparrow\rangle, |\downarrow\rangle\}$ as $\tilde{H}_0(\vec{k})\otimes I_2$ (where I_2 is the 2×2 identity matrix). Therefore, the total Hamiltonian on the basis set $\mathcal{V}_1\otimes\{|\uparrow\rangle, |\downarrow\rangle\}$ is $\tilde{H}_0(\vec{k})\otimes I_2 + \tilde{H}_{SO}$. We can repeat our previous treatment on this extended Hamiltonian. We first perform a second-order Taylor expansion around $\vec{\Gamma}$ (only of the first term, because the second term does not depend on $\vec{k}$). Then we move from the basis set $\mathcal{V}_1\otimes\{|\uparrow\rangle, |\downarrow\rangle\}$ to the basis set $\mathcal{M}_4\otimes\{|\uparrow\rangle, |\downarrow\rangle\}$ through the matrix $\tilde{Q}\otimes I_2$, obtaining:

$$\begin{aligned} &(\tilde{Q}\otimes I_2)^\dagger(\tilde{H}_0^{(\exp)}(\vec{\kappa})\otimes I_2 + \tilde{H}_{SO})(\tilde{Q}\otimes I_2) = \\ &= (\tilde{Q}\otimes I_2)^\dagger(\tilde{H}_0^{(\exp)}(\vec{\kappa})\otimes I_2)(\tilde{Q}\otimes I_2) + (\tilde{Q}\otimes I_2)^\dagger\tilde{H}_{SO}(\tilde{Q}\otimes I_2) = \\ &= H_0'^{(\exp)}(\vec{\kappa})\otimes I_2 + (\tilde{Q}\otimes I_2)^\dagger\tilde{H}_{SO}(\tilde{Q}\otimes I_2)\,. \end{aligned} \tag{S93}$$

Finally, we project the resulting Hamiltonian onto the basis $\{\,|\tau_3\rangle,\ |\tau_7\rangle,\ |\tau_8\rangle\}\otimes\{\,|\uparrow\rangle,\ |\downarrow\rangle\,\} = \{\,|\tau_3\uparrow\rangle,\ |\tau_3\downarrow\rangle,\ |\tau_7\uparrow$

$\rangle$, $|\tau_7\downarrow\rangle$, $|\tau_8\uparrow\rangle$, $|\tau_8\downarrow\rangle$ }, obtaining:

$$H_{\vec{\Gamma}}(\vec{\kappa}) \otimes I_2 + \frac{\Delta_{SO}}{3} \begin{bmatrix} 0 & 0 & 0 & 0 & 0 & 0 \\ 0 & 0 & 0 & 0 & 0 & 0 \\ 0 & 0 & 0 & 0 & -i & 0 \\ 0 & 0 & 0 & 0 & 0 & i \\ 0 & 0 & i & 0 & 0 & 0 \\ 0 & 0 & 0 & -i & 0 & 0 \end{bmatrix}, \quad \text{(S94)}$$

where $H_{\vec{\Gamma}}(\vec{\kappa})$ is given by Eq. (S89). The second term of Eq. (S94) is obtained performing the product $(\tilde{Q}\otimes I_2)^\dagger \tilde{H}_{SO}(\tilde{Q}\otimes I_2)$ and considering its 6×6 submatrix given by the intersection of its first 6 rows and of its first 6 columns (since $\{\,|\tau_3\uparrow\rangle, |\tau_3\downarrow\rangle, |\tau_7\uparrow\rangle, |\tau_7\downarrow\rangle, |\tau_8\uparrow\rangle, |\tau_8\downarrow\rangle\,\}$ represent the first 6 elements of the basis set $\mathcal{M}_4\otimes\{|\uparrow\rangle, |\downarrow\rangle\}$).

Following this procedure, we have neglected the coupling which exists between the 6 states on which we have performed our projection and the other 10 states that we have discarded (as we have seen, only the Hamiltonian without spin-orbit in $\vec{k}=\vec{\Gamma}$ is diagonal on the basis $\mathcal{M}_4\otimes\{|\uparrow\rangle, |\downarrow\rangle\}$). The effect of this interaction can be partially recovered adopting the method described in the Appendix B of Ref. [1]. We can rewrite the matrix $H_0'^{(\exp)}(\vec{\kappa})\otimes I_2 + (\tilde{Q}\otimes I_2)^\dagger \tilde{H}_{SO}(\tilde{Q}\otimes I_2)$ of Eq. (S93) (where the first term is the Hamiltonian without spin-orbit coupling and the second term is the spin-orbit contribution) in the form

$$\begin{bmatrix} H_\pi & H_n \\ H_n^\dagger & H_\sigma \end{bmatrix}, \quad \text{(S95)}$$

where the 6×6 matrix H_π is given by the intersection of the first 6 rows and of the first 6 columns (i.e., the rows and the columns corresponding to the states considered in our projection) of the Hamiltonian of Eq. (S93), the 6×10 matrix H_n is given by the intersection of its first 6 rows and of its 7th-to-16th columns, the 10×6 matrix $H_n^\dagger$ is given by the intersection of its 7th-to-16th rows and of its first 6 columns, and the 10×10 matrix H_σ is given by the intersection of its 7th-to-16th rows and of its 7th-to-16th columns. Let us define ϵ_{av} the average of the eigenvalues of $H_{\vec{\Gamma}}(\vec{0})$, i.e. $\epsilon_{av} = (\epsilon_3' - V_6 - V_6)/3$. Since the eigenvalues of H_π are near ϵ_{av}, the eigenvalues of H_σ are far from it, and H_n is much smaller than $H_\sigma - \epsilon_{av}\, I_{10}$, following the procedure described in the Appendix B of Ref. [1] we can approximate the Hamiltonian of Eq. (S93) near ϵ_{av} with $H_\pi - H_n(H_\sigma - \epsilon_{av}\, I_{10})^{-1}H_n^\dagger$ (where I_{10} is the 10×10 identity matrix). The matrix H_π represents the projection of Eq. (S93) on the first 6 states of the basis $\mathcal{M}_4\otimes\{|\uparrow\rangle, |\downarrow\rangle\}$, i.e. exactly Eq. (S94). Instead, the term $-H_n(H_\sigma - \epsilon_{av}\, I_{10})^{-1}H_n^\dagger$ gives rise to an effective second-order spin-orbit contribution. Let us approximate [1] the Hamiltonian of Eq. (S93) with its value in $\vec{\kappa}=0$ (i.e. in $\vec{k}=\vec{\Gamma}$); in this case its part without spin-orbit is the diagonal matrix $H_0'(\vec{\Gamma})\otimes I_2$. Therefore, the matrix H_n derives only from the spin-orbit part of Eq. (S93) and is given by the intersection of the first 6 rows and of the 7th-to-16th columns of $(\tilde{Q}\otimes I_2)^\dagger \tilde{H}_{SO}(\tilde{Q}\otimes I_2)$. Instead, in H_σ the contribution of the Hamiltonian without spin-orbit dominates on that of spin-orbit and we can approximate H_σ with the intersection of the 7th-to-16th rows and of the 7th-to-16th columns of $H_0'(\vec{\Gamma})\otimes I_2$. Performing the calculation, we obtain the following quantity:

$$H_{SO}^{2nd} = \begin{bmatrix} e_1 & 0 & 0 & 0 & 0 & 0 \\ 0 & e_1 & 0 & 0 & 0 & 0 \\ 0 & 0 & e_2 & 0 & ie_2 & 0 \\ 0 & 0 & 0 & e_2 & 0 & -ie_2 \\ 0 & 0 & -ie_2 & 0 & e_2 & 0 \\ 0 & 0 & 0 & ie_2 & 0 & e_2 \end{bmatrix}, \quad \text{(S96)}$$

with

$$e_1 = \left(\frac{\Delta_{SO}}{3}\right)^2 \frac{2\,q_{43}^2}{\epsilon_{av} - V_6}, \quad e_2 = \left(\frac{\Delta_{SO}}{3}\right)^2 \left(\frac{q_{21}^2}{\epsilon_{av} - \epsilon_1'} + \frac{q_{22}^2}{\epsilon_{av} - \epsilon_2'}\right), \quad \epsilon_{av} = \frac{\epsilon_3' - V_6 - V_6}{3}. \quad \text{(S97)}$$

Adding Eq. (S96) to Eq. (S94), we obtain the Hamiltonian

$$H_{\vec{\Gamma}}(\vec{\kappa}) \otimes I_2 + \begin{bmatrix} f_1 & 0 & 0 & 0 & 0 & 0 \\ 0 & f_1 & 0 & 0 & 0 & 0 \\ 0 & 0 & f_2 & 0 & -if_3 & 0 \\ 0 & 0 & 0 & f_2 & 0 & if_3 \\ 0 & 0 & if_3 & 0 & f_2 & 0 \\ 0 & 0 & 0 & -if_3 & 0 & f_2 \end{bmatrix}, \quad \text{(S98)}$$

where

$$f_1 = e_1\,,\quad f_2 = e_2\,,\quad f_3 = \frac{\Delta_{SO}}{3} - e_2\,. \tag{S99}$$

The $\vec{k}\cdot\vec{p}$ parameters obtained from the tight-binding ones (in particular, the NNTB parameters of Table 1) are reported in Table 2. In order to compute these values, we have exploited the Eqs. (S49), (S53), (S77), (S78), (S79), (S81), (S82), (S83), (S72), (S90), (S57), (S59), (S61), (S97), and (S99). Moreover, remembering that in Eq. (S43) we have subtracted $\epsilon_p = 1.7747$ eV to all the elements on the diagonal and that this shift survives all the basis changes we have previously performed, now we have added back $\epsilon_p = 1.7747$ eV to the elements located on the diagonal of the $\vec{k}\cdot\vec{p}$ Hamiltonians. This means that we have added $\epsilon_p = 1.7747$ eV to ϵ_1 and ϵ_3', while we have subtracted $\epsilon_p = 1.7747$ eV to V_6 (because $-V_6$ appears on the diagonal of Eq. (S89)).

The $\vec{k}\cdot\vec{p}$ dispersion relations are obtained finding the eigenvalues of the $\vec{k}\cdot\vec{p}$ Hamiltonians (Eqs. (S52), (S60), (S65), (S70), (S89), (S98)). In Fig. 6 we show the comparison between the tight-binding bands and the $\vec{k}\cdot\vec{p}$ dispersion relations computed around the $\vec{K}$ and Γ points, both neglecting and including the spin-orbit coupling effect in the calculation.

B.4: Fitting of the $\vec{k}\cdot\vec{p}$ parameters

With the previously described procedure we have been able to find the expressions of the $\vec{k}\cdot\vec{p}$ Hamiltonians around the points $\vec{K}'$, $\vec{K}$ and Γ (Eqs. (S52), (S60), (S65), (S70), (S89), (S98)) and to express the $\vec{k}\cdot\vec{p}$ parameters which appear inside them as a function of the nearest-neighbor tight-binding parameters (Eqs. (S49), (S53), (S77), (S78), (S79), (S81), (S82), (S83), (S72), (S90), (S57), (S59), (S61), (S97), (S99)). However, these tight-binding parameters have been found fitting the overall set of DFT bands along the maximum symmetry directions (the closed loop of straight lines joining $\vec{M}$, Γ, and $\vec{K}$) of the entire Brillouin zone (even though weighting more the regions nearest to the Fermi energy). Once we have the analytical form of the $\vec{k}\cdot\vec{p}$ dispersion relations, it is more useful to obtain the $\vec{k}\cdot\vec{p}$ parameters (ϵ_1, γ, ϵ_3', V_6, c_3, c_4, c_5, c_6, c_7, d_1, d_2, f_1, f_2, f_3) directly fitting the DFT dispersion relations in the regions around the points $\vec{K}$ (or $\vec{K}'$) and Γ, in such a way as to improve their local accuracy. In detail, we have chosen the $\vec{k}\cdot\vec{p}$ parameters in such a way as to minimize the following error function:

$$\chi = \sum_{\vec{P}=\{\vec{K},\Gamma\}} \sum_{\substack{\Sigma=\\ \{\text{without so},\\ \text{with so}\}}} \sum_{b=1}^{N_b(\vec{P},\Sigma)} \sum_{\vec{k}\in I(\vec{P})} w(\vec{k},\vec{P})\,(E_{b,\Sigma}^{\vec{k}\cdot\vec{p}}(\vec{k}) - E_{b,\Sigma}^{\mathrm{DFT}}(\vec{k}))^2\,, \tag{S100}$$

where $E_{b,\Sigma}^{\vec{k}\cdot\vec{p}}(\vec{k})$ and $E_{b,\Sigma}^{\mathrm{DFT}}(\vec{k})$ are the $\vec{k}\cdot\vec{p}$ and DFT dispersion relation, respectively, $I(\vec{P})$ is the subset of the values of $\vec{k}$ along the maximum symmetry directions (for which the DFT bands have been computed) which are nearer to the points $\vec{K}$ and Γ than $\pi/(6\,a_0)$ (i.e. 1/8 of the distance between Γ and $\vec{K}$), and

$$w(\vec{k},\vec{P}) = \frac{1}{1+10^4\ |\vec{k}-\vec{P}|^2} \tag{S101}$$

(in such a way as to attribute a greater weight to the energy values corresponding to the $\vec{k}$'s nearest to $\vec{K}$ and Γ). For each of the considered $\vec{k}$'s, the calculation of χ is performed on all the bands (specified by the index b), without and with spin-orbit interaction, for which we have found the $\vec{k}\cdot\vec{p}$ expressions: the number N_b of these bands near $\vec{K}$ is 2 without spin-orbit and 4 with spin-orbit, while near Γ it is 3 without spin-orbit and 6 with spin-orbit. We notice that in the actual implementation of the fitting procedure we have included also a further fitting parameter: the relative energy shift which in practice exists between the DFT dispersion relations obtained neglecting the spin-orbit contribution and those computed including it. The minimization has been performed using the direct search method described in Ref. [6].

The values of the $\vec{k}\cdot\vec{p}$ parameters that we have obtained at the end of this procedure are reported in Table 3.

In Fig. 7 we show the comparison between the DFT bands and the $\vec{k}\cdot\vec{p}$ dispersion relations obtained, using these values for the $\vec{k}\cdot\vec{p}$ parameters, around the $\vec{K}$ and Γ points, both neglecting and including the spin-orbit coupling effect in the calculation.

If we are interested in the dispersion relations in the presence of a magnetic field $\vec{B}$, we can simply substitute the coordinates κ_ℓ (with $\ell = x, y$) with $\kappa_\ell + (e/\hbar)A_\ell$ inside our $\vec{k} \cdot \vec{p}$ Hamiltonians (Eqs. (S52), (S60), (S65), (S70), (S89), (S98)). Here, e is the modulus of the electron charge, $\hbar$ is the reduced Planck constant, and A_ℓ is the ℓ-th coordinate of the vector potential $\vec{A}$ (chosen in such a way that $\vec{B} = \vec{\nabla} \times \vec{A}$).

REFERENCES

[1] C.-C. Liu, H. Jiang, and Y. Yao. Low-energy effective Hamiltonian involving spin-orbit coupling in silicene and two-dimensional germanium and tin. *Phys. Rev. B*, 84(19):195430, 2011.

[2] L. C. Lew Yan Voon, A. Lopez-Bezanilla, J. Wang, Y. Zhang, and M. Willatzen. Effective Hamiltonians for phosphorene and silicene. *New J. Phys.*, 17(2):025004, 2015.

[3] F. Geissler, J. C. Budich, and B. Trauzettel. Group theoretical and topological analysis of the quantum spin Hall effect in silicene. *New J. Phys.*, 15(8):085030, 2013.

[4] M. Ezawa. Spin valleytronics in silicene: Quantum spin Hall: Quantum anomalous Hall insulators and single-valley semimetals. *Phys. Rev. B*, 87(15):155415, 2013.

[5] C.-C. Liu, W. Feng, and Y. Yao. Quantum spin Hall effect in silicene and two-dimensional germanium. *Phys. Rev. Lett.*, 107(7):076802, 2011.

[6] J. P. Chandler. STEPIT: Finds local minima of a smooth function of several parameters (CPA 312). *Behav. Sci.*, 14:81–82, 1969.
