# Supplementary material for: A tight binding and k → ⋅ p → study of monolayer stanene
Source: Sci Rep. 2017 Sep 21;7:12069. doi: 10.1038/s41598-017-12281-y (PMC5608720; doi:10.1038/s41598-017-12281-y)
Supplement: Supplementary file 2 — SUPPLEMENTARY MATERIAL [file 41598_2017_12281_MOESM2_ESM.pdf]

SUPPLEMENTARY INFORMATION

for the paper:

Liming Jiang, Paolo Marconcini, Md Sharafat Hossian,
Wanzhi Qiu, Robin Evans, Massimo Macucci, Efstratios Skafidas,

“A TIGHT BINDING AND $\vec{k} \cdot \vec{p}$ STUDY OF MONOLAYER STANENE”

I. $\vec{K} \cdot \vec{P}$ ANALYSIS AT THE POINT $\vec{K}'$

In the following, we will strictly follow the procedure of Ref. [1]. First, let us diagonalize the tight-binding Hamiltonian exactly at the point $\vec{K}' = [4\pi/(3a_0),\, 0,\, 0]^T$. In $\vec{K}'$ we have that $e^{i\vec{K}'\cdot\vec{d}_1} = e^{i2\pi/3}$, $e^{i\vec{K}'\cdot\vec{d}_2} = e^{-i2\pi/3}$, $e^{i\vec{K}'\cdot\vec{d}_3} = 1$, and therefore the Hamiltonian (S43) becomes:

$$\tilde{H}_0(\vec{K}') = \begin{bmatrix} \Delta & 0 & 0 & 0 & 0 & iV_2' & -V_2' & 0 \\ 0 & 0 & 0 & 0 & -iV_2' & V_1' & -iV_1' & -iV_3' \\ 0 & 0 & 0 & 0 & V_2' & -iV_1' & -V_1' & V_3' \\ 0 & 0 & 0 & \Delta_{pz} & 0 & -iV_3' & V_3' & 0 \\ 0 & iV_2' & V_2' & 0 & \Delta & 0 & 0 & 0 \\ -iV_2' & V_1' & iV_1' & iV_3' & 0 & 0 & 0 & 0 \\ -V_2' & iV_1' & -V_1' & V_3' & 0 & 0 & 0 & 0 \\ 0 & iV_3' & V_3' & 0 & 0 & 0 & 0 & \Delta_{pz} \end{bmatrix} \tag{S102}$$

with

$$V_1' = \frac{3}{4}\sin^2\theta\,(V_{pp\pi} - V_{pp\sigma})\,, \quad V_2' = \frac{3}{2}\sin\theta\, V_{sp\sigma}\,, \quad V_3' = \frac{3}{2}\sin\theta\,\cos\theta\,(V_{pp\pi} - V_{pp\sigma})\,. \tag{S103}$$

Exactly as in Ref. [1], let us move from the basis set $\mathcal{V}_1$ to the basis set $\mathcal{V}_2 = \{|p_z^A\rangle, |s^A\rangle, |\varphi_2^B\rangle, |p_z^B\rangle, |s^B\rangle, |\varphi_1^A\rangle, |\varphi_3\rangle, |\varphi_4\rangle)$, where

$$\begin{gathered} |\varphi_1^A\rangle = -\frac{1}{\sqrt{2}}(|p_x^A\rangle + i\,|p_y^A\rangle)\,, \quad |\varphi_2^B\rangle = \frac{1}{\sqrt{2}}(|p_x^B\rangle - i\,|p_y^B\rangle)\,, \\ |\varphi_3\rangle = -\frac{1}{2}(|p_x^A\rangle - i\,|p_y^A\rangle) - \frac{1}{2}(|p_x^B\rangle + i\,|p_y^B\rangle)\,, \quad |\varphi_4\rangle = \frac{1}{2}(|p_x^A\rangle - i\,|p_y^A\rangle) - \frac{1}{2}(|p_x^B\rangle + i\,|p_y^B\rangle) \end{gathered} \tag{S104}$$

through the unitary transformation

$$\tilde{U}_1 = \begin{bmatrix} 0 & 1 & 0 & 0 & 0 & 0 & 0 & 0 \\ 0 & 0 & 0 & 0 & 0 & -\frac{1}{\sqrt{2}} & -\frac{1}{2} & \frac{1}{2} \\ 0 & 0 & 0 & 0 & 0 & -\frac{i}{\sqrt{2}} & \frac{i}{2} & -\frac{i}{2} \\ 1 & 0 & 0 & 0 & 0 & 0 & 0 & 0 \\ 0 & 0 & 0 & 0 & 1 & 0 & 0 & 0 \\ 0 & 0 & \frac{1}{\sqrt{2}} & 0 & 0 & 0 & -\frac{1}{2} & -\frac{1}{2} \\ 0 & 0 & -\frac{i}{\sqrt{2}} & 0 & 0 & 0 & -\frac{i}{2} & -\frac{i}{2} \\ 0 & 0 & 0 & 1 & 0 & 0 & 0 & 0 \end{bmatrix}. \tag{S105}$$

The Hamiltonian becomes

$$H_1(\vec{K}') = (\tilde{U}_1)^\dagger \, \tilde{H}_0(\vec{K}') \, \tilde{U}_1 = \begin{bmatrix} \Delta_{pz} & 0 & -iV_3 & 0 & 0 & 0 & 0 & 0 \\ 0 & \Delta & iV_2 & 0 & 0 & 0 & 0 & 0 \\ iV_3 & -iV_2 & 0 & 0 & 0 & 0 & 0 & 0 \\ 0 & 0 & 0 & \Delta_{pz} & 0 & -iV_3 & 0 & 0 \\ 0 & 0 & 0 & 0 & \Delta & -iV_2 & 0 & 0 \\ 0 & 0 & 0 & iV_3 & iV_2 & 0 & 0 & 0 \\ 0 & 0 & 0 & 0 & 0 & 0 & V_1 & 0 \\ 0 & 0 & 0 & 0 & 0 & 0 & 0 & -V_1 \end{bmatrix} , \tag{S106}$$

with $V_1 = 2V_1'$, $V_2 = \sqrt{2}V_2'$, and $V_3 = \sqrt{2}V_3'$. This Hamiltonian is a block diagonal matrix with the following blocks on the diagonal:

$$H_A^{\vec{K}'} = \begin{bmatrix} \Delta_{pz} & 0 & -iV_3 \\ 0 & \Delta & iV_2 \\ iV_3 & -iV_2 & 0 \end{bmatrix} , \quad H_B^{\vec{K}'} = \begin{bmatrix} \Delta_{pz} & 0 & -iV_3 \\ 0 & \Delta & -iV_2 \\ iV_3 & iV_2 & 0 \end{bmatrix} , \quad V_1 \, , \quad -V_1 \, . \tag{S107}$$

The eigenvalues ϵ_i (with $i = 1, 2, 3$, where $\epsilon_3 < \epsilon_1 < \epsilon_2$) of $H_A^{\vec{K}'}$ are the roots of the equation $\det(H_A^{\vec{K}'} - \epsilon I_3) = 0$ (where I_3 is the 3×3 identity matrix), i.e. of

$$\epsilon^3 - (\Delta + \Delta_{pz})\epsilon^2 - (-\Delta_{pz}\Delta + V_2^2 + V_3^2)\epsilon + \Delta_{pz}V_2^2 + \Delta V_3^2 = 0 \, , \tag{S108}$$

while the corresponding orthonormalized eigenvectors are

$$\begin{bmatrix} \dfrac{1}{\alpha_i} \\ \dfrac{V_2(\epsilon_i - \Delta_{pz})}{\alpha_i(\Delta - \epsilon_i)V_3} \\ \dfrac{i(\epsilon_i - \Delta_{pz})}{\alpha_i V_3} \end{bmatrix} \equiv \begin{bmatrix} u_{1i} \\ u_{2i} \\ u_{3i} \end{bmatrix} \tag{S109}$$

where

$$\alpha_i = \sqrt{1 + \left(\frac{V_2(\epsilon_i - \Delta_{pz})}{(\Delta - \epsilon_i)V_3} \right)^2 + \left(\frac{\epsilon_i - \Delta_{pz}}{V_3} \right)^2} \, . \tag{S110}$$

The matrix of the eigenvectors U_A diagonalizes $H_A^{\vec{K}'}$, i.e. $U_A^\dagger H_A^{\vec{K}'} U_A = \Lambda_A$, with

$$U_A = \begin{bmatrix} u_{11} & u_{12} & u_{13} \\ u_{21} & u_{22} & u_{23} \\ u_{31} & u_{32} & u_{33} \end{bmatrix} , \quad \Lambda_A = \begin{bmatrix} \epsilon_1 & 0 & 0 \\ 0 & \epsilon_2 & 0 \\ 0 & 0 & \epsilon_3 \end{bmatrix} . \tag{S111}$$

The eigenvalues of $H_B^{\vec{K}'}$ are again the roots of Eq. (S108) and thus coincide with those of $H_A^{\vec{K}'}$, but its eigenvectors have the form $[\, u_{1i}, \, -u_{2i}, \, u_{3i} \,]^T$. Therefore, we have that $U_B^\dagger H_B^{\vec{K}'} U_B = \Lambda_B$, with

$$U_B = \begin{bmatrix} u_{11} & u_{12} & u_{13} \\ -u_{21} & -u_{22} & -u_{23} \\ u_{31} & u_{32} & u_{33} \end{bmatrix} , \quad \Lambda_B = \Lambda_A \, . \tag{S112}$$

From this it follows that $(U_2')^\dagger H_1(\vec{K}')U_2' = H_2(\vec{K}')$, where

$$U_2' = \begin{bmatrix} u_{11} & u_{12} & u_{13} & 0 & 0 & 0 & 0 & 0 \\ u_{21} & u_{22} & u_{23} & 0 & 0 & 0 & 0 & 0 \\ u_{31} & u_{32} & u_{33} & 0 & 0 & 0 & 0 & 0 \\ 0 & 0 & 0 & u_{11} & u_{12} & u_{13} & 0 & 0 \\ 0 & 0 & 0 & -u_{21} & -u_{22} & -u_{23} & 0 & 0 \\ 0 & 0 & 0 & u_{31} & u_{32} & u_{33} & 0 & 0 \\ 0 & 0 & 0 & 0 & 0 & 0 & 1 & 0 \\ 0 & 0 & 0 & 0 & 0 & 0 & 0 & 1 \end{bmatrix}, \quad H_2(\vec{K}') = \begin{bmatrix} \epsilon_1 & 0 & 0 & 0 & 0 & 0 & 0 & 0 \\ 0 & \epsilon_2 & 0 & 0 & 0 & 0 & 0 & 0 \\ 0 & 0 & \epsilon_3 & 0 & 0 & 0 & 0 & 0 \\ 0 & 0 & 0 & \epsilon_1 & 0 & 0 & 0 & 0 \\ 0 & 0 & 0 & 0 & \epsilon_2 & 0 & 0 & 0 \\ 0 & 0 & 0 & 0 & 0 & \epsilon_3 & 0 & 0 \\ 0 & 0 & 0 & 0 & 0 & 0 & V_1 & 0 \\ 0 & 0 & 0 & 0 & 0 & 0 & 0 & -V_1 \end{bmatrix}. \tag{S113}$$

In this way, we have moved from the basis set $\mathcal{V}_2$ to the basis set $\mathcal{V}_3 = \{|\Phi_1\rangle, |\Phi_2\rangle, |\Phi_3\rangle, |\Phi_4\rangle, |\Phi_5\rangle, |\Phi_6\rangle, |\Phi_7\rangle, |\Phi_8\rangle\}$, where $|\Phi_1\rangle = u_{11}|p_z^A\rangle + u_{21}|s^A\rangle + u_{31}|\varphi_2^B\rangle$, $|\Phi_2\rangle = u_{12}|p_z^A\rangle + u_{22}|s^A\rangle + u_{32}|\varphi_2^B\rangle$, $|\Phi_3\rangle = u_{13}|p_z^A\rangle + u_{23}|s^A\rangle + u_{33}|\varphi_2^B\rangle$, $|\Phi_4\rangle = u_{11}|p_z^B\rangle - u_{21}|s^B\rangle + u_{31}|\varphi_1^A\rangle$, $|\Phi_5\rangle = u_{12}|p_z^B\rangle - u_{22}|s^B\rangle + u_{32}|\varphi_1^A\rangle$, $|\Phi_6\rangle = u_{13}|p_z^B\rangle - u_{23}|s^B\rangle + u_{33}|\varphi_1^A\rangle$, $|\Phi_7\rangle = |\varphi_3\rangle$, and $|\Phi_8\rangle = |\varphi_4\rangle$.

Substituting the values of the NNTB parameters of Table 1 into the expressions of V_1, V_2 and V_3 and solving Eq. (S108), we obtain that $\epsilon_3 = -9.8374$ eV (double degenerate), $V_1 = -4.1959$ eV, $\epsilon_1 = -1.7430$ eV (double degenerate), $\epsilon_2 = 2.4555$ eV (double degenerate), $-V_1 = 4.1959$ eV. Therefore, the Fermi energy ϵ_F (which leaves half of the energy levels occupied, i.e. with values lower than ϵ_F, and half unoccupied, i.e. with values higher than ϵ_F) is located at ϵ_1 and thus in our analysis around $\vec{K}'$ we have focused on the states corresponding to this eigenvalue.

Following Ref. [1], we have permuted the position of the basis states in such a way as to group the states with the same eigenvectors through a final change of basis $(U_2'')^\dagger H_2(\vec{K}')U_2'' = H_0'(\vec{K}')$, where

$$U_2'' = \begin{bmatrix} 1 & 0 & 0 & 0 & 0 & 0 & 0 & 0 \\ 0 & 0 & 1 & 0 & 0 & 0 & 0 & 0 \\ 0 & 0 & 0 & 0 & 1 & 0 & 0 & 0 \\ 0 & 1 & 0 & 0 & 0 & 0 & 0 & 0 \\ 0 & 0 & 0 & 1 & 0 & 0 & 0 & 0 \\ 0 & 0 & 0 & 0 & 0 & 1 & 0 & 0 \\ 0 & 0 & 0 & 0 & 0 & 0 & 1 & 0 \\ 0 & 0 & 0 & 0 & 0 & 0 & 0 & 1 \end{bmatrix}, \quad H_0'(\vec{K}') = \begin{bmatrix} \epsilon_1 & 0 & 0 & 0 & 0 & 0 & 0 & 0 \\ 0 & \epsilon_1 & 0 & 0 & 0 & 0 & 0 & 0 \\ 0 & 0 & \epsilon_2 & 0 & 0 & 0 & 0 & 0 \\ 0 & 0 & 0 & \epsilon_2 & 0 & 0 & 0 & 0 \\ 0 & 0 & 0 & 0 & \epsilon_3 & 0 & 0 & 0 \\ 0 & 0 & 0 & 0 & 0 & \epsilon_3 & 0 & 0 \\ 0 & 0 & 0 & 0 & 0 & 0 & V_1 & 0 \\ 0 & 0 & 0 & 0 & 0 & 0 & 0 & -V_1 \end{bmatrix}, \tag{S114}$$

in this way moving from the basis set $\mathcal{V}_3$ to the basis set $\mathcal{V}_4 = \{|\Phi_1\rangle, |\Phi_4\rangle, |\Phi_2\rangle, |\Phi_5\rangle, |\Phi_3\rangle, |\Phi_6\rangle, |\Phi_7\rangle, |\Phi_8\rangle\}$.

The overall unitary matrix $\tilde{U}$ corresponding to the basis change from $\mathcal{V}_1$ to $\mathcal{V}_4$, i.e. such that $(\tilde{U})^\dagger \tilde{H}_0(\vec{K}')\tilde{U} = H_0'(\vec{K}')$, is

$$\tilde{U} = \tilde{U}_1 U_2' U_2'' = \begin{bmatrix} u_{21} & 0 & u_{22} & 0 & u_{23} & 0 & 0 & 0 \\ 0 & -\frac{u_{31}}{\sqrt{2}} & 0 & -\frac{u_{32}}{\sqrt{2}} & 0 & -\frac{u_{33}}{\sqrt{2}} & -\frac{1}{2} & \frac{1}{2} \\ 0 & -\frac{iu_{31}}{\sqrt{2}} & 0 & -\frac{iu_{32}}{\sqrt{2}} & 0 & -\frac{iu_{33}}{\sqrt{2}} & \frac{i}{2} & -\frac{i}{2} \\ u_{11} & 0 & u_{12} & 0 & u_{13} & 0 & 0 & 0 \\ 0 & -u_{21} & 0 & -u_{22} & 0 & -u_{23} & 0 & 0 \\ \frac{u_{31}}{\sqrt{2}} & 0 & \frac{u_{32}}{\sqrt{2}} & 0 & \frac{u_{33}}{\sqrt{2}} & 0 & -\frac{1}{2} & -\frac{1}{2} \\ -\frac{iu_{31}}{\sqrt{2}} & 0 & -\frac{iu_{32}}{\sqrt{2}} & 0 & -\frac{iu_{33}}{\sqrt{2}} & 0 & -\frac{i}{2} & -\frac{i}{2} \\ 0 & u_{11} & 0 & u_{12} & 0 & u_{13} & 0 & 0 \end{bmatrix}. \tag{S115}$$

In analogy with Ref. [1], we have obtained the $\vec{k}\cdot\vec{p}$ Hamiltonian of stanene around $\vec{K}'$ performing a low-order expansion around $\vec{K}'$ of the tight-binding Hamiltonian of Eq. (S43) and reducing the matrix size through projection onto the subset of states we are mainly interested in (i.e., those with energy nearest to the Fermi energy).

More in detail, in $\tilde{H}_o(\vec{k})$ (see Eq. (S43)) we have substituted each occurrence of $\vec{k}$ with $\vec{K}' + \vec{\kappa}$ (where $\vec{\kappa} = \vec{k} - \vec{K}'$ represents the distance in the reciprocal space between $\vec{K}'$ and $\vec{k}$); then we have performed a Taylor expansion around

$\vec{\kappa}=0$ (which corresponds to $\vec{k}=\vec{K}'$) of all the elements of the matrix. Since we are interested only in the behavior of the dispersion relations near $\vec{K}'$ (i.e. for small values of $\vec{\kappa}$) and from the DFT and tight-binding results it appears that the dispersion relations around $\vec{K}'$ are nearly linear, we have kept only the first-order terms of the expansion. The dependence on $\vec{k}$ appears in the elements of $\tilde{H}_o(\vec{k})$ (see Eq. (S44)) only through the complex exponentials $e^{i\vec{k}\cdot\vec{d}_i}$; therefore, the first-order Taylor expansion $\tilde{H}_o^{(\exp)}(\vec{\kappa})$ of the Hamiltonian $\tilde{H}_o(\vec{k})$ can be simply performed substituting the following quantities to the exponentials $e^{i\vec{k}\cdot\vec{d}_i}$ in all the elements (Eq. (S44)) of the matrix:

$$
\begin{aligned}
e^{i\vec{k}\cdot\vec{d}_1} &= e^{i\vec{K}'\cdot\vec{d}_1}e^{i\vec{\kappa}\cdot\vec{d}_1} \approx e^{i\vec{K}'\cdot\vec{d}_1}(1+i\vec{\kappa}\cdot\vec{d}_1) = \left(-\frac{1}{2}+i\frac{\sqrt{3}}{2}\right)\left(1+i\frac{a_0}{2}\kappa_x+i\frac{a_0}{2\sqrt{3}}\kappa_y\right) , \\
e^{i\vec{k}\cdot\vec{d}_2} &= e^{i\vec{K}'\cdot\vec{d}_2}e^{i\vec{\kappa}\cdot\vec{d}_2} \approx e^{i\vec{K}'\cdot\vec{d}_2}(1+i\vec{\kappa}\cdot\vec{d}_2) = \left(-\frac{1}{2}-i\frac{\sqrt{3}}{2}\right)\left(1-i\frac{a_0}{2}\kappa_x+i\frac{a_0}{2\sqrt{3}}\kappa_y\right) , \\
e^{i\vec{k}\cdot\vec{d}_3} &= e^{i\vec{K}'\cdot\vec{d}_3}e^{i\vec{\kappa}\cdot\vec{d}_3} \approx e^{i\vec{K}'\cdot\vec{d}_3}(1+i\vec{\kappa}\cdot\vec{d}_3) = 1-i\frac{a_0}{\sqrt{3}}\kappa_y
\end{aligned}
\tag{S116}
$$

(where we have substituted the coordinates of $\vec{K}'$, $\vec{d}_1$, $\vec{d}_2$, and $\vec{d}_3$). Then, in order to project the expanded Hamiltonian $\tilde{H}_o^{(\exp)}(\vec{\kappa})$ onto the subset of states corresponding to the bands nearest to the Fermi energy, we have first changed the basis from $\mathcal{V}_1$ to $\mathcal{V}_4$ (the basis set which diagonalizes the tight-binding Hamiltonian exactly in $\vec{K}'$, i.e. for $\vec{\kappa}=0$), obtaining the matrix $H_0'^{(\exp)}(\vec{\kappa})=(\tilde{U})^\dagger\tilde{H}_0^{(\exp)}(\vec{\kappa})\,\tilde{U}$. The projection of $H_0'^{(\exp)}(\vec{\kappa})$ onto the basis states $|\Phi_1\rangle$ and $|\Phi_4\rangle$ (which correspond near $\vec{K}'$ to the bands nearest to the Fermi energy) is then the 2×2 submatrix obtained from the intersection of its first two rows and of its first two columns (because $|\Phi_1\rangle$ and $|\Phi_4\rangle$ are the first two elements of $\mathcal{V}_4$). At the end of this analytical procedure, we obtain the following 2×2 matrix:

$$
H_{\vec{K}'}(\vec{\kappa}) = \begin{bmatrix} \epsilon_1 & \gamma(\kappa_x+i\kappa_y) \\ \gamma(\kappa_x-i\kappa_y) & \epsilon_1 \end{bmatrix} , \tag{S117}
$$

where γ (which coincides with $\hbar v_F$, with $\hbar$ the reduced Planck constant and v_F the Fermi velocity) is equal to

$$
\gamma = -\frac{\sqrt{3}a_0}{2}\left[u_{11}^2(V_{pp\pi}\sin^2\theta+V_{pp\sigma}\cos^2\theta)-u_{21}^2V_{ss\sigma}+2u_{11}u_{21}\cos\theta V_{sp\sigma}-\frac{1}{2}|u_{31}|^2\sin^2\theta(V_{pp\sigma}-V_{pp\pi})\right] . \tag{S118}
$$

Its eigenvalues are equal to $\epsilon(\vec{\kappa})=\epsilon_1\pm\gamma\sqrt{\kappa_x^2+\kappa_y^2}=\epsilon_1\pm\gamma|\vec{\kappa}|$, which fit quite well the tight-binding bands nearest to the Fermi energy around the $\vec{K}'$ point.

Now let us include also the effect of the spin-orbit coupling (neglecting the Rashba term). Rearranging Eq. (8), the spin-orbit Hamiltonian on the basis of 8 atomic orbitals (differing also for the spin) centered on the same atom $\{|s\uparrow\rangle, |s\downarrow\rangle, |p_x\uparrow\rangle, |p_x\downarrow\rangle, |p_y\uparrow\rangle, |p_y\downarrow\rangle, |p_z\uparrow\rangle, |p_z\downarrow\rangle$ is:

$$
\hat{H}_{SO} = \frac{\Delta_{SO}}{3}\begin{bmatrix}
0 & 0 & 0 & 0 & 0 & 0 & 0 & 0 \\
0 & 0 & 0 & 0 & 0 & 0 & 0 & 0 \\
0 & 0 & 0 & 0 & -i & 0 & 0 & 1 \\
0 & 0 & 0 & 0 & 0 & i & -1 & 0 \\
0 & 0 & i & 0 & 0 & 0 & 0 & -i \\
0 & 0 & 0 & -i & 0 & 0 & -i & 0 \\
0 & 0 & 0 & -1 & 0 & i & 0 & 0 \\
0 & 0 & 1 & 0 & i & 0 & 0 & 0
\end{bmatrix} . \tag{S119}
$$

Since no spin-orbit interaction exists between orbitals on different atoms, on the basis set $\mathcal{V}_1\otimes\{|\uparrow\rangle,|\downarrow\rangle\}=\{|s^A\uparrow\rangle, |s^A\downarrow\rangle, |p_x^A\uparrow\rangle, |p_x^A\downarrow\rangle, |p_y^A\uparrow\rangle, |p_y^A\downarrow\rangle, |p_z^A\uparrow\rangle, |p_z^A\downarrow\rangle, |s^B\uparrow\rangle, |s^B\downarrow\rangle, |p_x^B\uparrow\rangle, |p_x^B\downarrow\rangle, |p_y^B\uparrow\rangle, |p_y^B\downarrow\rangle, |p_z^B\uparrow\rangle, |p_z^B\downarrow\rangle\}$ (the operator $\otimes$ being the tensor product) the spin-orbit contribution to the Hamiltonian is

$$
\tilde{H}_{SO} = \begin{bmatrix} \hat{H}_{SO} & 0 \\ 0 & \hat{H}_{SO} \end{bmatrix} . \tag{S120}
$$

On the other hand, the part of Hamiltonian which does not include the spin-orbit interaction, i.e. $\tilde{H}_0(\vec{k})$, acts identically on spin-up and spin-down orbitals, and thus it can be expressed on the basis set $\mathcal{V}_1\otimes\{|\uparrow\rangle,|\downarrow\rangle\}$ as $\tilde{H}_0(\vec{k})\otimes I_2$ (where I_2 is the 2×2 identity matrix). Therefore, the total Hamiltonian on the basis set $\mathcal{V}_1\otimes\{|\uparrow\rangle,|\downarrow\rangle\}$ is

$\tilde{H}_0(\vec{k}) \otimes I_2 + \tilde{H}_{SO}$. We can repeat our previous treatment on this extended Hamiltonian. We first perform a first-order Taylor expansion around $\vec{K}'$ (only of the first term, because the second term does not depend on $\vec{k}$). Then we move from the basis set $\mathcal{V}_1 \otimes \{|\uparrow\rangle, |\downarrow\rangle\}$ to the basis set $\mathcal{V}_4 \otimes \{|\uparrow\rangle, |\downarrow\rangle\}$ through the matrix $\tilde{U} \otimes I_2$, obtaining:

$$\begin{aligned}
&(\tilde{U} \otimes I_2)^\dagger (\tilde{H}_0^{(\exp)}(\vec{\kappa}) \otimes I_2 + \tilde{H}_{SO})(\tilde{U} \otimes I_2) = \\
&= (\tilde{U} \otimes I_2)^\dagger (\tilde{H}_0^{(\exp)}(\vec{\kappa}) \otimes I_2)(\tilde{U} \otimes I_2) + (\tilde{U} \otimes I_2)^\dagger \tilde{H}_{SO} (\tilde{U} \otimes I_2) = \\
&= H_0'^{(\exp)}(\vec{\kappa}) \otimes I_2 + (\tilde{U} \otimes I_2)^\dagger \tilde{H}_{SO} (\tilde{U} \otimes I_2)\,.
\end{aligned} \tag{S121}$$

Finally, we project the resulting Hamiltonian onto the basis $\{\,|\Phi_1\rangle, |\Phi_4\rangle\,\} \otimes \{\,|\uparrow\rangle, |\downarrow\rangle\,\} = \{\,|\Phi_1 \uparrow\rangle, |\Phi_1 \downarrow\rangle, |\Phi_4 \uparrow\rangle, |\Phi_4 \downarrow\rangle\}$, obtaining:

$$\begin{bmatrix} \epsilon_1 & 0 & \gamma(\kappa_x + i\kappa_y) & 0 \\ 0 & \epsilon_1 & 0 & \gamma(\kappa_x + i\kappa_y) \\ \gamma(\kappa_x - i\kappa_y) & 0 & \epsilon_1 & 0 \\ 0 & \gamma(\kappa_x - i\kappa_y) & 0 & \epsilon_1 \end{bmatrix} + \lambda_{SO}^{1st} \begin{bmatrix} -1 & 0 & 0 & 0 \\ 0 & 1 & 0 & 0 \\ 0 & 0 & 1 & 0 \\ 0 & 0 & 0 & -1 \end{bmatrix}, \tag{S122}$$

with

$$\lambda_{SO}^{1st} = \frac{\Delta_{SO}}{3} |u_{31}|^2\,. \tag{S123}$$

The first term of Eq. (S122) corresponds to $H_{\vec{K}'}(\vec{\kappa}) \otimes I_2$, while its second term is obtained performing the product $(\tilde{U} \otimes I_2)^\dagger \tilde{H}_{SO} (\tilde{U} \otimes I_2)$ and considering its 4×4 submatrix given by the intersection of its first 4 rows and of its first 4 columns (since $\{\,|\Phi_1 \uparrow\rangle, |\Phi_1 \downarrow\rangle, |\Phi_4 \uparrow\rangle, |\Phi_4 \downarrow\rangle\}$ represent the first 4 elements of the basis set $\mathcal{V}_4 \otimes \{|\uparrow\rangle, |\downarrow\rangle\}$).

Following this procedure, we have neglected the coupling which exists between the 4 states on which we have performed our projection and the other 12 states that we have discarded (as we have seen, only the Hamiltonian without spin-orbit in $\vec{k} = \vec{K}'$ is diagonal on the basis $\mathcal{V}_4 \otimes \{|\uparrow\rangle, |\downarrow\rangle\}$). The effect of this interaction can be partially recovered adopting the method described in the Appendix B of Ref. [1]. We can rewrite the matrix $H_0'^{(\exp)}(\vec{\kappa}) \otimes I_2 + (\tilde{U} \otimes I_2)^\dagger \tilde{H}_{SO} (\tilde{U} \otimes I_2)$ of Eq. (S121) (where the first term is the Hamiltonian without spin-orbit coupling and the second term is the spin-orbit contribution) in the form

$$\begin{bmatrix} H_\pi & H_n \\ H_n^\dagger & H_\sigma \end{bmatrix}, \tag{S124}$$

where the 4×4 matrix H_π is given by the intersection of the first 4 rows and of the first 4 columns (i.e., the rows and the columns corresponding to the states considered in our projection) of the Hamiltonian of Eq. (S121), the 4×12 matrix H_n is given by the intersection of its first 4 rows and of its 5th-to-16th columns, the 12×4 matrix $H_n^\dagger$ is given by the intersection of its 5th-to-16th rows and of its first 4 columns, and the 12×12 matrix H_σ is given by the intersection of its 5th-to-16th rows and of its 5th-to-16th columns. Since the eigenvalues of H_π are near ϵ_1, the eigenvalues of H_σ are far from it, and H_n is much smaller than $H_\sigma - \epsilon_1 I_{12}$, following the procedure described in the Appendix B of Ref. [1] we can approximate the Hamiltonian of Eq. (S121) near ϵ_1 with $H_\pi - H_n (H_\sigma - \epsilon_1 I_{12})^{-1} H_n^\dagger$ (where I_{12} is the 12×12 identity matrix). The matrix H_π represents the projection of Eq. (S121) on the first 4 states of the basis $\mathcal{V}_4 \otimes \{|\uparrow\rangle, |\downarrow\rangle\}$, i.e. exactly Eq. (S122). Instead, the term $-H_n (H_\sigma - \epsilon_1 I_{12})^{-1} H_n^\dagger$ gives rise to an effective second-order spin-orbit contribution. Let us approximate [1] the Hamiltonian of Eq. (S121) with its value in $\vec{\kappa} = 0$ (i.e. in $\vec{k} = \vec{K}'$); in this case its part without spin-orbit is the diagonal matrix $H_0'(\vec{K}') \otimes I_2$. Therefore, the matrix H_n derives only from the spin-orbit part of Eq. (S121) and is given by the intersection of the first 4 rows and of the 5th-to-16th columns of $(\tilde{U} \otimes I_2)^\dagger \tilde{H}_{SO} (\tilde{U} \otimes I_2)$. Instead, in H_σ the contribution of the Hamiltonian without spin-orbit dominates on that of spin-orbit and we can approximate H_σ with the intersection of the 5th-to-16th rows and of the 5th-to-16th columns of $H_0'(\vec{K}') \otimes I_2$. Performing the calculation, we obtain the following quantity:

$$H_{SO}^{2nd} = \begin{bmatrix} b_1 & 0 & 0 & 0 \\ 0 & b_2 & 0 & 0 \\ 0 & 0 & b_2 & 0 \\ 0 & 0 & 0 & b_1 \end{bmatrix}, \tag{S125}$$

with

$$b_1 = \left(\frac{\Delta_{SO}}{3}\right)^2 \left(\frac{2\,u_{12}^2|u_{31}|^2 + 4\,u_{11}u_{12}u_{31}u_{32} + 2\,u_{11}^2|u_{32}|^2 + |u_{31}|^2|u_{32}|^2}{\epsilon_1 - \epsilon_2} + \right.$$
$$\left. + \frac{2\,u_{13}^2|u_{31}|^2 + 4\,u_{11}u_{13}u_{31}u_{33} + 2\,u_{11}^2|u_{33}|^2 + |u_{31}|^2|u_{33}|^2}{\epsilon_1 - \epsilon_3}\right), \tag{S126}$$
$$b_2 = \left(\frac{\Delta_{SO}}{3}\right)^2 \left(\frac{|u_{31}|^2|u_{32}|^2}{\epsilon_1 - \epsilon_2} + \frac{|u_{31}|^2|u_{33}|^2}{\epsilon_1 - \epsilon_3} + \frac{2\,\epsilon_1\,u_{11}^2}{\epsilon_1^2 - V_1^2}\right).$$

Adding Eq. (S125) to Eq. (S122), we obtain the Hamiltonian

$$H_{\vec{K}'}(\vec{\kappa}) \otimes I_2 + \begin{bmatrix} d_1 & 0 & 0 & 0 \\ 0 & d_2 & 0 & 0 \\ 0 & 0 & d_2 & 0 \\ 0 & 0 & 0 & d_1 \end{bmatrix}, \tag{S127}$$

where

$$d_1 = b_1 - \lambda_{SO}^{1st}\,, \quad d_2 = b_2 + \lambda_{SO}^{1st}\,. \tag{S128}$$

II. $\vec{K} \cdot \vec{P}$ ANALYSIS AT THE POINT $\vec{K}$

The $\vec{k} \cdot \vec{p}$ analysis around $\vec{K}$ can be performed in a very similar way, following again the procedure of Ref. [1].

We first diagonalize the tight-binding Hamiltonian exactly at the point $\vec{K} = [-4\pi/(3a_0),\, 0,\, 0]^T$. In $\vec{K}$ we have that $e^{i\vec{K}\cdot\vec{d}_1} = e^{-i2\pi/3}$, $e^{i\vec{K}\cdot\vec{d}_2} = e^{i2\pi/3}$, $e^{i\vec{K}\cdot\vec{d}_3} = 1$, and therefore the Hamiltonian (S43) becomes:

$$\tilde{H}_0(\vec{K}) = \begin{bmatrix} \Delta & 0 & 0 & 0 & 0 & -iV_2' & -V_2' & 0 \\ 0 & 0 & 0 & 0 & iV_2' & V_1' & iV_1' & iV_3' \\ 0 & 0 & 0 & 0 & V_2' & iV_1' & -V_1' & V_3' \\ 0 & 0 & 0 & \Delta_{pz} & 0 & iV_3' & V_3' & 0 \\ 0 & -iV_2' & V_2' & 0 & \Delta & 0 & 0 & 0 \\ iV_2' & V_1' & -iV_1' & -iV_3' & 0 & 0 & 0 & 0 \\ -V_2' & -iV_1' & -V_1' & V_3' & 0 & 0 & 0 & 0 \\ 0 & -iV_3' & V_3' & 0 & 0 & 0 & 0 & \Delta_{pz} \end{bmatrix} \tag{S129}$$

with

$$V_1' = \frac{3}{4}\sin^2\theta\,(V_{pp\pi} - V_{pp\sigma})\,, \quad V_2' = \frac{3}{2}\sin\theta\, V_{sp\sigma}\,, \quad V_3' = \frac{3}{2}\sin\theta\,\cos\theta\,(V_{pp\pi} - V_{pp\sigma})\,. \tag{S130}$$

Let us move from the basis set $\mathcal{V}_1$ to the basis set $\mathcal{N}_2 = \{|p_z^A\rangle, |s^A\rangle, |\psi_2^B\rangle, |p_z^B\rangle, |s^B\rangle, |\psi_1^A\rangle, |\psi_3\rangle, |\psi_4\rangle\}$, where

$$|\psi_1^A\rangle = -\frac{1}{\sqrt{2}}(|p_x^A\rangle - i\,|p_y^A\rangle)\,, \quad |\psi_2^B\rangle = \frac{1}{\sqrt{2}}(|p_x^B\rangle + i\,|p_y^B\rangle)\,,$$
$$|\psi_3\rangle = -\frac{1}{2}(|p_x^A\rangle + i\,|p_y^A\rangle) - \frac{1}{2}(|p_x^B\rangle - i\,|p_y^B\rangle)\,, \quad |\psi_4\rangle = \frac{1}{2}(|p_x^A\rangle + i\,|p_y^A\rangle) - \frac{1}{2}|(p_x^B\rangle - i\,|p_y^B\rangle) \tag{S131}$$

through the unitary transformation

$$\tilde{W}_1 = \begin{bmatrix} 0 & 1 & 0 & 0 & 0 & 0 & 0 & 0 \\ 0 & 0 & 0 & 0 & 0 & -\frac{1}{\sqrt{2}} & -\frac{1}{2} & \frac{1}{2} \\ 0 & 0 & 0 & 0 & 0 & \frac{i}{\sqrt{2}} & -\frac{i}{2} & \frac{i}{2} \\ 1 & 0 & 0 & 0 & 0 & 0 & 0 & 0 \\ 0 & 0 & 0 & 0 & 1 & 0 & 0 & 0 \\ 0 & 0 & \frac{1}{\sqrt{2}} & 0 & 0 & 0 & -\frac{1}{2} & -\frac{1}{2} \\ 0 & 0 & \frac{i}{\sqrt{2}} & 0 & 0 & 0 & \frac{i}{2} & \frac{i}{2} \\ 0 & 0 & 0 & 1 & 0 & 0 & 0 & 0 \end{bmatrix} . \tag{S132}$$

The Hamiltonian becomes

$$H_1(\vec{K}) = (\tilde{W}_1)^\dagger \, \tilde{H}_0(\vec{K}) \, \tilde{W}_1 = \begin{bmatrix} \Delta_{pz} & 0 & iV_3 & 0 & 0 & 0 & 0 & 0 \\ 0 & \Delta & -iV_2 & 0 & 0 & 0 & 0 & 0 \\ -iV_3 & iV_2 & 0 & 0 & 0 & 0 & 0 & 0 \\ 0 & 0 & 0 & \Delta_{pz} & 0 & iV_3 & 0 & 0 \\ 0 & 0 & 0 & 0 & \Delta & iV_2 & 0 & 0 \\ 0 & 0 & 0 & -iV_3 & -iV_2 & 0 & 0 & 0 \\ 0 & 0 & 0 & 0 & 0 & 0 & V_1 & 0 \\ 0 & 0 & 0 & 0 & 0 & 0 & 0 & -V_1 \end{bmatrix} , \tag{S133}$$

with $V_1 = 2V_1'$, $V_2 = \sqrt{2}V_2'$, and $V_3 = \sqrt{2}V_3'$. This Hamiltonian is a block diagonal matrix with the following blocks on the diagonal:

$$H_A^{\vec{K}} = \begin{bmatrix} \Delta_{pz} & 0 & iV_3 \\ 0 & \Delta & -iV_2 \\ -iV_3 & iV_2 & 0 \end{bmatrix} , \quad H_B^{\vec{K}} = \begin{bmatrix} \Delta_{pz} & 0 & iV_3 \\ 0 & \Delta & iV_2 \\ -iV_3 & -iV_2 & 0 \end{bmatrix} , \quad V_1 , \quad -V_1 . \tag{S134}$$

The eigenvalues ϵ_i (with $i = 1, 2, 3$, where $\epsilon_3 < \epsilon_1 < \epsilon_2$) of $H_A^{\vec{K}}$ are the roots of the equation $\det(H_A^{\vec{K}} - \epsilon I_3) = 0$ (where I_3 is the 3×3 identity matrix), i.e. of

$$\epsilon^3 - (\Delta + \Delta_{pz})\epsilon^2 - (-\Delta_{pz}\Delta + V_2^2 + V_3^2)\epsilon + \Delta_{pz}V_2^2 + \Delta V_3^2 = 0 \tag{S135}$$

(the same as at the $\vec{K}'$ point), while the corresponding orthonormalized eigenvectors are

$$\begin{bmatrix} \frac{1}{\alpha_i} \\ \frac{V_2(\epsilon_i - \Delta_{pz})}{\alpha_i(\Delta - \epsilon_i)V_3} \\ -\frac{i(\epsilon_i - \Delta_{pz})}{\alpha_i V_3} \end{bmatrix} \equiv \begin{bmatrix} w_{1i} \\ w_{2i} \\ w_{3i} \end{bmatrix} \tag{S136}$$

where

$$\alpha_i = \sqrt{1 + \left(\frac{V_2(\epsilon_i - \Delta_{pz})}{(\Delta - \epsilon_i)V_3}\right)^2 + \left(\frac{\epsilon_i - \Delta_{pz}}{V_3}\right)^2} . \tag{S137}$$

The matrix of the eigenvectors W_A diagonalizes $H_A^{\vec{K}}$, i.e. $W_A^\dagger H_A^{\vec{K}} W_A = \Lambda_A$, with

$$W_A = \begin{bmatrix} w_{11} & w_{12} & w_{13} \\ w_{21} & w_{22} & w_{23} \\ w_{31} & w_{32} & w_{33} \end{bmatrix}, \quad \Lambda_A = \begin{bmatrix} \epsilon_1 & 0 & 0 \\ 0 & \epsilon_2 & 0 \\ 0 & 0 & \epsilon_3 \end{bmatrix}. \tag{S138}$$

The eigenvalues of $H_B^{\vec{K}}$ are again the roots of Eq. (S135) and thus coincide with those of $H_A^{\vec{K}}$, but its eigenvectors have the form $[\, w_{1i},\, -w_{2i},\, w_{3i}\,]^T$. Therefore, we have that $W_B^\dagger H_B^{\vec{K}} W_B = \Lambda_B$, with

$$W_B = \begin{bmatrix} w_{11} & w_{12} & w_{13} \\ -w_{21} & -w_{22} & -w_{23} \\ w_{31} & w_{32} & w_{33} \end{bmatrix}, \quad \Lambda_B = \Lambda_A\,. \tag{S139}$$

Therefore, $(W_2')^\dagger H_1(\vec{K}) W_2' = H_2(\vec{K})$, where

$$W_2' = \begin{bmatrix} w_{11} & w_{12} & w_{13} & 0 & 0 & 0 & 0 & 0 \\ w_{21} & w_{22} & w_{23} & 0 & 0 & 0 & 0 & 0 \\ w_{31} & w_{32} & w_{33} & 0 & 0 & 0 & 0 & 0 \\ 0 & 0 & 0 & w_{11} & w_{12} & w_{13} & 0 & 0 \\ 0 & 0 & 0 & -w_{21} & -w_{22} & -w_{23} & 0 & 0 \\ 0 & 0 & 0 & w_{31} & w_{32} & w_{33} & 0 & 0 \\ 0 & 0 & 0 & 0 & 0 & 0 & 1 & 0 \\ 0 & 0 & 0 & 0 & 0 & 0 & 0 & 1 \end{bmatrix}, \quad H_2(\vec{K}) = \begin{bmatrix} \epsilon_1 & 0 & 0 & 0 & 0 & 0 & 0 & 0 \\ 0 & \epsilon_2 & 0 & 0 & 0 & 0 & 0 & 0 \\ 0 & 0 & \epsilon_3 & 0 & 0 & 0 & 0 & 0 \\ 0 & 0 & 0 & \epsilon_1 & 0 & 0 & 0 & 0 \\ 0 & 0 & 0 & 0 & \epsilon_2 & 0 & 0 & 0 \\ 0 & 0 & 0 & 0 & 0 & \epsilon_3 & 0 & 0 \\ 0 & 0 & 0 & 0 & 0 & 0 & V_1 & 0 \\ 0 & 0 & 0 & 0 & 0 & 0 & 0 & -V_1 \end{bmatrix}. \tag{S140}$$

In this way, we have moved from the basis set $\mathcal{N}_2$ to the basis set $\mathcal{N}_3 = \{|\Psi_1\rangle, |\Psi_2\rangle, |\Psi_3\rangle, |\Psi_4\rangle, |\Psi_5\rangle, |\Psi_6\rangle, |\Psi_7\rangle, |\Psi_8\rangle)$, where $|\Psi_1\rangle = w_{11}|p_z^A\rangle + w_{21}|s^A\rangle + w_{31}|\psi_2^B\rangle$, $|\Psi_2\rangle = w_{12}|p_z^A\rangle + w_{22}|s^A\rangle + w_{32}|\psi_2^B\rangle$, $|\Psi_3\rangle = w_{13}|p_z^A\rangle + w_{23}|s^A\rangle + w_{33}|\psi_2^B\rangle$, $|\Psi_4\rangle = w_{11}|p_z^B\rangle - w_{21}|s^B\rangle + w_{31}|\psi_1^A\rangle$, $|\Psi_5\rangle = w_{12}|p_z^B\rangle - w_{22}|s^B\rangle + w_{32}|\psi_1^A\rangle$, $|\Psi_6\rangle = w_{13}|p_z^B\rangle - w_{23}|s^B\rangle + w_{33}|\psi_1^A\rangle$, $|\Psi_7\rangle = |\psi_3\rangle$, and $|\Psi_8\rangle = |\psi_4\rangle$.

Substituting the values of the NNTB parameters of Table 1 into the expressions of V_1, V_2 and V_3 and solving Eq. (S135), we obtain that $\epsilon_3 = -9.8374$ eV (double degenerate), $V_1 = -4.1959$ eV, $\epsilon_1 = -1.7430$ eV (double degenerate), $\epsilon_2 = 2.4555$ eV (double degenerate), $-V_1 = 4.1959$ eV. Therefore, the Fermi energy ϵ_F (which leaves half of the energy levels occupied, i.e. with values lower than ϵ_F, and half unoccupied, i.e. with values higher than ϵ_F) is located at ϵ_1 and thus in our analysis around $\vec{K}$ we have focused on the states corresponding to this eigenvalue. Following Ref. [1], we have permuted the position of the basis states in such a way as to group the states with the same eigenvectors through a final change of basis $(W_2'')^\dagger H_2(\vec{K}) W_2'' = H_0'(\vec{K})$, where

$$W_2'' = \begin{bmatrix} 1 & 0 & 0 & 0 & 0 & 0 & 0 & 0 & 0 \\ 0 & 0 & 1 & 0 & 0 & 0 & 0 & 0 & 0 \\ 0 & 0 & 0 & 0 & 1 & 0 & 0 & 0 & 0 \\ 0 & 1 & 0 & 0 & 0 & 0 & 0 & 0 & 0 \\ 0 & 0 & 0 & 1 & 0 & 0 & 0 & 0 & 0 \\ 0 & 0 & 0 & 0 & 0 & 1 & 0 & 0 & 0 \\ 0 & 0 & 0 & 0 & 0 & 0 & 0 & 1 & 0 \\ 0 & 0 & 0 & 0 & 0 & 0 & 0 & 0 & 1 \end{bmatrix}, \quad H_0'(\vec{K}) = \begin{bmatrix} \epsilon_1 & 0 & 0 & 0 & 0 & 0 & 0 & 0 \\ 0 & \epsilon_1 & 0 & 0 & 0 & 0 & 0 & 0 \\ 0 & 0 & \epsilon_2 & 0 & 0 & 0 & 0 & 0 \\ 0 & 0 & 0 & \epsilon_2 & 0 & 0 & 0 & 0 \\ 0 & 0 & 0 & 0 & \epsilon_3 & 0 & 0 & 0 \\ 0 & 0 & 0 & 0 & 0 & \epsilon_3 & 0 & 0 \\ 0 & 0 & 0 & 0 & 0 & 0 & V_1 & 0 \\ 0 & 0 & 0 & 0 & 0 & 0 & 0 & -V_1 \end{bmatrix}, \tag{S141}$$

in this way moving from the basis set $\mathcal{N}_3$ to the basis set $\mathcal{N}_4 = \{|\Psi_1\rangle, |\Psi_4\rangle, |\Psi_2\rangle, |\Psi_5\rangle, |\Psi_3\rangle, |\Psi_6\rangle, |\Psi_7\rangle, |\Psi_8\rangle)$.

The overall unitary matrix $\tilde{W}$ corresponding to the basis change from $\mathcal{V}_1$ to $\mathcal{N}_4$, i.e. such that $(\tilde{W})^\dagger \tilde{H}_0(\vec{K}) \tilde{W} = H_0'(\vec{K})$,

is

$$\tilde{W} = \tilde{W}_1 W_2' W_2'' = \begin{bmatrix} w_{21} & 0 & w_{22} & 0 & w_{23} & 0 & 0 & 0 \\ 0 & -\frac{w_{31}}{\sqrt{2}} & 0 & -\frac{w_{32}}{\sqrt{2}} & 0 & -\frac{w_{33}}{\sqrt{2}} & -\frac{1}{2} & \frac{1}{2} \\ 0 & \frac{iw_{31}}{\sqrt{2}} & 0 & \frac{iw_{32}}{\sqrt{2}} & 0 & \frac{iw_{33}}{\sqrt{2}} & -\frac{i}{2} & \frac{i}{2} \\ w_{11} & 0 & w_{12} & 0 & w_{13} & 0 & 0 & 0 \\ 0 & -w_{21} & 0 & -w_{22} & 0 & -w_{23} & 0 & 0 \\ \frac{w_{31}}{\sqrt{2}} & 0 & \frac{w_{32}}{\sqrt{2}} & 0 & \frac{w_{33}}{\sqrt{2}} & 0 & -\frac{1}{2} & -\frac{1}{2} \\ \frac{iw_{31}}{\sqrt{2}} & 0 & \frac{iw_{32}}{\sqrt{2}} & 0 & \frac{iw_{33}}{\sqrt{2}} & 0 & \frac{i}{2} & \frac{i}{2} \\ 0 & w_{11} & 0 & w_{12} & 0 & w_{13} & 0 & 0 \end{bmatrix} . \tag{S142}$$

In analogy with Ref. [1], we have obtained the $\vec{k}\cdot\vec{p}$ Hamiltonian of stanene around $\vec{K}$ performing a low-order expansion around $\vec{K}$ of the tight-binding Hamiltonian of Eq. (S43) and reducing the matrix size through projection onto the subset of states we are mainly interested in (i.e., those with energy nearest to the Fermi energy).
More in detail, in $\tilde{H}_o(\vec{k})$ (see Eq. (S43)) we have substituted each occurrence of $\vec{k}$ with $\vec{K}+\vec{\kappa}$ (where $\vec{\kappa}=\vec{k}-\vec{K}$ represents the distance in the reciprocal space between $\vec{K}$ and $\vec{k}$); then we have performed a Taylor expansion around $\vec{\kappa}=0$ (which corresponds to $\vec{k}=\vec{K}$) of all the elements of the matrix. Since we are interested only in the behavior of the dispersion relations near $\vec{K}$ (i.e. for small values of $\vec{\kappa}$) and from the DFT and tight-binding results it appears that the dispersion relations around $\vec{K}$ are nearly linear, we have kept only the first-order terms of the expansion. The dependence on $\vec{k}$ appears in the elements of $\tilde{H}_o(\vec{k})$ (see Eq. (S44)) only through the complex exponentials $e^{i\vec{k}\cdot\vec{d}_i}$; therefore, the first-order Taylor expansion $\tilde{H}_o^{(\mathrm{exp})}(\vec{\kappa})$ of the Hamiltonian $\tilde{H}_o(\vec{k})$ can be simply performed substituting the following quantities to the exponentials $e^{i\vec{k}\cdot\vec{d}_i}$ in all the elements of the matrix (Eq. (S44)):

$$\begin{aligned} e^{i\vec{k}\cdot\vec{d}_1} &= e^{i\vec{K}\cdot\vec{d}_1}e^{i\vec{\kappa}\cdot\vec{d}_1} \approx e^{i\vec{K}\cdot\vec{d}_1}(1+i\vec{\kappa}\cdot\vec{d}_1) = \left(-\frac{1}{2}-i\frac{\sqrt{3}}{2}\right)\left(1+i\frac{a_0}{2}\kappa_x+i\frac{a_0}{2\sqrt{3}}\kappa_y\right) , \\ e^{i\vec{k}\cdot\vec{d}_2} &= e^{i\vec{K}\cdot\vec{d}_2}e^{i\vec{\kappa}\cdot\vec{d}_2} \approx e^{i\vec{K}\cdot\vec{d}_2}(1+i\vec{\kappa}\cdot\vec{d}_2) = \left(-\frac{1}{2}+i\frac{\sqrt{3}}{2}\right)\left(1-i\frac{a_0}{2}\kappa_x+i\frac{a_0}{2\sqrt{3}}\kappa_y\right) , \\ e^{i\vec{k}\cdot\vec{d}_3} &= e^{i\vec{K}\cdot\vec{d}_3}e^{i\vec{\kappa}\cdot\vec{d}_3} \approx e^{i\vec{K}\cdot\vec{d}_3}(1+i\vec{\kappa}\cdot\vec{d}_3) = 1-i\frac{a_0}{\sqrt{3}}\kappa_y \end{aligned} \tag{S143}$$

(where we have substituted the coordinates of $\vec{K}$, $\vec{d}_1$, $\vec{d}_2$, and $\vec{d}_3$). Then, in order to project the expanded Hamiltonian $\tilde{H}_o^{(\mathrm{exp})}(\vec{\kappa})$ onto the subset of states corresponding to the bands nearest to the Fermi energy, we have first changed the basis from $\mathcal{V}_1$ to $\mathcal{N}_4$ (the basis set which diagonalizes the tight-binding Hamiltonian exactly in $\vec{K}$, i.e. for $\vec{\kappa}=0$), obtaining the matrix $H_0'^{(\mathrm{exp})}(\vec{\kappa}) = (\tilde{W})^\dagger \tilde{H}_0^{(\mathrm{exp})}(\vec{\kappa})\,\tilde{W}$. The projection of $H_0'^{(\mathrm{exp})}(\vec{\kappa})$ onto the basis states $|\Psi_1\rangle$ and $|\Psi_4\rangle$ (which correspond near $\vec{K}$ to the bands nearest to the Fermi energy) is then the 2×2 submatrix obtained from the intersection of its first two rows and of its first two columns (because $|\Psi_1\rangle$ and $|\Psi_4\rangle$ are the first two elements of $\mathcal{N}_4$). At the end of this analytical procedure, we obtain the following 2×2 matrix:

$$H_{\vec{K}}(\vec{\kappa}) = \begin{bmatrix} \epsilon_1 & -\gamma(\kappa_x - i\kappa_y) \\ -\gamma(\kappa_x + i\kappa_y) & \epsilon_1 \end{bmatrix} , \tag{S144}$$

where γ (which coincides with $\hbar v_F$, with $\hbar$ the reduced Planck constant and v_F the Fermi velocity) is equal to

$$\gamma = -\frac{\sqrt{3}a_0}{2}\left[w_{11}^2(V_{pp\pi}\sin^2\theta + V_{pp\sigma}\cos^2\theta) - w_{21}^2 V_{ss\sigma} + 2w_{11}w_{21}\cos\theta V_{sp\sigma} - \frac{1}{2}|w_{31}|^2\sin^2\theta(V_{pp\sigma}-V_{pp\pi})\right] \tag{S145}$$

(which is identical to Eq. (S118)). Its eigenvalues are equal to $\epsilon(\vec{\kappa}) = \epsilon_1 \pm \gamma\sqrt{\kappa_x^2+\kappa_y^2} = \epsilon_1 \pm \gamma|\vec{\kappa}|$, which fit the tight-binding bands quite well around the $\vec{K}$ point.

Now let us include also the effect of the spin-orbit coupling (neglecting the Rashba term). Rearranging Eq. (8), the spin-orbit Hamiltonian on the basis of 8 atomic orbitals (differing also for the spin) centered on the same atom $\{|s\uparrow\rangle, |s\downarrow\rangle, |p_x\uparrow\rangle, |p_x\downarrow\rangle, |p_y\uparrow\rangle, |p_y\downarrow\rangle, |p_z\uparrow\rangle, |p_z\downarrow\rangle$ is:

$$\hat{H}_{SO} = \frac{\Delta_{SO}}{3}\begin{bmatrix} 0 & 0 & 0 & 0 & 0 & 0 & 0 & 0 \\ 0 & 0 & 0 & 0 & 0 & 0 & 0 & 0 \\ 0 & 0 & 0 & 0 & -i & 0 & 0 & 1 \\ 0 & 0 & 0 & 0 & 0 & i & -1 & 0 \\ 0 & 0 & i & 0 & 0 & 0 & 0 & -i \\ 0 & 0 & 0 & -i & 0 & 0 & -i & 0 \\ 0 & 0 & 0 & -1 & 0 & i & 0 & 0 \\ 0 & 0 & 1 & 0 & i & 0 & 0 & 0 \end{bmatrix}. \tag{S146}$$

Since no spin-orbit interaction exists between orbitals on different atoms, on the basis set $\mathcal{V}_1 \otimes \{|\uparrow\rangle, |\downarrow\rangle\} = \{|s^A\uparrow\rangle, |s^A\downarrow\rangle, |p_x^A\uparrow\rangle, |p_x^A\downarrow\rangle, |p_y^A\uparrow\rangle, |p_y^A\downarrow\rangle, |p_z^A\uparrow\rangle, |p_z^A\downarrow\rangle, |s^B\uparrow\rangle, |s^B\downarrow\rangle, |p_x^B\uparrow\rangle, |p_x^B\downarrow\rangle, |p_y^B\uparrow\rangle, |p_y^B\downarrow\rangle, |p_z^B\uparrow\rangle, |p_z^B\downarrow\rangle\}$ (the operator $\otimes$ being the tensor product) the spin-orbit contribution to the Hamiltonian is

$$\tilde{H}_{SO} = \begin{bmatrix} \hat{H}_{SO} & 0 \\ 0 & \hat{H}_{SO} \end{bmatrix}. \tag{S147}$$

On the other hand, the part of Hamiltonian which does not include the spin-orbit interaction, i.e. $\tilde{H}_0(\vec{k})$, acts identically on spin-up and spin-down orbitals, and thus it can be expressed on the basis set $\mathcal{V}_1 \otimes \{|\uparrow\rangle, |\downarrow\rangle\}$ as $\tilde{H}_0(\vec{k}) \otimes I_2$ (where I_2 is the 2×2 identity matrix). Therefore, the total Hamiltonian on the basis set $\mathcal{V}_1 \otimes \{|\uparrow\rangle, |\downarrow\rangle\}$ is $\tilde{H}_0(\vec{k}) \otimes I_2 + \tilde{H}_{SO}$. We can repeat our previous treatment on this extended Hamiltonian. We first perform a first-order Taylor expansion around $\vec{K}$ (only of the first term, because the second term does not depend on $\vec{k}$). Then we move from the basis set $\mathcal{V}_1 \otimes \{|\uparrow\rangle, |\downarrow\rangle\}$ to the basis set $\mathcal{N}_4 \otimes \{|\uparrow\rangle, |\downarrow\rangle\}$ through the matrix $\tilde{W} \otimes I_2$, obtaining:

$$\begin{aligned} &(\tilde{W} \otimes I_2)^\dagger (\tilde{H}_0^{(\exp)}(\vec{\kappa}) \otimes I_2 + \tilde{H}_{SO})(\tilde{W} \otimes I_2) = \\ &= (\tilde{W} \otimes I_2)^\dagger (\tilde{H}_0^{(\exp)}(\vec{\kappa}) \otimes I_2)(\tilde{W} \otimes I_2) + (\tilde{W} \otimes I_2)^\dagger \tilde{H}_{SO}(\tilde{W} \otimes I_2) = \\ &= H_0'^{(\exp)}(\vec{\kappa}) \otimes I_2 + (\tilde{W} \otimes I_2)^\dagger \tilde{H}_{SO}(\tilde{W} \otimes I_2)\,. \end{aligned} \tag{S148}$$

Finally, we project the resulting Hamiltonian onto the basis $\{\,|\Psi_1\rangle,\,|\Psi_4\rangle\,\} \otimes \{\,|\uparrow\rangle,\,|\downarrow\rangle\,\} = \{\,|\Psi_1\uparrow\rangle,\,|\Psi_1\downarrow\rangle,\,|\Psi_4\uparrow\rangle,\,|\Psi_4\downarrow\rangle\}$, obtaining:

$$\begin{bmatrix} \epsilon_1 & 0 & -\gamma(\kappa_x - i\kappa_y) & 0 \\ 0 & \epsilon_1 & 0 & -\gamma(\kappa_x - i\kappa_y) \\ -\gamma(\kappa_x + i\kappa_y) & 0 & \epsilon_1 & 0 \\ 0 & -\gamma(\kappa_x + i\kappa_y) & 0 & \epsilon_1 \end{bmatrix} + \lambda_{SO}^{1st}\begin{bmatrix} 1 & 0 & 0 & 0 \\ 0 & -1 & 0 & 0 \\ 0 & 0 & -1 & 0 \\ 0 & 0 & 0 & 1 \end{bmatrix}, \tag{S149}$$

with

$$\lambda_{SO}^{1st} = \frac{\Delta_{SO}}{3}|w_{31}|^2 \tag{S150}$$

(which is identical to Eq. (S123)). The first term of Eq. (S149) corresponds to $H_{\vec{K}}(\vec{\kappa}) \otimes I_2$, while its second term is obtained performing the product $(\tilde{W} \otimes I_2)^\dagger \tilde{H}_{SO}(\tilde{W} \otimes I_2)$ and considering its 4×4 submatrix given by the intersection of its first 4 rows and of its first 4 columns (since $\{\,|\Psi_1\uparrow\rangle,\,|\Psi_1\downarrow\rangle,\,|\Psi_4\uparrow\rangle,\,|\Psi_4\downarrow\rangle\}$ represent the first 4 elements of the basis set $\mathcal{N}_4 \otimes \{|\uparrow\rangle, |\downarrow\rangle\}$).

Following this procedure, we have neglected the coupling which exists between the 4 states on which we have performed our projection and the other 12 states that we have discarded (as we have seen, only the Hamiltonian without spin-orbit in $\vec{k} = \vec{K}$ is diagonal on the basis $\mathcal{N}_4 \otimes \{|\uparrow\rangle, |\downarrow\rangle\}$). The effect of this interaction can be partially recovered adopting the method described in the Appendix B of Ref. [1]. We can rewrite the matrix $H_0'^{(\exp)}(\vec{\kappa}) \otimes I_2 + (\tilde{W} \otimes I_2)^\dagger \tilde{H}_{SO}(\tilde{W} \otimes I_2)$ of Eq. (S148) (where the first term is the Hamiltonian without spin-orbit coupling and the second term is the spin-orbit contribution) in the form

$$\begin{bmatrix} H_\pi & H_n \\ H_n^\dagger & H_\sigma \end{bmatrix}, \tag{S151}$$

where the 4×4 matrix H_π is given by the intersection of the first 4 rows and of the first 4 columns (i.e., the rows and the columns corresponding to the states considered in our projection) of the Hamiltonian of Eq. (S148), the 4×12 matrix H_n is given by the intersection of its first 4 rows and of its 5th-to-16th columns, the 12×4 matrix $H_n^\dagger$ is given by the intersection of its 5th-to-16th rows and of its first 4 columns, and the 12×12 matrix H_σ is given by the intersection of its 5th-to-16th rows and of its 5th-to-16th columns. Since the eigenvalues of H_π are near ϵ_1, the eigenvalues of H_σ are far from it, and H_n is much smaller than $H_\sigma - \epsilon_1 I_{12}$, following the procedure described in the Appendix B of Ref. [1] we can approximate the Hamiltonian of Eq. (S148) near ϵ_1 with $H_\pi - H_n(H_\sigma - \epsilon_1 I_{12})^{-1} H_n^\dagger$ (where I_{12} is the 12×12 identity matrix). The matrix H_π represents the projection of Eq. (S148) on the first 4 states of the basis $\mathcal{N}_4 \otimes \{|\uparrow\rangle, |\downarrow\rangle\}$, i.e. exactly Eq. (S149). Instead, the term $-H_n(H_\sigma - \epsilon_1 I_{12})^{-1} H_n^\dagger$ gives rise to an effective second-order spin-orbit contribution. Let us approximate [1] the Hamiltonian of Eq. (S148) with its value in $\vec{\kappa} = 0$ (i.e. in $\vec{k} = \vec{K}$); in this case its part without spin-orbit is the diagonal matrix $H_0'(\vec{K}) \otimes I_2$. Therefore, the matrix H_n derives only from the spin-orbit part of Eq. (S148) and is given by the intersection of the first 4 rows and of the 5th-to-16th columns of $(\tilde{W} \otimes I_2)^\dagger \tilde{H}_{SO} (\tilde{W} \otimes I_2)$. Instead, in H_σ the contribution of the Hamiltonian without spin-orbit dominates on that of spin-orbit and we can approximate H_σ with the intersection of the 5th-to-16th rows and of the 5th-to-16th columns of $H_0'(\vec{K}) \otimes I_2$. Performing the calculation, we obtain the following quantity:

$$H_{SO}^{2nd} = \begin{bmatrix} b_2 & 0 & 0 & 0 \\ 0 & b_1 & 0 & 0 \\ 0 & 0 & b_1 & 0 \\ 0 & 0 & 0 & b_2 \end{bmatrix}, \tag{S152}$$

with

$$\begin{aligned} b_1 &= \left(\frac{\Delta_{SO}}{3}\right)^2 \left(\frac{2\, w_{12}^2 |w_{31}|^2 + 4\, w_{11} w_{12} w_{31} w_{32} + 2\, w_{11}^2 |w_{32}|^2 + |w_{31}|^2 |w_{32}|^2}{\epsilon_1 - \epsilon_2} + \right. \\ &\qquad \left. + \frac{2\, w_{13}^2 |w_{31}|^2 + 4\, w_{11} w_{13} w_{31} w_{33} + 2\, w_{11}^2 |w_{33}|^2 + |w_{31}|^2 |w_{33}|^2}{\epsilon_1 - \epsilon_3} \right), \\ b_2 &= \left(\frac{\Delta_{SO}}{3}\right)^2 \left(\frac{|w_{31}|^2 |w_{32}|^2}{\epsilon_1 - \epsilon_2} + \frac{|w_{31}|^2 |w_{33}|^2}{\epsilon_1 - \epsilon_3} + \frac{2\, \epsilon_1\, w_{11}^2}{\epsilon_1^2 - V_1^2} \right) \end{aligned} \tag{S153}$$

(which coincide with the definitions reported in Eq. (S126)). Adding Eq. (S152) to Eq. (S149), we obtain the Hamiltonian

$$H_{\vec{K}}(\vec{\kappa}) \otimes I_2 + \begin{bmatrix} d_2 & 0 & 0 & 0 \\ 0 & d_1 & 0 & 0 \\ 0 & 0 & d_1 & 0 \\ 0 & 0 & 0 & d_2 \end{bmatrix}, \tag{S154}$$

where

$$d_1 = b_1 - \lambda_{SO}^{1st}, \quad d_2 = b_2 + \lambda_{SO}^{1st} \tag{S155}$$

(which coincide with the definitions reported in Eq. (S128)).

III. EXTENDED $\vec{K} \cdot \vec{P}$ ANALYSIS AT THE POINT $\vec{\Gamma}$

Here we report the derivation of a 4-band (8-band including the spin) $\vec{k} \cdot \vec{p}$ Hamiltonian around $\vec{\Gamma}$, obtained considering around $\vec{\Gamma}$ the four states with energy in $\vec{\Gamma}$ equal to $-V_6$ (double degenerate), ϵ_3' and ϵ_1' (which are the states nearest to the Fermi energy and correspond to the two highest valence bands and to the two lowest conduction bands).

In order to arrive at this result, we have first diagonalized the tight-binding Hamiltonian exactly at the point $\vec{\Gamma}$. In $\vec{\Gamma}$, adopting the basis set $\mathcal{V}_1$ the Hamiltonian matrix is $\tilde{H}_0(\vec{\Gamma})$ (see Eq. (S71)). Following the first steps described in our Appendix B.3, we have first moved from the basis set $\mathcal{V}_1$ to the basis set $\mathcal{M}_2$ through the unitary transformation $\tilde{Q}_1$; as a consequence, the Hamiltonian matrix becomes $H_1(\vec{\Gamma})$ (see Eq. (S75)). Then, we have moved from the basis set $\mathcal{M}_2$ to the basis set $\mathcal{M}_3$ through the unitary transformation Q_2', obtaining the diagonalized Hamiltonian $H_2(\vec{\Gamma})$

(see Eq. (S85)). Finally, since in this case we wanted to focus on the four states with energy in $\vec{\Gamma}$ equal to $-V_6$ (double degenerate), ϵ_3' and ϵ_1', we have permuted the position of the basis states in such a way as to put these 4 states in the first positions, through a final change of basis $(R_2'')^\dagger H_2(\vec{\Gamma}) R_2'' = H_0'(\vec{\Gamma})$, where

$$R_2'' = \begin{bmatrix} 1&0&0&0&0&0&0&0\\ 0&0&0&0&1&0&0&0\\ 0&1&0&0&0&0&0&0\\ 0&0&0&0&0&1&0&0\\ 0&0&0&0&0&0&1&0\\ 0&0&0&0&0&0&0&1\\ 0&0&1&0&0&0&0&0\\ 0&0&0&1&0&0&0&0 \end{bmatrix}, \quad H_0'(\vec{\Gamma}) = \begin{bmatrix} \epsilon_1'&0&0&0&0&0&0&0\\ 0&\epsilon_3'&0&0&0&0&0&0\\ 0&0&-V_6&0&0&0&0&0\\ 0&0&0&-V_6&0&0&0&0\\ 0&0&0&0&\epsilon_2'&0&0&0\\ 0&0&0&0&0&\epsilon_4'&0&0\\ 0&0&0&0&0&0&V_6&0\\ 0&0&0&0&0&0&0&V_6 \end{bmatrix}. \tag{S156}$$

In this way we have moved from the basis set $\mathcal{M}_3$ to the basis set $\mathcal{P}_4 = \{|\tau_1\rangle, |\tau_3\rangle, |\tau_7\rangle, |\tau_8\rangle, |\tau_2\rangle, |\tau_4\rangle, |\tau_5\rangle, |\tau_6\rangle\}$.
The overall unitary matrix $\tilde{R}$ corresponding to the basis change from $\mathcal{V}_1$ to $\mathcal{P}_4$, i.e. such that $(\tilde{R})^\dagger \tilde{H}_0(\vec{\Gamma})\tilde{R} = H_0'(\vec{\Gamma})$, is

$$\tilde{R} = \tilde{Q}_1 Q_2' R_2'' = \frac{1}{\sqrt{2}} \begin{bmatrix} q_{11} & q_{33} & 0 & 0 & q_{12} & q_{34} & 0 & 0\\ 0&0&1&0&0&0&1&0\\ 0&0&0&1&0&0&0&1\\ q_{21} & q_{43} & 0 & 0 & q_{22} & q_{44} & 0 & 0\\ q_{11} & -q_{33} & 0 & 0 & q_{12} & -q_{34} & 0 & 0\\ 0&0&-1&0&0&0&1&0\\ 0&0&0&-1&0&0&0&1\\ -q_{21} & q_{43} & 0 & 0 & -q_{22} & q_{44} & 0 & 0 \end{bmatrix}. \tag{S157}$$

Then, we have obtained the 4-band $\vec{k}\cdot\vec{p}$ Hamiltonian of stanene around $\vec{\Gamma}$ performing a low-order expansion around $\vec{\Gamma}$ of the tight-binding Hamiltonian of Eq. (S43) and reducing the matrix size through projection onto the subset of states $\{|\tau_1\rangle, |\tau_3\rangle, |\tau_7\rangle, |\tau_8\rangle\}$.
More in detail, in $\tilde{H}_o(\vec{k})$ (see Eq. (S43)) we have substituted each occurrence of $\vec{k}$ with $\vec{\Gamma}+\vec{\kappa}$ (where $\vec{\kappa} = \vec{k}-\vec{\Gamma}$ represents the distance in the reciprocal space between $\vec{\Gamma}$ and $\vec{k}$); then we have performed a second-order Taylor expansion around $\vec{\kappa}=0$ (which corresponds to $\vec{k}=\vec{\Gamma}$) of all the elements of the matrix. The second-order Taylor expansion $\tilde{H}_o^{(\mathrm{exp})}(\vec{\kappa})$ of the Hamiltonian $\tilde{H}_o(\vec{k})$ can be simply performed substituting in all the elements of the matrix (see Eq. (S44)) to the exponentials $e^{i\vec{k}\cdot\vec{d}_i}$ the quantities reported in Eq. (S88). In order to project the expanded Hamiltonian $\tilde{H}_o^{(\mathrm{exp})}(\vec{\kappa})$ onto the subset of basis states $\{|\tau_1\rangle, |\tau_3\rangle, |\tau_7\rangle, |\tau_8\rangle\}$ (corresponding to the bands nearest to the Fermi energy), we have first changed the basis from $\mathcal{V}_1$ to $\mathcal{P}_4$, obtaining the matrix $H_0'^{(\mathrm{exp})}(\vec{\kappa}) = (\tilde{R})^\dagger \tilde{H}_0^{(\mathrm{exp})}(\vec{\kappa})\,\tilde{R}$. Since we have previously ordered the basis elements of $\mathcal{P}_4$ in such a way as to have the states $\{|\tau_1\rangle, |\tau_3\rangle, |\tau_7\rangle, |\tau_8\rangle\}$ in the first positions, the projection of $H_0'^{(\mathrm{exp})}(\vec{\kappa})$ onto this set of states is the 4×4 submatrix obtained from the intersection of its first 4 rows and of its first 4 columns. At the end of this analytical procedure, we obtain the following 4×4 matrix:

$$H_{\vec{\Gamma}}(\vec{\kappa}) = \begin{bmatrix} \epsilon_1' + c_1(\kappa_x^2+\kappa_y^2) & 0 & c_2\kappa_x\kappa_y & -\frac{c_2}{2}(-\kappa_x^2+\kappa_y^2)\\ 0 & \epsilon_3' + c_3(\kappa_x^2+\kappa_y^2) & -ic_4\kappa_x & -ic_4\kappa_y\\ c_2\kappa_x\kappa_y & ic_4\kappa_x & -V_6 + c_5\kappa_x^2 + c_6\kappa_y^2 & -c_7\kappa_x\kappa_y\\ -\frac{c_2}{2}(-\kappa_x^2+\kappa_y^2) & ic_4\kappa_y & -c_7\kappa_x\kappa_y & -V_6 + c_6\kappa_x^2 + c_5\kappa_y^2 \end{bmatrix} \tag{S158}$$

where

$$\begin{gathered} c_1 = \frac{a^2}{12}(-V_4\,q_{11}^2 + V_5\,2\,q_{11}\,q_{21} + V_7\,q_{21}^2)\,, \quad c_2 = \frac{a^2}{6\sqrt{2}}(V_2\,q_{11} - V_3\,q_{21})\,,\\ c_3 = \frac{a^2}{12}(V_4\,q_{33}^2 - V_7\,q_{43}^2 - V_5\,2\,q_{33}\,q_{43})\,, \quad c_4 = \frac{a}{\sqrt{6}}(V_2\,q_{33} - V_3\,q_{43})\,,\\ c_5 = \frac{a^2}{8}(V_6 - V_{pp\pi})\,, \quad c_6 = \frac{a^2}{24}(V_6 + 3\,V_{pp\pi}), \quad c_7 = \frac{a^2}{12}V_1\,. \end{gathered} \tag{S159}$$

Now let us include also the effect of the spin-orbit coupling (neglecting the Rashba term). On the basis set $\mathcal{V}_1 \otimes \{|\uparrow\rangle, |\downarrow\rangle\} = \{|s^A\uparrow\rangle, |s^A\downarrow\rangle, |p_x^A\uparrow\rangle, |p_x^A\downarrow\rangle, |p_y^A\uparrow\rangle, |p_y^A\downarrow\rangle, |p_z^A\uparrow\rangle, |p_z^A\downarrow\rangle, |s^B\uparrow\rangle, |s^B\downarrow\rangle, |p_x^B\uparrow\rangle, |p_x^B\downarrow\rangle, |p_y^B\uparrow\rangle, |p_y^B\downarrow\rangle, |p_z^B\uparrow\rangle, |p_z^B\downarrow\rangle\}$ (the operator $\otimes$ being the tensor product) the spin-orbit contribution to the Hamiltonian is

$$\tilde{H}_{SO} = \left[\begin{array}{cc} \hat{H}_{SO} & 0 \\ 0 & \hat{H}_{SO} \end{array} \right], \tag{S160}$$

where $\hat{H}_{SO}$ is defined in Eq. (S91). On the other hand, the part of Hamiltonian which does not include the spin-orbit interaction, i.e. $\tilde{H}_0(\vec{k})$, acts identically on spin-up and spin-down orbitals, and thus it can be expressed on the basis set $\mathcal{V}_1 \otimes \{|\uparrow\rangle, |\downarrow\rangle\}$ as $\tilde{H}_0(\vec{k}) \otimes I_2$ (where I_2 is the 2×2 identity matrix). Therefore, the total Hamiltonian on the basis set $\mathcal{V}_1 \otimes \{|\uparrow\rangle, |\downarrow\rangle\}$ is $\tilde{H}_0(\vec{k}) \otimes I_2 + \tilde{H}_{SO}$. We can repeat our previous treatment on this extended Hamiltonian. We first perform a second-order Taylor expansion around $\vec{\Gamma}$ (only of the first term, because the second term does not depend on $\vec{k}$). Then we move from the basis set $\mathcal{V}_1 \otimes \{|\uparrow\rangle, |\downarrow\rangle\}$ to the basis set $\mathcal{P}_4 \otimes \{|\uparrow\rangle, |\downarrow\rangle\}$ through the matrix $\tilde{R} \otimes I_2$, obtaining:

$$\begin{aligned} &(\tilde{R} \otimes I_2)^\dagger (\tilde{H}_0^{(\exp)}(\vec{\kappa}) \otimes I_2 + \tilde{H}_{SO})(\tilde{R} \otimes I_2) = \\ &= (\tilde{R} \otimes I_2)^\dagger (\tilde{H}_0^{(\exp)}(\vec{\kappa}) \otimes I_2)(\tilde{R} \otimes I_2) + (\tilde{R} \otimes I_2)^\dagger \tilde{H}_{SO}(\tilde{R} \otimes I_2) = \\ &= H_0'^{(\exp)}(\vec{\kappa}) \otimes I_2 + (\tilde{R} \otimes I_2)^\dagger \tilde{H}_{SO}(\tilde{R} \otimes I_2). \end{aligned} \tag{S161}$$

Finally, we project the resulting Hamiltonian onto the basis $\{\,|\tau_1\rangle, |\tau_3\rangle, |\tau_7\rangle, |\tau_8\rangle\} \otimes \{\,|\uparrow\rangle, |\downarrow\rangle\,\} = \{\,|\tau_1\uparrow\rangle, |\tau_1\downarrow\rangle, |\tau_3\uparrow\rangle, |\tau_3\downarrow\rangle, |\tau_7\uparrow\rangle, |\tau_7\downarrow\rangle, |\tau_8\uparrow\rangle, |\tau_8\downarrow\rangle\,\}$, obtaining:

$$H_{\vec{\Gamma}}(\vec{\kappa}) \otimes I_2 + \frac{\Delta_{SO}}{3} \left[\begin{array}{cccccccc} 0 & 0 & 0 & 0 & 0 & -q_{21} & 0 & iq_{21} \\ 0 & 0 & 0 & 0 & q_{21} & 0 & iq_{21} & 0 \\ 0 & 0 & 0 & 0 & 0 & 0 & 0 & 0 \\ 0 & 0 & 0 & 0 & 0 & 0 & 0 & 0 \\ 0 & q_{21} & 0 & 0 & 0 & 0 & -i & 0 \\ -q_{21} & 0 & 0 & 0 & 0 & 0 & 0 & i \\ 0 & -iq_{21} & 0 & 0 & i & 0 & 0 & 0 \\ -iq_{21} & 0 & 0 & 0 & 0 & -i & 0 & 0 \end{array} \right], \tag{S162}$$

where $H_{\vec{\Gamma}}(\vec{\kappa})$ is given by Eq. (S158). The second term of Eq. (S162) is obtained performing the product $(\tilde{R} \otimes I_2)^\dagger \tilde{H}_{SO}(\tilde{R} \otimes I_2)$ and considering its 8×8 submatrix given by the intersection of its first 8 rows and of its first 8 columns (since $\{\,|\tau_1\uparrow\rangle, |\tau_1\downarrow\rangle, |\tau_3\uparrow\rangle, |\tau_3\downarrow\rangle, |\tau_7\uparrow\rangle, |\tau_7\downarrow\rangle, |\tau_8\uparrow\rangle, |\tau_8\downarrow\rangle\,\}$ represent the first 8 elements of the basis set $\mathcal{P}_4 \otimes \{|\uparrow\rangle, |\downarrow\rangle\}$).

Following this procedure, we have neglected the coupling which exists between the 8 states on which we have performed our projection and the other 8 states that we have discarded (as we have seen, only the Hamiltonian without spin-orbit in $\vec{k} = \vec{\Gamma}$ is diagonal on the basis $\mathcal{P}_4 \otimes \{|\uparrow\rangle, |\downarrow\rangle\}$). The effect of this interaction can be partially recovered adopting the method described in the Appendix B of Ref. [1]. We can rewrite the matrix $H_0'^{(\exp)}(\vec{\kappa}) \otimes I_2 + (\tilde{R} \otimes I_2)^\dagger \tilde{H}_{SO}(\tilde{R} \otimes I_2)$ of Eq. (S161) (where the first term is the Hamiltonian without spin-orbit coupling and the second term is the spin-orbit contribution) in the form

$$\left[\begin{array}{cc} H_\pi & H_n \\ H_n^\dagger & H_\sigma \end{array} \right], \tag{S163}$$

where the 8×8 matrix H_π is given by the intersection of the first 8 rows and of the first 8 columns (i.e., the rows and the columns corresponding to the states considered in our projection) of the Hamiltonian of Eq. (S161), the 8×8 matrix H_n is given by the intersection of its first 8 rows and of its 9th-to-16th columns, the 8×8 matrix $H_n^\dagger$ is given by the intersection of its 9th-to-16th rows and of its first 8 columns, and the 8×8 matrix H_σ is given by the intersection of its 9th-to-16th rows and of its 9th-to-16th columns. Let us define ϵ'_{av} the average of the eigenvalues of $H_{\vec{\Gamma}}(\vec{0})$, i.e. $\epsilon'_{av} = (\epsilon'_1 + \epsilon'_3 - V_6 - V_6)/4$. Since the eigenvalues of H_π are near ϵ'_{av}, the eigenvalues of H_σ are far from it, and H_n is much smaller than $H_\sigma - \epsilon'_{av} I_8$, following the procedure described in the Appendix B of Ref. [1] we can approximate the Hamiltonian of Eq. (S161) near ϵ'_{av} with $H_\pi - H_n(H_\sigma - \epsilon'_{av} I_8)^{-1} H_n^\dagger$ (where I_8 is the 8×8 identity matrix). The matrix H_π represents the projection of Eq. (S161) on the first 8 states of the basis $\mathcal{P}_4 \otimes \{|\uparrow\rangle, |\downarrow\rangle\}$, i.e. exactly Eq. (S162). Instead, the term $-H_n(H_\sigma - \epsilon'_{av} I_8)^{-1} H_n^\dagger$ gives rise to an effective second-order spin-orbit contribution. Let us approximate [1] the Hamiltonian of Eq. (S161) with its value in $\vec{\kappa} = 0$ (i.e. in $\vec{k} = \vec{\Gamma}$); in this

case its part without spin-orbit is the diagonal matrix $H_0'(\vec{\Gamma}) \otimes I_2$. Therefore, the matrix H_n derives only from the spin-orbit part of Eq. (S161) and is given by the intersection of the first 8 rows and of the 9th-to-16th columns of $(\tilde{R} \otimes I_2)^\dagger \tilde{H}_{SO} (\tilde{R} \otimes I_2)$. Instead, in H_σ the contribution of the Hamiltonian without spin-orbit dominates on that of spin-orbit and we can approximate H_σ with the intersection of the 9th-to-16th rows and of the 9th-to-16th columns of $H_0'(\vec{\Gamma}) \otimes I_2$. Performing the calculation, we obtain the following quantity:

$$H_{SO}^{2nd} = \begin{bmatrix} 0 & 0 & 0 & 0 & 0 & 0 & 0 & 0 \\ 0 & 0 & 0 & 0 & 0 & 0 & 0 & 0 \\ 0 & 0 & b_3 & 0 & 0 & 0 & 0 & 0 \\ 0 & 0 & 0 & b_3 & 0 & 0 & 0 & 0 \\ 0 & 0 & 0 & 0 & b_4 & 0 & ib_4 & 0 \\ 0 & 0 & 0 & 0 & 0 & b_4 & 0 & -ib_4 \\ 0 & 0 & 0 & 0 & -ib_4 & 0 & b_4 & 0 \\ 0 & 0 & 0 & 0 & 0 & ib_4 & 0 & b_4 \end{bmatrix}, \tag{S164}$$

with

$$b_3 = \left(\frac{\Delta_{SO}}{3}\right)^2 \frac{2\, q_{43}^2}{\epsilon'_{av} - V_6}, \quad b_4 = \left(\frac{\Delta_{SO}}{3}\right)^2 \frac{q_{22}^2}{\epsilon'_{av} - \epsilon'_2}, \quad \epsilon'_{av} = \frac{\epsilon'_1 + \epsilon'_3 - V_6 - V_6}{4}. \tag{S165}$$

Adding Eq. (S164) to Eq. (S162), we obtain the Hamiltonian

$$H_{\vec{\Gamma}}(\vec{\kappa}) \otimes I_2 + \begin{bmatrix} 0 & 0 & 0 & 0 & 0 & -d_5 & 0 & id_5 \\ 0 & 0 & 0 & 0 & d_5 & 0 & id_5 & 0 \\ 0 & 0 & d_3 & 0 & 0 & 0 & 0 & 0 \\ 0 & 0 & 0 & d_3 & 0 & 0 & 0 & 0 \\ 0 & d_5 & 0 & 0 & d_4 & 0 & -id_6 & 0 \\ -d_5 & 0 & 0 & 0 & 0 & d_4 & 0 & id_6 \\ 0 & -id_5 & 0 & 0 & id_6 & 0 & d_4 & 0 \\ -id_5 & 0 & 0 & 0 & 0 & -id_6 & 0 & d_4 \end{bmatrix}, \tag{S166}$$

where

$$d_3 = b_3, \quad d_4 = b_4, \quad d_5 = \frac{\Delta_{SO}}{3} q_{21}, \quad d_6 = \frac{\Delta_{SO}}{3} - b_4. \tag{S167}$$

The $\vec{k} \cdot \vec{p}$ parameters obtained from the tight-binding ones (in particular, the NNTB parameters of Table 1) are reported in Table SI. In order to compute these values, we have exploited the Eqs. (S108), (S118), (S77), (S78), (S79), (S81), (S82), (S83), (S72), (S159), (S123), (S126), (S128), (S165), and (S167). Moreover, remembering that in Eq. (S43) we have subtracted $\epsilon_p = 1.7747$ eV to all the elements on the diagonal and that this shift survives all the basis changes we have previously performed, now we have added back $\epsilon_p = 1.7747$ eV to the elements located on the diagonal of the $\vec{k} \cdot \vec{p}$ Hamiltonians. This means that we have added $\epsilon_p = 1.7747$ eV to ϵ_1, ϵ'_1 and ϵ'_3, while we have subtracted $\epsilon_p = 1.7747$ eV to V_6 (because $-V_6$ appears on the diagonal of Eq. (S158)).

The $\vec{k} \cdot \vec{p}$ dispersion relations are obtained finding the eigenvalues of the $\vec{k} \cdot \vec{p}$ Hamiltonians (Eqs. (S117), (S127), (S144), (S154), (S158), (S166)). In Fig. S1 we show the comparison between the nearest-neighbor tight-binding bands and the $\vec{k} \cdot \vec{p}$ dispersion relations computed around the $\vec{K}$ and $\vec{\Gamma}$ points, both neglecting and including the spin-orbit coupling effect in the calculation.

Once we have derived the analytical form of the $\vec{k} \cdot \vec{p}$ dispersion relations, it is also useful to obtain the $\vec{k} \cdot \vec{p}$ parameters (ϵ_1, γ, ϵ'_1, ϵ'_3, V_6, c_1, c_2, c_3, c_4, c_5, c_6, c_7, d_1, d_2, d_3, d_4, d_5, d_6) directly fitting the DFT dispersion relations in the regions around the points $\vec{K}$ (or $\vec{K}'$) and $\vec{\Gamma}$, in such a way as to improve their local accuracy. We have derived these values following the same method described in our Appendix B.4 (in this case, in Eq. (S100) the number N_b near $\vec{K}$ is 2 without spin-orbit and 4 with spin-orbit, while near $\vec{\Gamma}$ it is 4 without spin-orbit and 8 with spin-orbit).

The values of the $\vec{k} \cdot \vec{p}$ parameters that we have obtained at the end of this procedure are reported in Table SII.

In Fig. S2 we show the comparison between the DFT bands and the $\vec{k} \cdot \vec{p}$ dispersion relations obtained, using these values for the $\vec{k} \cdot \vec{p}$ parameters, around the $\vec{K}$ and $\vec{\Gamma}$ points, both neglecting and including the spin-orbit coupling effect in the calculation.

If we are interested in the dispersion relations in the presence of a magnetic field $\vec{B}$, we can simply substitute the coordinates κ_ℓ (with $\ell = x, y$) with $\kappa_\ell + (e/\hbar) A_\ell$ inside our $\vec{k} \cdot \vec{p}$ Hamiltonians (Eqs. (S117), (S127), (S144), (S154), (S158), (S166)). Here, e is the modulus of the electron charge, $\hbar$ is the reduced Planck constant, and A_ℓ is the ℓ-th coordinate of the vector potential $\vec{A}$ (chosen in such a way that $\vec{B} = \vec{\nabla} \times \vec{A}$).

TABLE SI. Values of the $\vec{k}\cdot\vec{p}$ parameters obtained from the nearest-neighbor tight-binding parameters. A $\vec{k}\cdot\vec{p}$ description with 4 bands near $\vec{K}$ and 8 bands near $\vec{\Gamma}$ is considered.

$\vec{k}\cdot\vec{p}$ parameter	value obtained from NNTB parameters
ϵ_1	$3.1749\cdot 10^{-2}$ eV
γ	$2.9001\cdot 10^{-1}$ eV·nm
ϵ_1'	2.3056 eV
ϵ_3'	$5.0316\cdot 10^{-1}$ eV
V_6	$3.9049\cdot 10^{-1}$ eV
c_1	$-2.9729\cdot 10^{-2}$ eV·nm^2
c_2	$-3.2820\cdot 10^{-2}$ eV·nm^2
c_3	$-2.3078\cdot 10^{-2}$ eV·nm^2
c_4	$6.6487\cdot 10^{-1}$ eV·nm
c_5	$7.8477\cdot 10^{-2}$ eV·nm^2
c_6	$1.2379\cdot 10^{-3}$ eV·nm^2
c_7	$-7.7239\cdot 10^{-2}$ eV·nm^2
d_1	$-5.3937\cdot 10^{-2}$ eV
d_2	$4.2190\cdot 10^{-2}$ eV
d_3	$-2.2850\cdot 10^{-2}$ eV
d_4	$8.8695\cdot 10^{-5}$ eV
d_5	$2.2185\cdot 10^{-1}$ eV
d_6	$2.2391\cdot 10^{-1}$ eV

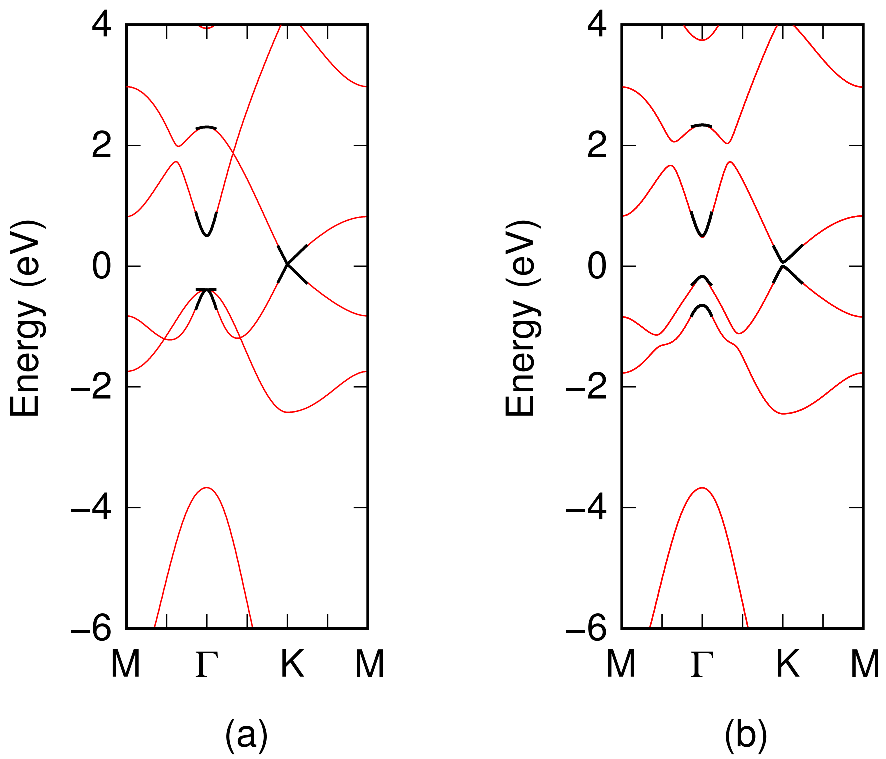

FIG. S1. Dispersion relations of stanene, obtained neglecting (a) and considering (b) spin-orbit interaction. With the thin red line we show the nearest-neighbor tight-binding bands, while with the thick black line we report the $\vec{k}\cdot\vec{p}$ dispersion relations obtained with the parameters of Table SI.

TABLE SII. Values of the $\vec{k}\cdot\vec{p}$ parameters obtained fitting the DFT dispersion relations. A $\vec{k}\cdot\vec{p}$ description with 4 bands near $\vec{K}$ and 8 bands near $\vec{\Gamma}$ is considered.

$\vec{k}\cdot\vec{p}$ parameter	value fitted from DFT
ϵ_1	$-2.6346\cdot 10^{-4}$ eV
γ	$2.9360\cdot 10^{-1}$ eV·nm
ϵ_1'	1.4406 eV
ϵ_3'	$1.7378\cdot 10^{-1}$ eV
V_6	$4.6849\cdot 10^{-1}$ eV
c_1	$7.4819\cdot 10^{-3}$ eV·nm^2
c_2	$9.2618\cdot 10^{-4}$ eV·nm^2
c_3	$1.2286\cdot 10^{-4}$ eV·nm^2
c_4	$7.4054\cdot 10^{-1}$ eV·nm
c_5	$6.5772\cdot 10^{-2}$ eV·nm^2
c_6	$-8.0303\cdot 10^{-2}$ eV·nm^2
c_7	$-1.3981\cdot 10^{-1}$ eV·nm^2
d_1	$1.6876\cdot 10^{-2}$ eV
d_2	$1.0202\cdot 10^{-1}$ eV
d_3	$1.5447\cdot 10^{-3}$ eV
d_4	$2.4556\cdot 10^{-1}$ eV
d_5	$3.8460\cdot 10^{-1}$ eV
d_6	$1.6716\cdot 10^{-1}$ eV

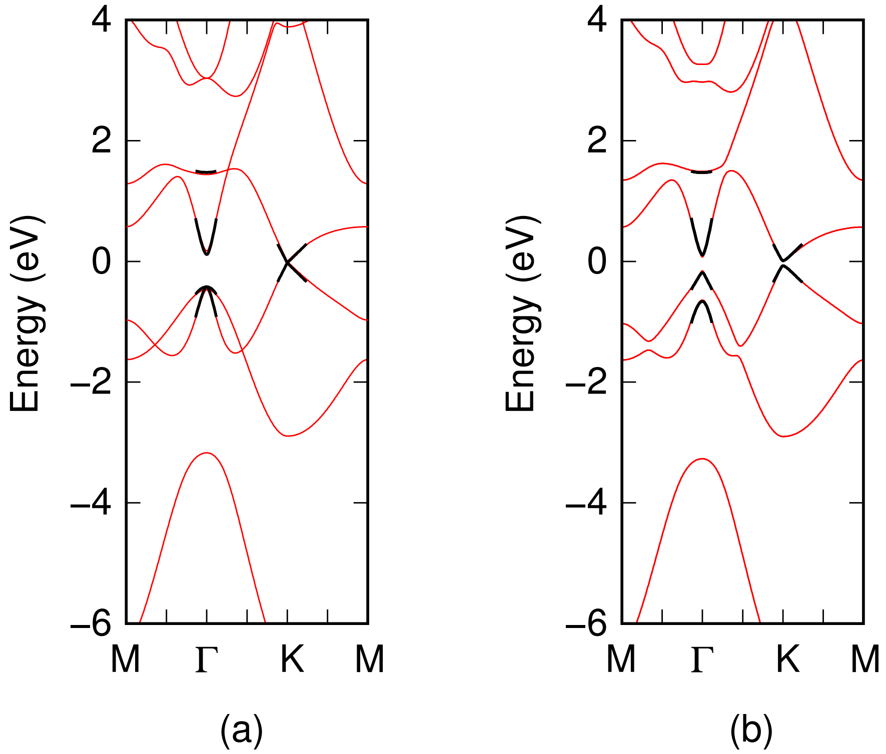

FIG. S2. Dispersion relations of stanene, obtained neglecting (a) and considering (b) spin-orbit interaction. With the thin red line we show the DFT bands, while with the thick black line we report the $\vec{k}\cdot\vec{p}$ dispersion relations obtained with the parameters of Table SII.

REFERENCES

[1] C.-C. Liu, H. Jiang, and Y. Yao. Low-energy effective Hamiltonian involving spin-orbit coupling in silicene and two-dimensional germanium and tin. *Phys. Rev. B*, 84(19):195430, 2011.
